# Supplementary material for: The prefrontal cortex encodes task-identity information and flexibly adjusts its sensory processes as a function of the specific ongoing task
Source: PLoS Biol. 2025 Aug 26;23(8):e3003353. doi: 10.1371/journal.pbio.3003353 (PMC12463330; doi:10.1371/journal.pbio.3003353)
Supplement: S1 File — Task-related selectivity might be accounted for by changes in the signal along the recording session, resulting in possibly different firing rate fluctuations not related with the cognitive aspects of the tasks. To reduce this effect, we decided to work with normalized activity with respect to the baseline in each trial. Furthermore, to prove that our selectivity results are not driven by systematic firing rate fluctuations driven by the time in the session, we conducted a supplementary analysis in which we extracted the task and position selectivity by selecting trials randomly along the recording session. In this way, our data did not contain any temporal structure given by the time-course along the recording session, nor information about task or position. We performed this procedure 100 times and computed the % of position, task, mixed selectivity and non-selective channels. The 95% confidence interval for both task and position are below 2.5% for all categories. We conducted a second analysis in which we computed the % of position, task, mixed selectivity and non-selective channels over a randomly selected subset of 10 trials for each possible trial category. We repeated this procedure 100 times. The outcome of this analysis is very close to that reported in main Fig 2. These supplementary analyses are captured by the figure below. Over all, these additional analyses indicate that the reported results in main Fig 2 are robust to neuronal variability as well as to possible temporal structure in the task. Barplot corresponding to the proportion of MUAs (left) or SUAs (right) tuned to only position (red), task (blue) and both (additive mixed-selectivity; yellow; mixed selectivity with interaction; green, mean ± s.e. computed over 100 repetitions of over a random selection of 10 trials for each possible trial category). Non-selective MUAs/SUAs are plotted in gray. Black dashed line corresponds to the 95% CI computed independently for each category of cells. Note th [file pbio.3003353.s001.pdf]

Supplementary material M1: Stability of MUA/SUA selectivity across trials. Task-related selectivity might be accounted for by changes in the signal along the recording session, resulting in possibly different firing rate fluctuations not related with the cognitive aspects of the tasks. To reduce this effect, we decided to work with normalized activity with respect to the baseline in each trial. Furthermore, to prove that our selectivity results are not driven by systematic firing rate fluctuations driven by the time in the session, we conducted a supplementary analysis in which we extracted the task and position selectivity by selecting trials randomly along the recording session. In this way, our data did not contain any temporal structure given by the time-course along the recording session, nor information about task or position. We performed this procedure 100 times and computed the % of position, task, mixed selectivity and non-selective channels. The 95% confidence interval for both task and position are below 2.5% for all categories. We conducted a second analysis in which we computed the % of position, task, mixed selectivity and non-selective channels over a randomly selected subset of 10 trials for each possible trial category. We repeated this procedure 100 times. The outcome of this analysis is very close to that reported in main figure 2. These supplementary analyses are captured by the figure below. Over all, these additional analyses indicate that the reported results in main figure 2 are robust to neuronal variability as well as to possible temporal structure in the task.

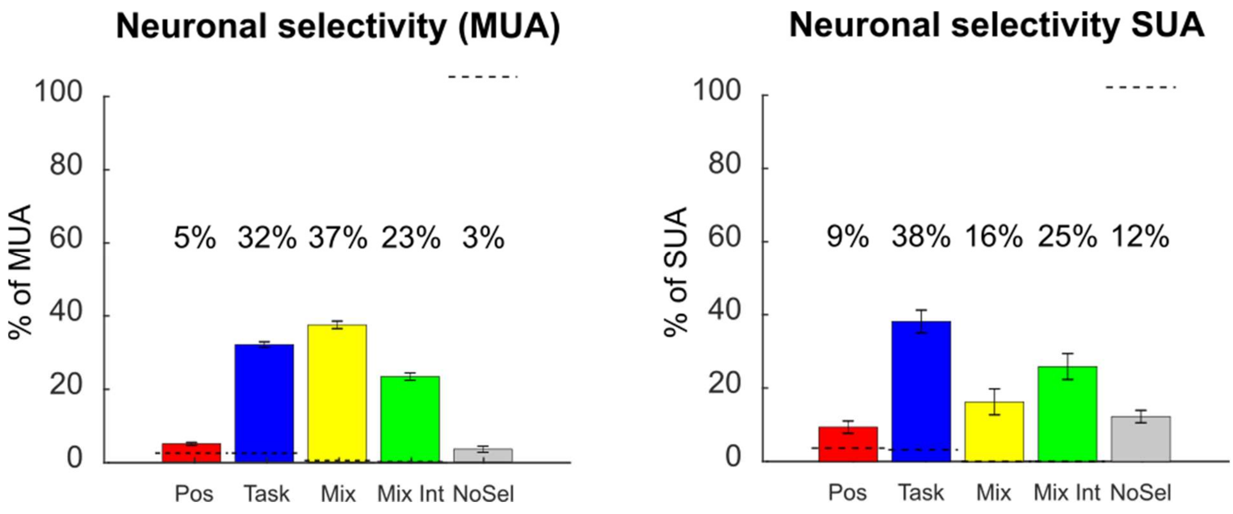

Barplot corresponding to the proportion of MUAs (left) or SUAs (right) tuned to only position (red), task (blue) and both (additive mixed-selectivity; yellow; mixed selectivity with interaction; green, mean +/- s.e. computed over 100 repetitions of over a random selection of 10 trials for each possible trial category). Non-selective MUAs/SUAs are plotted in grey. Black dashed line corresponds to the 95% CI computed independently for each category of cells. Note that the average proportion of non-selective MUAs/SUAs is very low relative to the 95% CI estimated from a random permutation procedure.
