# Supplementary material for: The prefrontal cortex encodes task-identity information and flexibly adjusts its sensory processes as a function of the specific ongoing task
Source: PLoS Biol. 2025 Aug 26;23(8):e3003353. doi: 10.1371/journal.pbio.3003353 (PMC12463330; doi:10.1371/journal.pbio.3003353)
Supplement: S2 File — In contrast with other methods such as decoding or PCA, the analysis of neuronal variability that we implemented in this work relies on demixed principal component analysis (dPCA, see below). This method has been shown in the literature not to be affected by the drift in the signal (Kobak and colleagues [32]). Indeed, it extracts the neuronal variance associated to specific parameters (here, task or position), and separates this neuronal variance from the parameter-independent variance. Monkeys performed each task for 20 min or more. Thus, drift could happen both within any task and between tasks. These changes in neuronal responses were captured in the parameter-independent variance. We further validate that drift did not account for the main results presented in Figs 4 and 5, as follows. We performed task-dPCA on each of the independent sessions. For each session and each task, we projected the population activity on the first task-component. We then plotted the average of this activity not as a function of task, but as a function of task order in the recording session. While the memory guided saccade task was always the last task in the session, half of the sessions started with the Exo task and the other half started with the Endo task. We found that there was no statistically significant difference between the distributions of average projected activity recorded during the first and second task onto the first dPC (figure below). Thus, the task-dPCA component cannot distinguish between first and second task in the session, while is can distinguish between Endo and Exo attentional tasks. Normalized firing rate of projected population activity on the first task-component of the dPCA per session, computed independently for the first task in the session (50% Exo, 50% Endo), the second task in the session (50% Exo, 50% Endo) and the last task in the session (100% memory-guided saccade task; 1-way non-parametric ANOVA: p = 5.0396e − 14; post-hoc Bonferroni test, task [file pbio.3003353.s002.pdf]

Supplementary material M2: Despite the precautions taken to stabilize the electrodes after their insertion in the brain, and trial-based baseline correction, a drift related to physiological parameters, could still impact neuronal recordings along the session. In contrast with other methods such as decoding or PCA, the analysis of neuronal variability that we implemented in this work relies on demixed principal component analysis (dPCA, see below). This method has been shown in the literature not to be affected by the drift in the signal (Kobak et al., 2016). Indeed, it extracts the neuronal variance associated to specific parameters (here, task or position), and separates this neuronal variance from the parameter-independent variance. Monkeys performed each task for 20 minutes or more. Thus, drift could happen both within any task and between tasks. These changes in neuronal responses were captured in the parameter-independent variance.

We further validate that drift did not account for the main results presented in Figures 4 and 5, as follows. We performed task-dPCA on each of the independent sessions. For each session and each task, we projected the population activity on the first task-component. We then plotted the average of this activity not as a function of task, but as a function of task order in the recording session. While the memory guided saccade task was always the last task in the session, half of the sessions started with the Exo task and the other half started with the Endo task. We found that there was no statistically significant difference between the distributions of average projected activity recorded during the first and second task onto the first dPC (figure below). Thus, the task-dPCA component cannot distinguish between first and second task in the session, while is can distinguish between Endo and Exo attentional tasks.

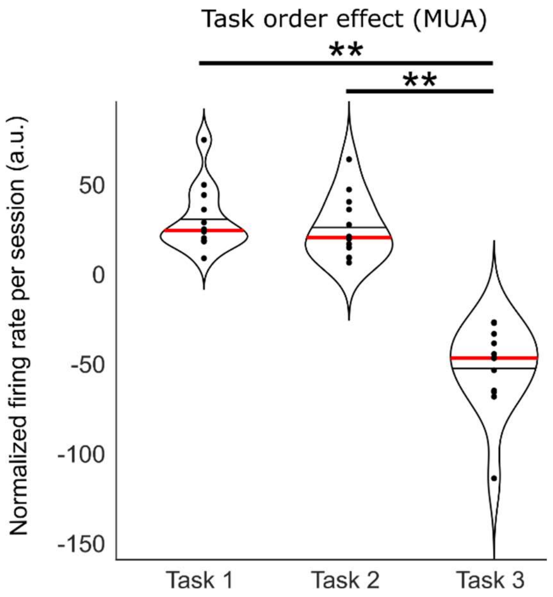

Normalized firing rate of projected population activity on the first task-component of the dPCA per session, computed independently for the first task in the session (50% Exo, 50% Endo), the second task in the session (50% Exo, 50% Endo) and the last task in the session (100% memory-guided saccade task; 1-way non-parametric ANOVA: $p = 5.0396e-14$; post-hoc Bonferroni test, task 1 vs. task 2: $p = 1$, task 1 vs. task 3: $p = 6.2112e-13$, task 2 vs. task 3: $p = 2.2593e-12$). Please note that this analysis cannot be performed on SUA data as it requires dPCA to be performed at the session level.
