# Supplementary figures and images for: The prefrontal cortex encodes task-identity information and flexibly adjusts its sensory processes as a function of the specific ongoing task
Source: PLoS Biol. 2025 Aug 26;23(8):e3003353. doi: 10.1371/journal.pbio.3003353 (PMC12463330; doi:10.1371/journal.pbio.3003353)

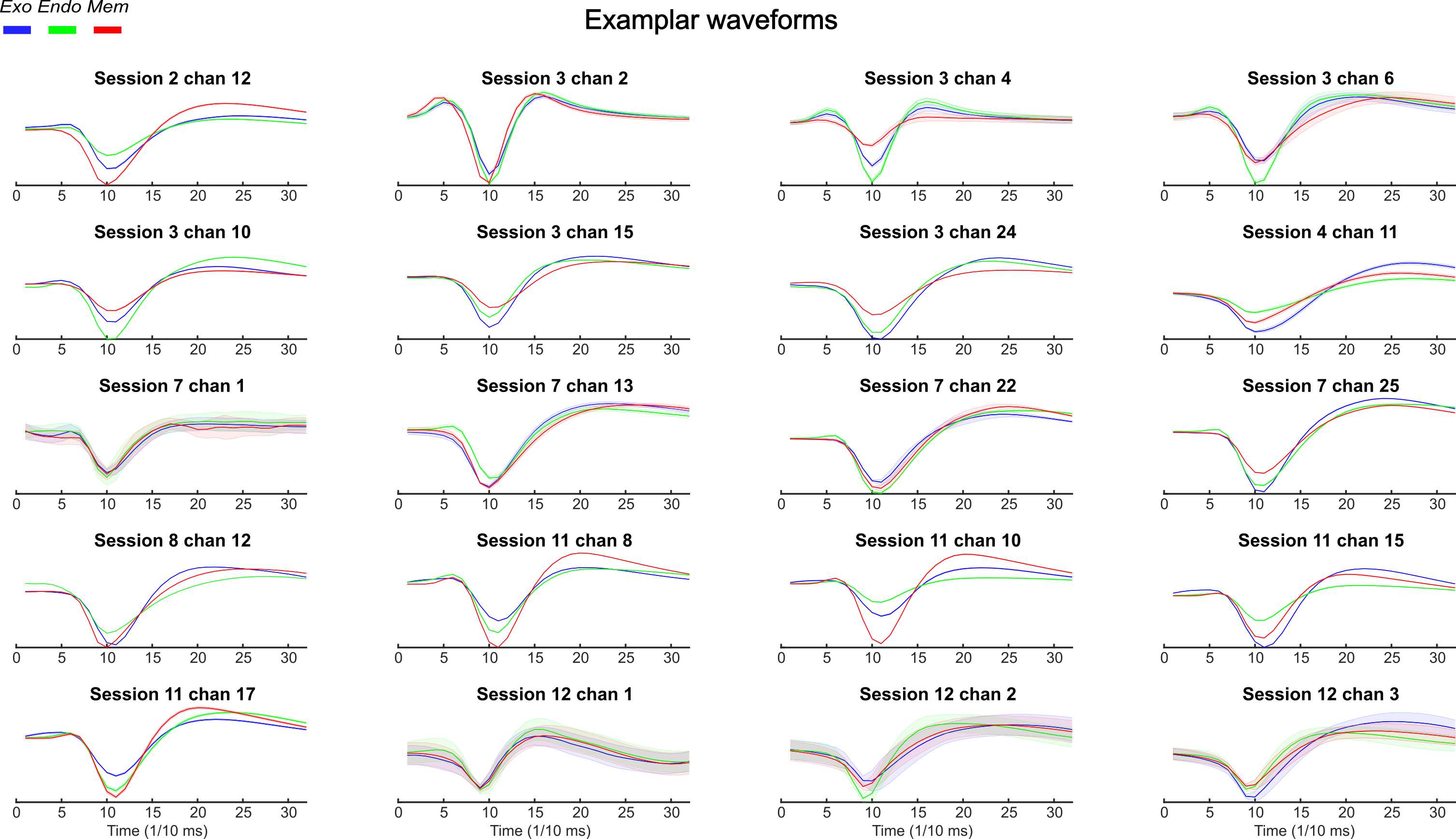

Supplement: S1 Fig — Mean (±s.e.) of each waveform is presented per task (Blue: Exogenous attentional task; Green: Endogenous attentional task; Red: Memory guided saccade task). Data and code to S1 Fig can be found at https://osf.io/z8eh9/. (TIFF) [file pbio.3003353.s003.tiff]

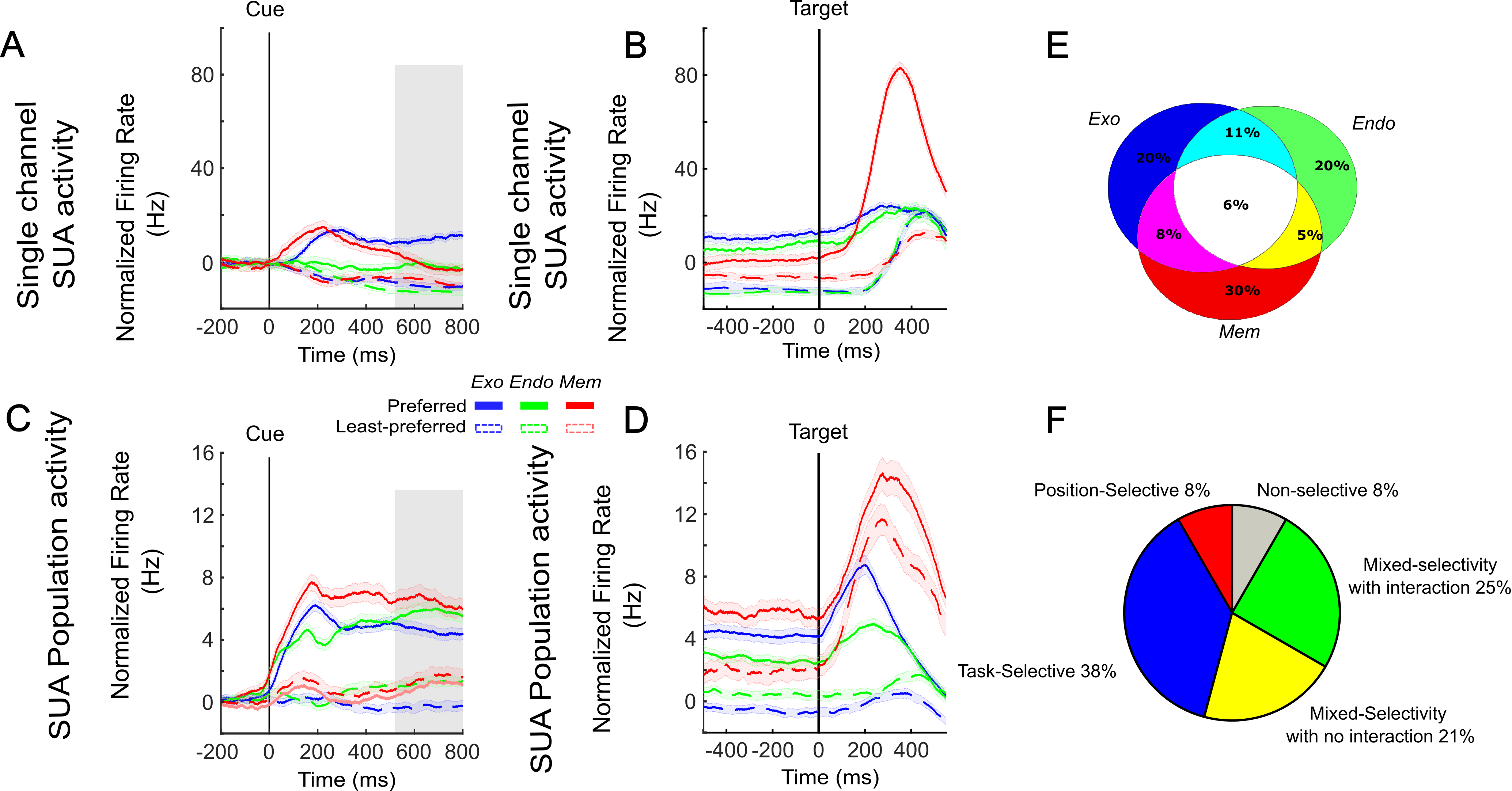

Supplement: S2 Fig — The data were baseline corrected (−500 to −200 ms before cue onset) on individual trial and channel. Gray box corresponds to the delay period during which neuronal activities were analyzed for subplots C and D. (B) Firing rate of a representative SUA (same as in A) aligned on the target (500 ms before the target onset to 500 ms after the target onset). All else as in A. (C) Mean firing rate of the SUA neuronal population in the three tasks aligned on cue presentation (200 ms before the cue onset to 1,000 ms after the cue onset) in the exogenous task (blue), the endogenous task (green) and the memory guided saccade task (red) for preferred (full line) and non-preferred (dash line) position. The data were baseline corrected (−500 to −200 ms before cue onset) on individual trial and channel. Gray box corresponds to the delay period during which neuronal activities were analyzed for subplots C and D. (D) Firing rate of the SUA neuronal population (same as in C) aligned on the target (500 ms before the target onset to 500 ms after the target onset). All else as in C. (E) Venn diagram representing the proportion of cells showing a spatial tuning in each of the three tasks. (F) Pie chart corresponding to the proportion of neurons tuned to only position (red), task (blue) and both (Additive mixed-selectivity; yellow; Mixed-selectivity with interaction; green). Non-selective neurons are plotted in gray. Wilcoxon ranksum test was performed between baseline (−100 to 0 before the cue onset) and time interval (800–900 ms after the cue onset) for each channel and position across trials. A non-parametric 2-Way-ANOVA was performed on selective channels with Bonferroni–Holm correction on spatial and task factor on time interval from 600 to 900 ms after the cue onset (p < 0.01). Data and code to S2 Fig can be found at https://osf.io/z8eh9/. (TIFF) [file pbio.3003353.s004.tiff]

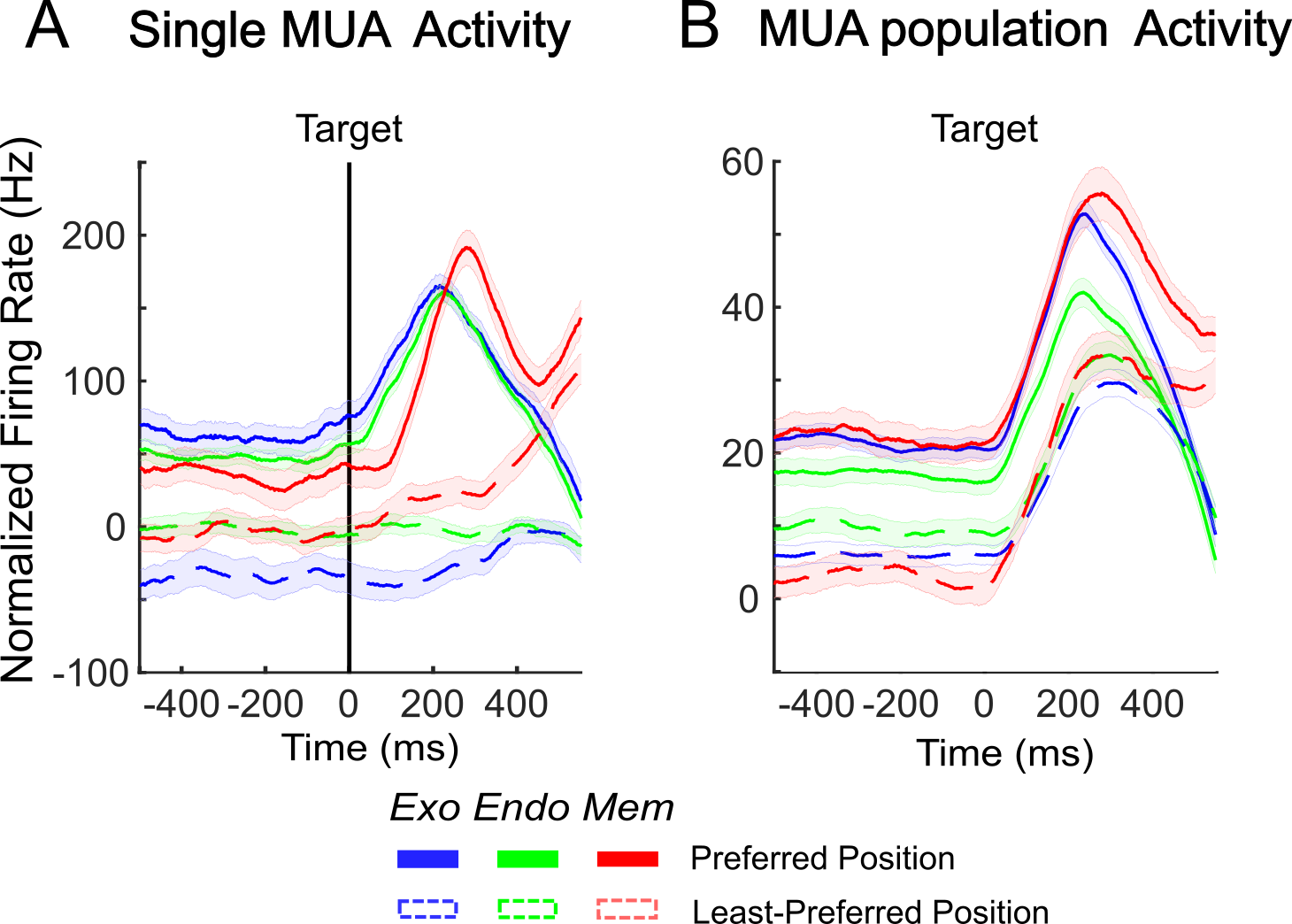

Supplement: S3 Fig — The data were baseline corrected (−500 to −200 ms before cue onset) on individual trials and channels. (B) Mean firing rate across the neuronal population (MUA) aligned on the target (−400 ms before target onset to 600 ms after target onset) as a function of tasks and positions (same as in A). The data were baseline corrected (−500 to −200 ms before cue onset) on individual trials and channels. Data and code to S3 Fig can be found at https://osf.io/z8eh9/. (TIFF) [file pbio.3003353.s005.tiff]

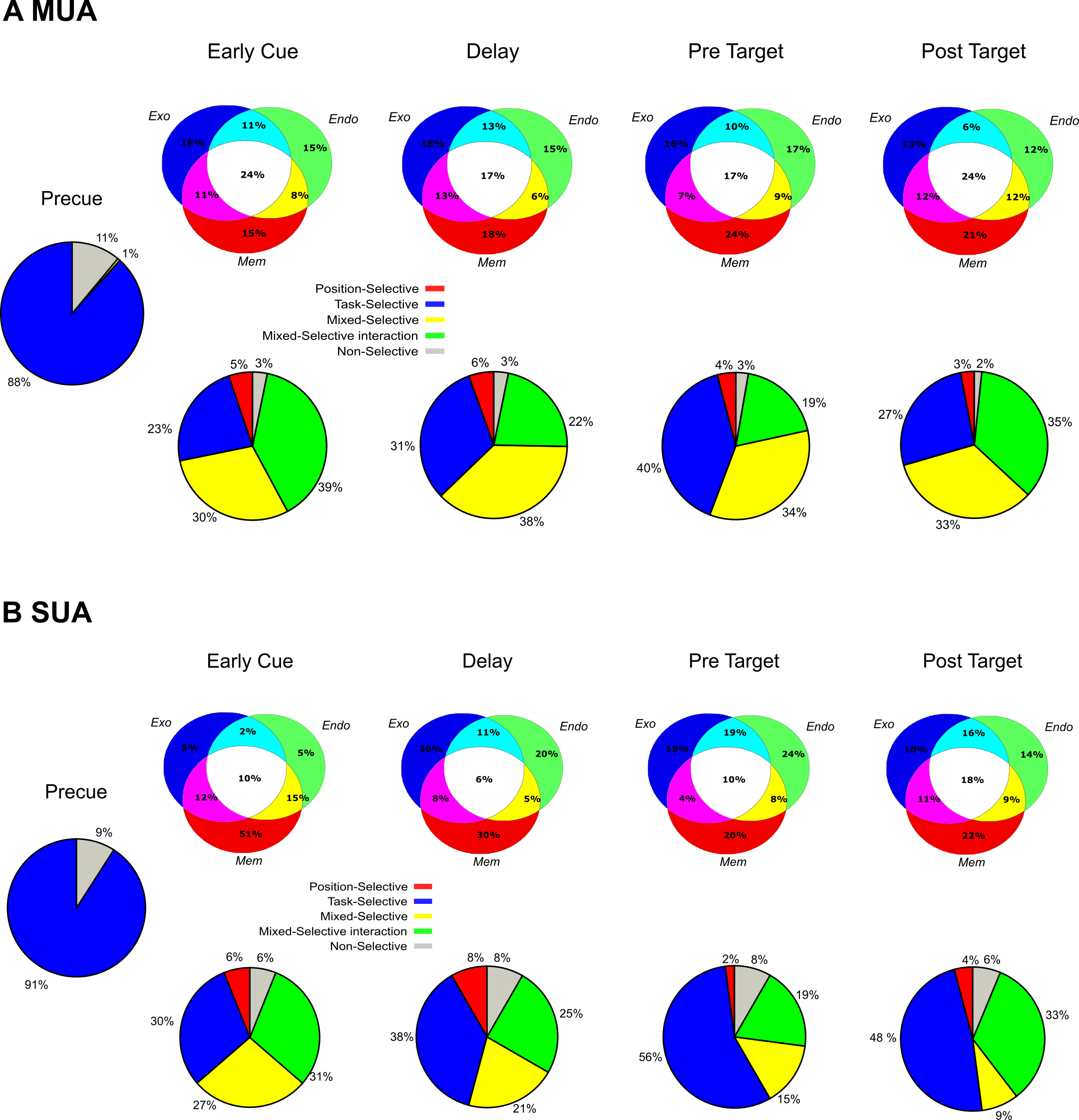

Supplement: S5 Fig — Bottom row: pie chart corresponding to the proportion of neurons tuned to only position (red), task (blue) or both (mixed-selectivity; yellow) during different periods in the trial (Precue: from 0 to 500 ms before the cue onset; Early Cue: from 0 to 500 ms after the cue onset; Delay: from 500 to 1,000 ms after the cue onset; Pre Target: from −500 to 0 ms before the target onset; and Target: from 0 to 500 ms after the target onset). Proportion of non-selective neurons is plotted in gray. This figure complements Fig 2C and 2D. (B) SUA: all as in A. This figure complements S2E and S2F Fig. Data and code to S5 Fig can be found at https://osf.io/z8eh9/. (TIFF) [file pbio.3003353.s007.tiff]

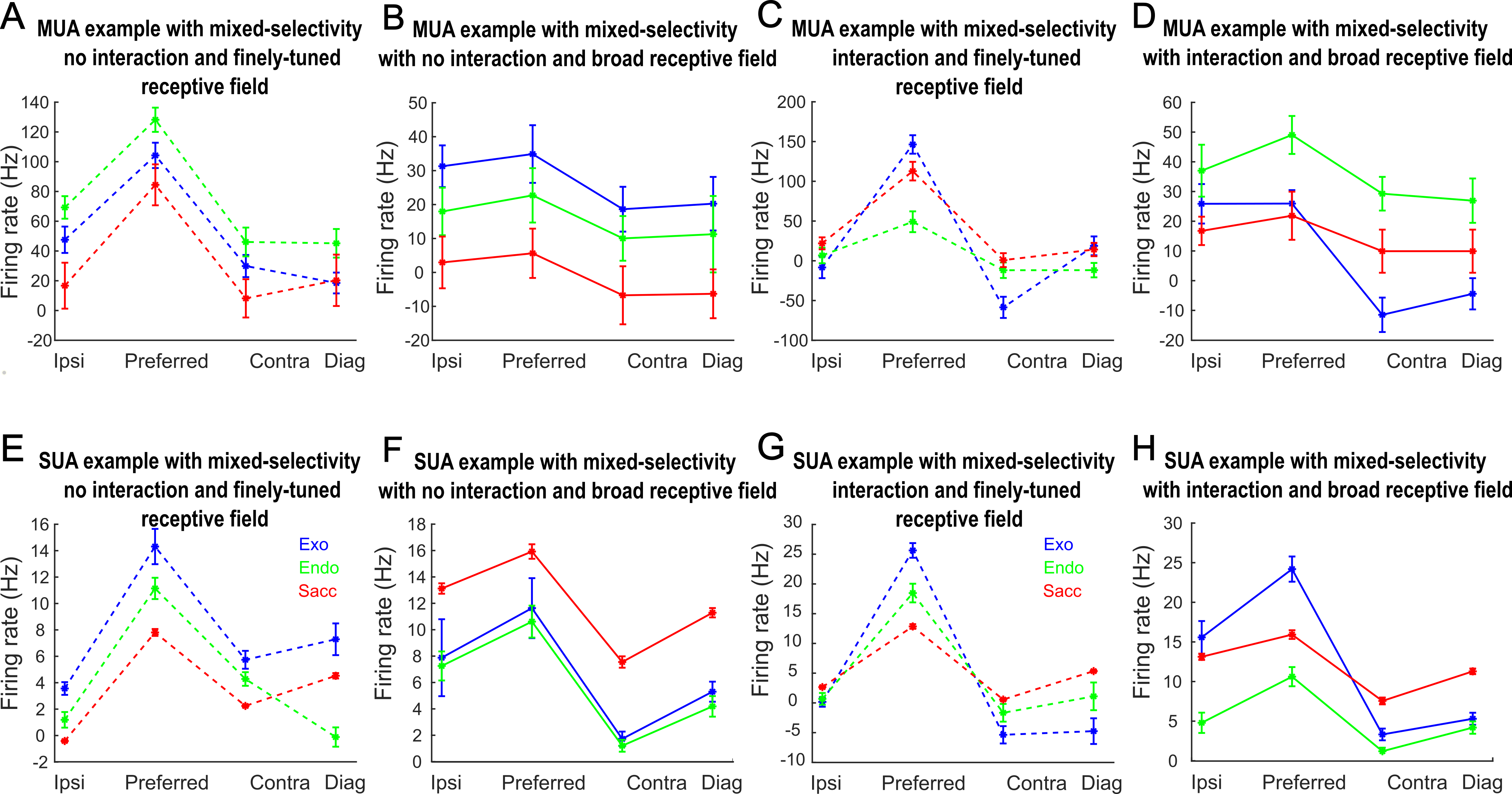

Supplement: S6 Fig — (A) MUA example, with mixed-selectivity without interaction, and finely-tuned receptive field (Exo: blue, Endo: green, Sacc: red). (B) MUA example, with mixed-selectivity without interaction, and board receptive field. (C) MUA example, with mixed-selectivity with interaction, and finely-tuned receptive field (Exo: blue, Endo: green, Sacc: red). (D) MUA example, with mixed-selectivity with interaction, and board receptive field. (E)–(H) Same as (A)–(D) for SUA example. Data and code to S6 Fig can be found at https://osf.io/z8eh9/. (TIFF) [file pbio.3003353.s008.tiff]

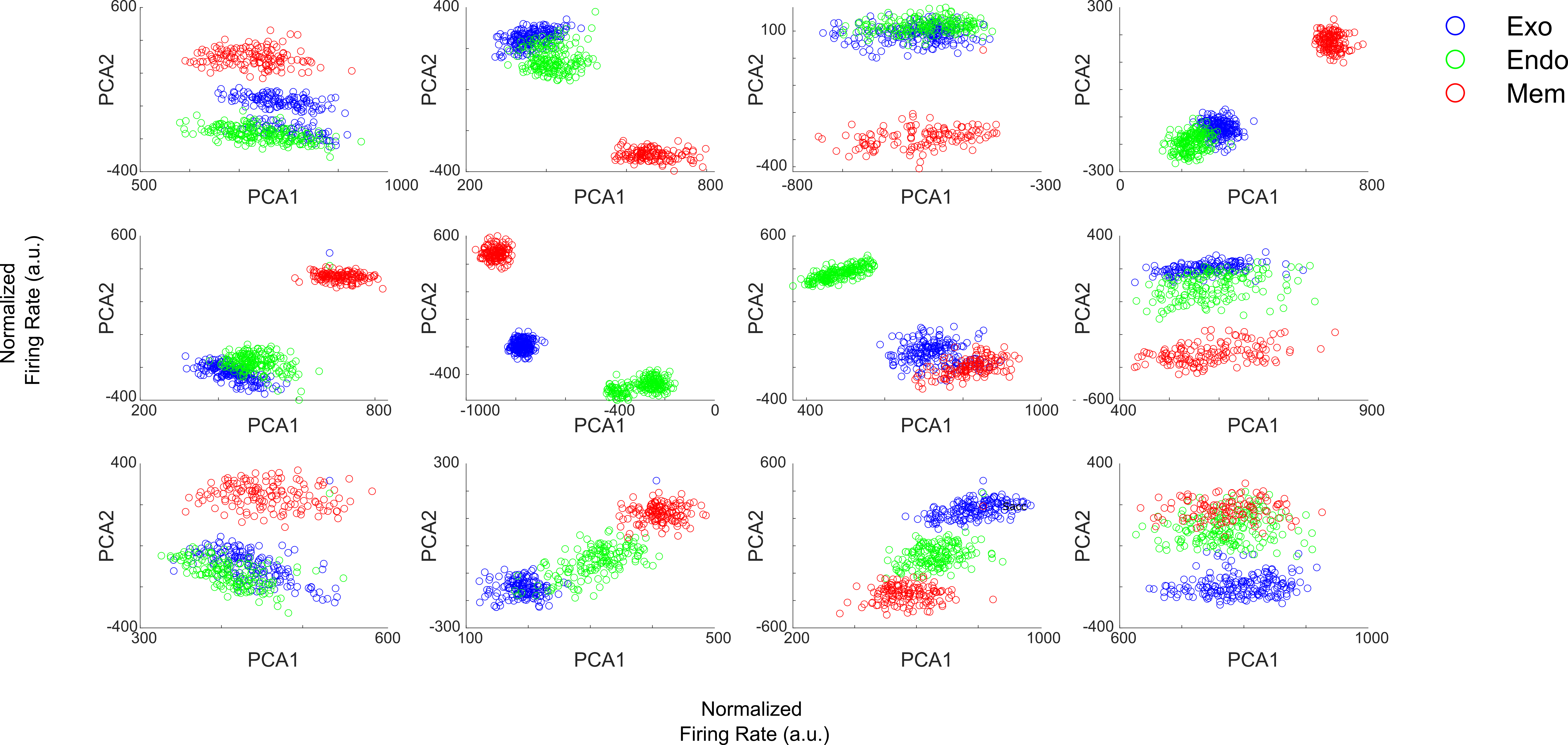

Supplement: S7 Fig — Each point corresponds to the projection of the activity in each trial (averaged in the time interval −200 to 0 ms pre Cue, each circle thus corresponds to one trial) in each task (Exogenous task, blue; Endogenous task; green; Memory saccade task, red). Each plot corresponds to a different session. Plots 1–7 are from monkey 1 and plots 8–12 are from monkey 2. Data and code to S7 Fig can be found at https://osf.io/z8eh9/. (TIFF) [file pbio.3003353.s009.tiff]

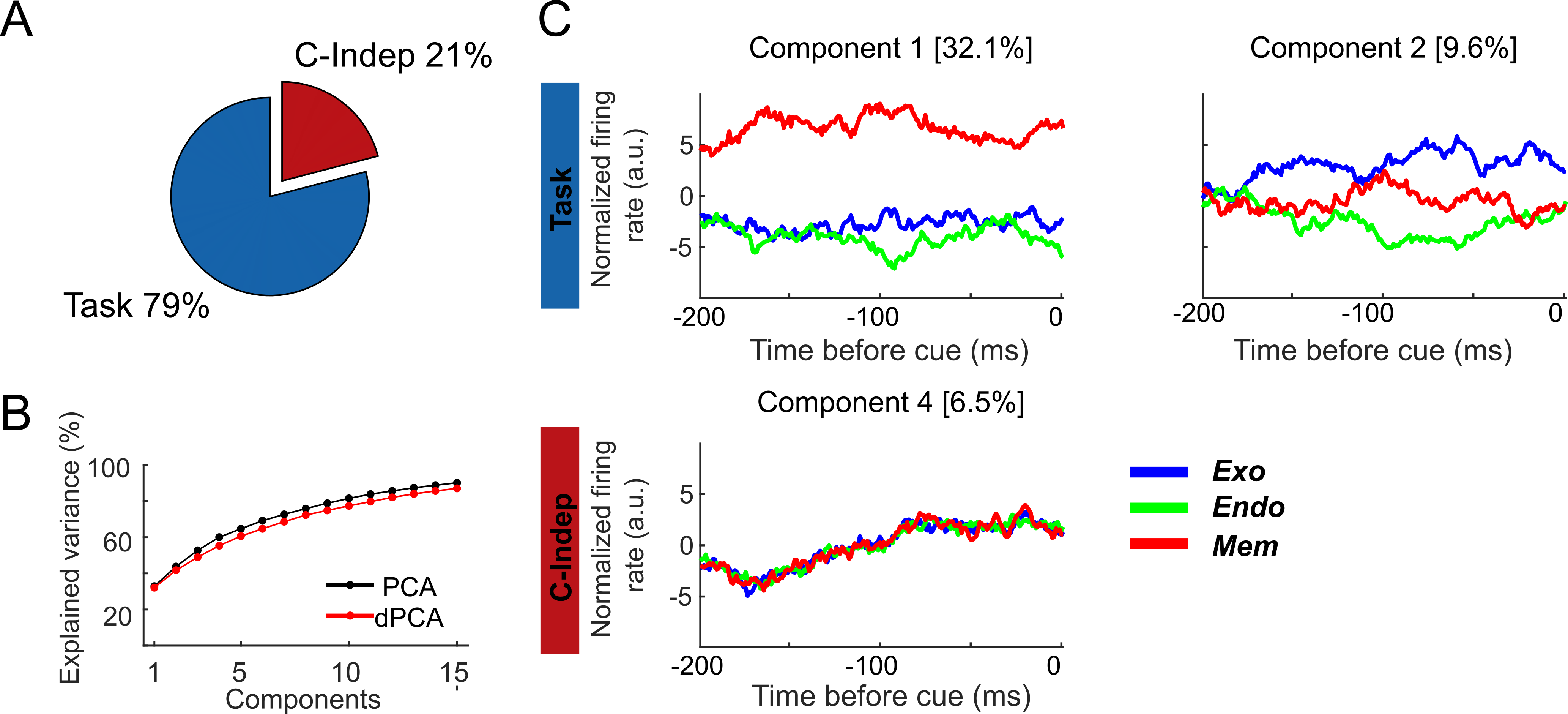

Supplement: S8 Fig — (A) Pie chart shows how the total signal variance is split among parameters: task (blue) and condition independent (red). (B) Cumulative variance explained by PCA (black) and dPCA (red). Demixed principal component show similar explained variance as PCA. (C) Demixed principal components. In each plot, normalized MUA firing rate averaged per task (in arbitrary units, exogenous (blue); endogenous (green) and memory-guided saccade (red) in the interval from −200 ms to 0 ms locked to the cue onset are projected onto the task-related dPCA components. Thick black lines show time intervals during which the task-information can be reliably extracted from single-trial activity (assessed against 95% C.I.). (D) True positive decoding rate for PC1 task-related. (E) True positive decoding rate for PC2 task-related. Dashed lines in (C) and (D) represent the 95% C.I. Note that the decoding presented in Fig 4 could not be reproduced here, due to the fact that SUA data was not extracted for all recording sessions. Data and code to S8 Fig can be found at https://osf.io/z8eh9/. (TIFF) [file pbio.3003353.s010.tiff]

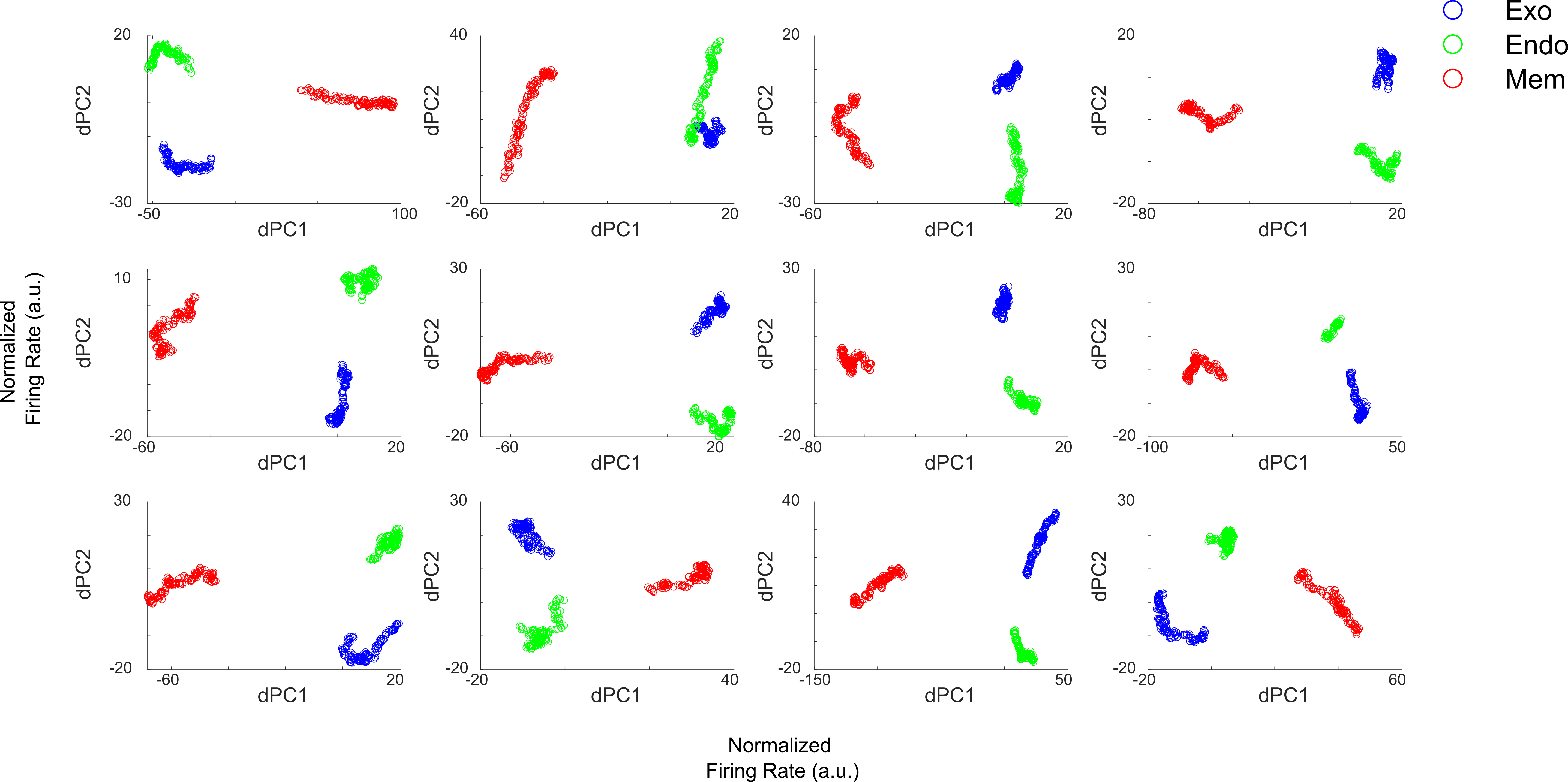

Supplement: S9 Fig — Each point corresponds to the projection of the activity in each trial (averaged in the time interval −200 to 0 ms pre-Cue, each circle thus corresponds to one trial) in each task (Exogenous task, blue; Endogenous task; green; Memory saccade task, red). Each plot corresponds to a different session. Plots 1–7 are from monkey 1 and plots 8–12 are from monkey 2. Data and code to S9 Fig can be found at https://osf.io/z8eh9/. (TIFF) [file pbio.3003353.s011.tiff]

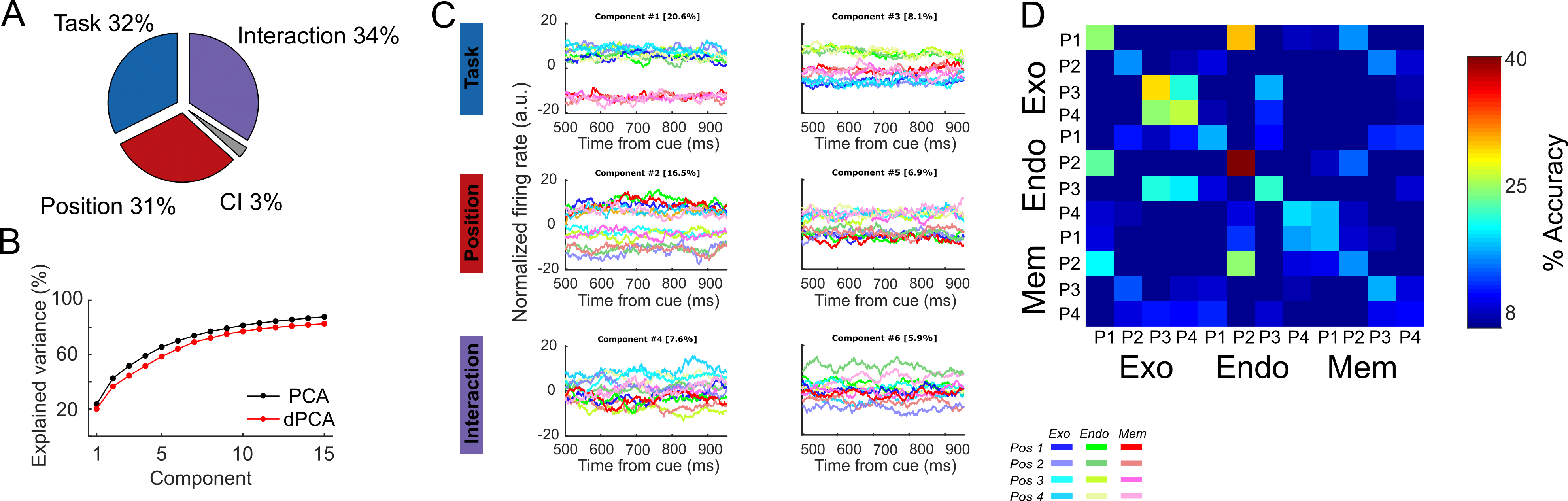

Supplement: S12 Fig — (A) Pie chart shows how the total signal variance is split among parameters: Task (blue), Position (red), Interaction (purple) and Condition independent (gray). (B) Cumulative variance explained by PCA (black) and dPCA (red). Demixed principal components show similar explained variance as PCA. (C) Demixed principal components. In each plot, MUA firing rate averaged per task (exogenous (blue shades); endogenous (green shades) and memory guided saccade (red shades)) and cued position in each task, in the interval from 500 ms to 1,000 ms locked to cue onset are projected onto the task-related, position-related and interaction dPCA components. Thick black lines show time intervals during which the parameter information can be reliably extracted from single-trial activity (assessed against 95% C.I.). (D) Confusion matrix of decoding task and position using the demixed interaction components, averaged across all sessions. Absolute chance level of the confusion matrix is 1/12 = 0.08. All elements along the diagonal are significantly above the 95% C.I. Data and code to S12 Fig can be found at https://osf.io/z8eh9/. (TIFF) [file pbio.3003353.s014.tiff]

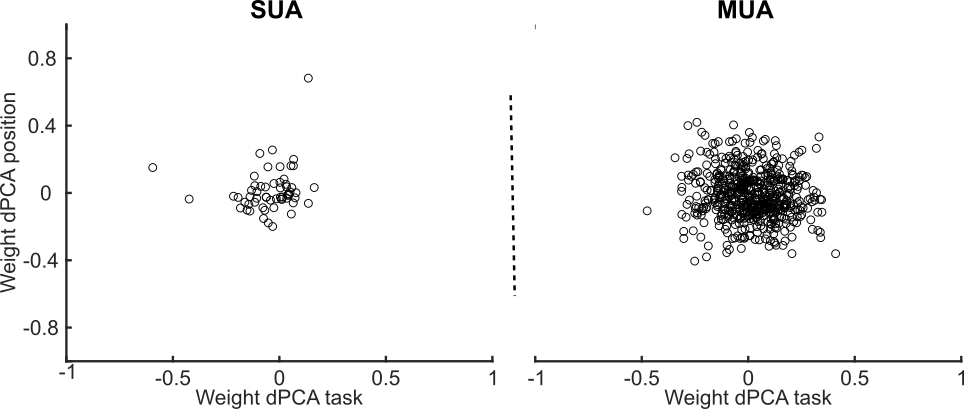

Supplement: S13 Fig — Data and code to S13 Fig can be found at https://osf.io/z8eh9/. (TIFF) [file pbio.3003353.s015.tiff]

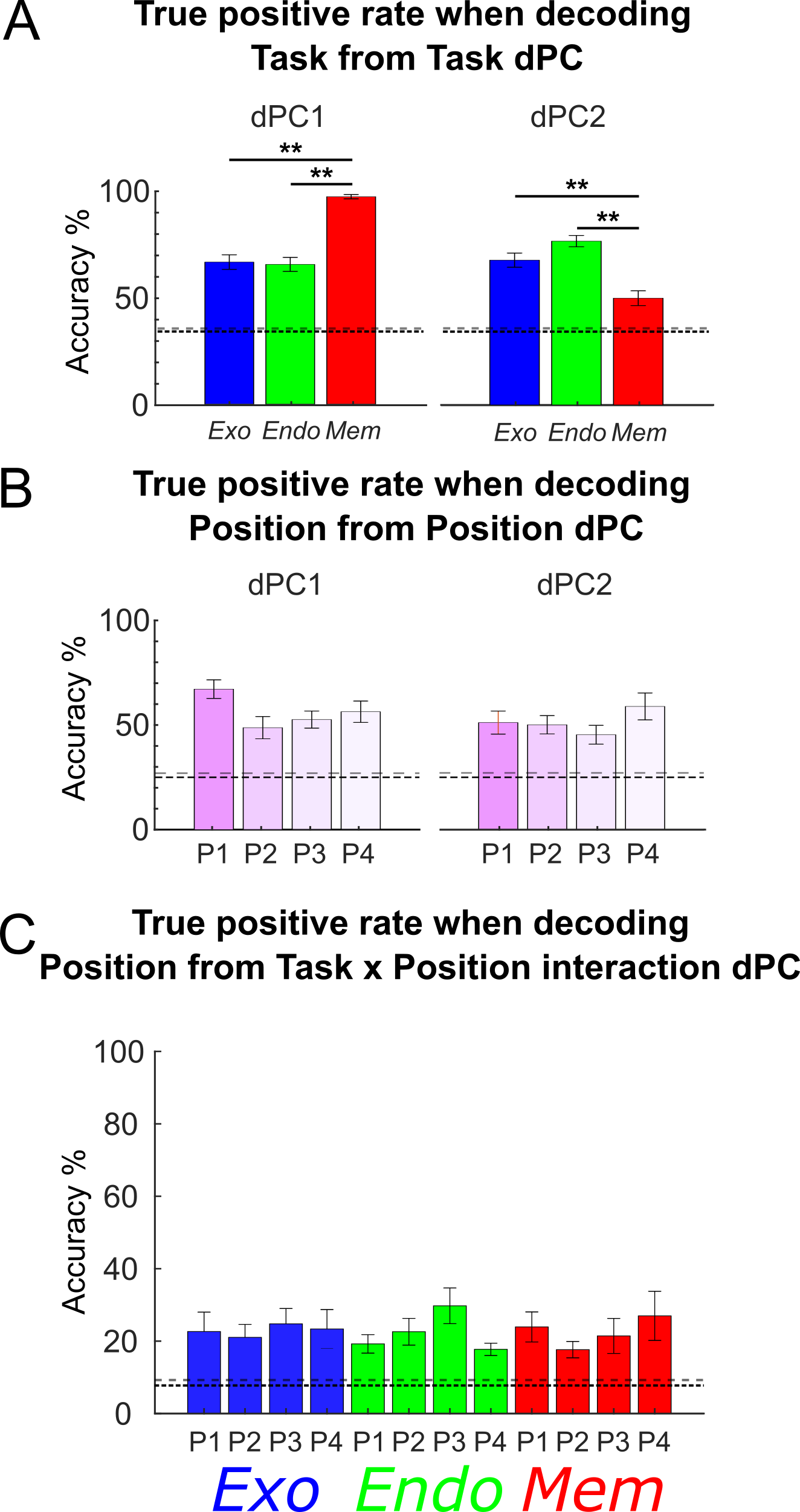

Supplement: S14 Fig — (B) Barplot corresponding to the true positive rates obtained using the first position-related demixed principal components as a linear classifier to classify trials by position (1-way ANOVA, position as main factor, on dPC1: p = 0.0367; post-hoc Bonferroni: all positions comparisons showed no significant difference, p > 0.05; 1-way ANOVA, position as main factor, on dPC2: p = 0.4449). (C) Barplot corresponding to the true positive rates obtained using the first task × position interaction demixed principal component as a linear classifier to classify trials by position in each task (2-way ANOVA, position × task; task: p = 0.7971; position: p = 0.8539; interaction: p = 0.4104). In all plots, black dashed lines indicate absolute chance level. Gray spaced dashed lines indicate 95% C.I. Data and code to S14 Fig can be found at https://osf.io/z8eh9/. (TIFF) [file pbio.3003353.s016.tiff]
