# Supplementary material for: The prefrontal cortex encodes task-identity information and flexibly adjusts its sensory processes as a function of the specific ongoing task
Source: PLoS Biol. 2025 Aug 26;23(8):e3003353. doi: 10.1371/journal.pbio.3003353 (PMC12463330; doi:10.1371/journal.pbio.3003353)
Supplement: Attachment — Submitted filename: Resp2Rev_v7.docx [file pbio.3003353.s017.docx]

**We would like to thank both reviewers and the editor for their positive assessment of our work and the constructive comments and insightful questions. We believe that this paper is now improved thanks to their feedback on our work.**

**Please find below a point-by-point response to the reviewers’ comments. Reviewers’ questions are in** black normal fonts**. Responses and changes made to original version of the manuscript are in bold red. Text from the original version of the manuscript is in** normal blue font**.**

## Reviewer #1:

In this manuscript, Mouille et al examine how neuronal activity in the Frontal Eye Field (FEF), in the prefrontal cortex of monkeys, encodes task identity and spatial information. To this end, they have employed multiunit activity recorded from the two hemispheres, during three different tasks: two cued spatial attention tasks and a memory guided saccade task. There are three main findings: a) task identity is encoded by FEF activity at the population level during fixation and before the onset of the spatial cue, b) several FEF sites encode both task identity and spatial information following the cue, some also in a non-linear manner and c) different components of population activity in the FEF can discriminate on one hand task context and on the other hand spatial information in different tasks. They suggest that this pattern of mixed selectivity results in high dimensional representations that can encode flexibly and accurately behaviorally relevant information according to current demands.

The analyses are sound and interesting and support the authors' conclusions. Conceptually, the paper provides convincing evidence and innovative findings to answer a timely question: how different types of behaviorally relevant information including task, are represented in the brain within a single cortical area. Thus, I believe the paper advances our current knowledge. I do, however, have some concerns and suggestions that can hopefully help clarify some of the methods, results and their presentation. I list these below:

**We would like to thank reviewer 1 for the positive assessment of our work.**

### Major

1)	I am confused about the summary of results provided in the concluding paragraph of the Introduction. It is written that "spatial information is represented independently from task-related information, indicating that task-identity is represented at the same hierarchical level as specific task parameters such as position". Presumably this describes the fact that in the demixed PCA analysis different components were largely found to explain the variance for task vs spatial information. However, the authors did find a component of activity that encoded the interaction of the two variables, which would indicate that task identity and spatial information are not encoded independently in absolute terms. Please, explain.

**We thank to the reviewer for this comment. As the reviewer indicates in his/her comment, when we reduced dimensionality of the recordings using dPCA, we found two components that explained variance associated to task-identity and spatial information. Since dPCA does not impose orthogonality between these components, we tested whether these two components were significantly non-orthogonal. By means of this procedure, we found that the angle was close enough to 90 degrees and therefore we conclude that these two components were statistically orthogonal. This result indicates that task identity and sensory information are not redundant or correlated with each other. However, we found a third component that explained the variance associated to the interaction between task-identity and position. This finding suggests that some neurons in the neural population might respond more strongly to specific sensory inputs during a particular task. Therefore, there are neural activity patterns that cannot be fully explained by either task-identity or sensory information alone. In summary, while the task-identity and sensory information components are orthogonal in the sense that they represent distinct dimensions of neural variability, the presence of an interaction component reflects the complex interplay between these dimensions in shaping neural responses.**

**We have clarified this paragraph of the introduction, accordingly.**

[…]

We found that the neuronal processes underlying each task were encoded in two different orthogonal components, one component reflecting the encoding of either attention versus working memory, and the other component reflecting the encoding of endogenous versus exogenous attentional enhancement. **This possibly suggests that task-identity is represented at the same hierarchical level as specific task parameters such as position. However**, we **also** identified **an additional** component that encoded the interaction between task-identity and sensory processing, indicating that these two sources of information (task and position of the sensory information) could be decoded simultaneously by a single linear readout, though the readout of sensory information varied across tasks. **This result suggests that task identity and spatial information are not encoded independently in absolute terms such that the specific state of one variable influences the specific computations implemented by the other variable.** At the fundamental level, it will be important to pursue on this work in order to better understand the specific dimensions that the prefrontal cortex uses to represent task identity **and achieve perceptual representation stability across tasks**. At the translational level, this work opens new venues for the implementation of brain computer interface technologies based on machine learning algorithms able to extract complex cognitive information from real-time PFC recordings.

2)	There are a number of points that are not clear in the Methods.

a)	In the description of the attention tasks, the authors refer to a change in luminosity of targets or distractors that the monkeys should respond to. Was that an increase or decrease in luminosity and what were the exact levels of luminance before and after the change. Although this was similar in the two attention tasks, it is critical to appreciate the difficulty of detecting the change and thus the required cognitive load in each task.

**We appreciate this comment. This information is now specified as follows:**

After the cue presentation, the monkeys needed to orient their attention to the target landmark in order to monitor it for **an increase** in luminosity while maintaining eye fixation onto the central blue cross. This **increase** **in** luminosity (from now on, the target) could occur anywhere between 500 to 2800 ms from cue onset. **The increase in luminosity was titrated during preliminary behavioral experiments, independently for each of the four possible target positions, such that behavioral performance during the Exo task would be in the range of 85-90% at each location.**

[…] The endogenous task was identical to the exogenous task in all aspects**, including parameters of target luminosity increase,** except for the location and size of the cue (Fig. 1B, panel 1).

b)	The data preprocessing for multi-unit activity is somewhat confusing. It is mentioned that "epoched MUA data were baseline corrected (independently for each channel and each trial) relative to the period from -500 ms to -300 ms before the cue". Does this refer to the firing rate plots shown in Figure 2A,B? This doesn't seem to be the case, yet it is unclear what kind of normalization was done in these plots. I am not sure why correction was applied in each trial rather than the average across trials. If on the other hand, this refers to preprocessing for the PCA or dPCA analysis this should be clearly stated and a description of normalization for data shown in plots Fig 2A,b should be added.

**We apologize for lack of clarity. Indeed, this refers to preprocessing for the PCA or dPCA analysis and figure 2ab involves a different normalization. This is now specified in the text as follows:**

[…] In each task, MUA activity was epoched per trial. Spike trains were filtered with a Gaussian kernel (δ = 60 ms). First, MUA activity previous to the cue onset was epoched from -500 ms to 0 with respect to the onset of the Cue for the Pre-Cue analysis. Then, MUA activity was epoched between 500 ms to 0 ms prior to Target onset for the Delay analysis. To extract the averaged firing rates **for** **dPCA analysis**, these epoched MUA data were baseline corrected (independently for each channel and each trial) relative to the time period from -500 ms to -300 ms before the cue. **This reduced trial-to-trial variability in baseline activity levels.**

**Figure 2. Mixed selectivity corresponds to the ability of a neuronal population to encode simultaneously independent sources of information:** (A) Firing rate of the same MUA in the three tasks aligned on the cue (200 ms before the cue onset to 1000 ms after the cue onset) in exogenous task (blue), endogenous task (green) and memory guided saccade task (red) for preferred (**solid** line) and non-preferred (dash line) position. **The data was z-scored relative to mean and standard deviation of average individual trial responses during baseline (-150 to 0 ms before cue onset).** **Gray box corresponds to the delay period during which neuronal activities were analyzed for subplots C and D.** (B) Mean firing rate of the same neuronal population in the three tasks aligned on cue presentation (200 ms before the cue onset to 1000 ms after the cue onset) in the exogenous task (blue), the endogenous task (green) and the memory guided saccade task (red) for preferred (**solid** line) and non-preferred (dash line) position. **The data was z-scored relative to mean and standard deviation of average individual channel responses during baseline (-150 to 0 ms before cue onset).** **Gray box corresponds to the delay period during which neuronal activities were analyzed for subplots C and D.** (C) Venn diagram representing the proportion of cells showing a spatial tuning in each of the three tasks. (D) Pie chart corresponding to the proportion of neurons tuned to only position (red), task (blue) and both (mixed-selectivity; yellow). Non-selective neurons are plotted in grey. **Wilcoxon ranksum test was performed between baseline and time interval for each channel and position across trials. A non-parametric 2-Way-ANOVA was performed on selective channels with Bonferroni_Holm correction on spatial and task factor (p<0.01).**

**Supplementary Figure 1.** (A) Firing rate of **a representative** MUA (same as in Figure 1) aligned to target onset (400 ms before target onset to 600 ms after target onset) averaged across trials of the exogenous attention task (blue), the endogenous attention task (green) and the memory guided saccade task (red) for preferred (full line) and least-preferred (dash line) positions. **The data was z-scored relative to mean and standard deviation of average individual trial responses during baseline (-150 to 0 ms before cue onset).** (B) Mean firing rate across the neuronal population (MUA) aligned on the target (-400 ms before target onset to 600 ms after target onset) as a function of tasks and positions (same as in A). **The data was z-scored relative to mean and standard deviation of average individual channel responses during baseline (-150 to 0 ms before cue onset).**

c)	To assess spatial selectivity the authors used a Wilcoxon test comparing activity in the pre-cue and post-cue period. Was this a signed rank test? What was the p-value used to classify a channel as selective? Was activity for each channel averaged across all four cued positions? I do not understand how this comparison examines spatial selectivity. Comparison with baseline does not compare across positions and may have over- or underestimated spatial selectivity by including visually responsive channels rather than spatially selective channels. An ANOVA or a Kruskal Wallis across all four positions in the post cue period would be more appropriate.

**We apologize for lack of clarity. Below is the updated and corrected version of the statistical procedure to characterize spatially selective channels:**

Spatially selective channels **during the delay epoch** were identified **as follows. First,** a non-parametric Wilcoxon test **was used to identify responsive channels, i.e. channels for which the activities observed during the baseline and during the post cue period (800 to 900 ms following cue onset, only considering trials for which target or fixation off event happened 1000ms after cue presentation) were significantly different for at least one of the four possible spatial cued positions (p<0.05).** For each responsive channel, a **non-parametric** two-way ANOVA Task x Position **(David Stern,** [**https://www.mathworks.com/matlabcentral/fileexchange/44308-randanova2**](https://www.mathworks.com/matlabcentral/fileexchange/44308-randanova2)**, this method uses the Still and White's (1981) approach to calculate P-value for interaction effect)** was then implemented on the average activity in the **post cue period i**n order to identify the Task related channels, the spatially selective channels as well as the mixed selectivity channels (**p<0.01**). All p values were corrected for multiple comparisons using a Holm-Bonferroni correction.

3)	In the Results, in the first paragraph of page 8, it is stated that "Although these three tasks showed some similarities in their structure, they recruited different cognitive processes". However, differences are not limited to cognitive demands. The sensory conditions are also very different and this should be clearly stated. Particularly, when comparing the memory guided saccade task to the attention tasks, the absence of stimuli in the former imposes a critical difference that could in principle explain the results beyond cognitive demands. It is the fact that differences across the two attention tasks that are more similar in structure are found that allows the authors to generalize their conclusions about task identity encoding. I believe this should be discussed.

**We have now clarified this point as follows:**

Although these three tasks showed some similarities in their structure, they recruited different cognitive processes. While monkeys needed to enhance visual attention in the cued quadrant to respond to the target onset in the exogenous and endogenous attentional tasks (Amengual et al., 2022; Astrand et al., 2020, 2016), they needed to memorize the location of the cue to produce an oriented eye movement towards that location. **Sensory conditions were also different. Indeed, in the attentional tasks, peripheral landmarks were present all throughout the trial, while they were absent in the memory guided saccade task. While this probably did not impact pre-cue neuronal activity, it possibly impacted post-cue task differences observed in the activity.**

4)	In the last paragraph of page 8 it is stated that both monkeys showed lower false alarm rates in the endogenous task compared to the exogenous task, but the opposite is shown in Figure 1B.

**We thank to the reviewer for this comment. We apologize for this confusion. This is now corrected as follows:**

Both monkeys showed lower false alarm rates in **Exo** than in **Endo** (fig.1.B M1: 7% vs. 13%, Wilcoxon ranksum test, M1: Z=2.56, p=0.007; M2: 10% vs. 21%, Wilcoxon ranksum test, Z=0.63, p=0.0079).

5)	On page 10, the authors report the proportion of channels that were found to be selective for position only, task only and both task and position. I am not sure how to interpret these numbers. Given the variability in firing rates, even across trials, I can think of potential confounding factors that would need to be ruled out. For example, is it possible that task-related selectivity is a result of changes in the signal over time given that the tasks were run in blocks? How do these numbers e.g. for position selectivity or mixed selectivity compare to those expected by chance (by shuffling trials)?

**We appreciate this comment. As the reviewer suggests,** **the changes might be accounted for by changes in the signal along the recording session, resulting in possibly different firing rate fluctuations not related with the cognitive aspects of the tasks. This is addressed in supplementary material M1 as follows. See also our response to reviewer 2, major point 3.**

**Supplementary material M1:** **Stability of MUA selectivity across trials. Task-related selectivity might be accounted for by changes in the signal along the recording session, resulting in possibly different firing rate fluctuations not related with the cognitive aspects of the tasks. To reduce this effect, we decided to work with normalized activity with respect to the baseline in each trial. Furthermore, to prove that our selectivity results are not driven by systematic firing rate fluctuations driven by the time in the session, we conducted a supplementary analysis in which we extracted the task and position selectivity by selecting trials randomly along the recording session. In this way, our data did not contain any temporal structure given by the time-course along the recording session, nor information about task or position. We performed this procedure 100 times and computed the % of position, task, mixed selectivity and non-selective channels. The 95% confidence interval for both task and position are below 2.5% for all categories. We conducted a second analysis in which we computed the % of position, task, mixed selectivity and non-selective channels over a randomly selected subset of 10 trials for each possible trial category. We repeated this procedure 100 times. The outcome of this analysis is very close to that reported in main** **figure 2. These supplementary analyses are captured by the figure below. Over all, these additional analyses indicate that the reported results in main figure 2 are robust to neuronal variability as well as to possible temporal structure in the task.**

**Barplot corresponding to the proportion of MUAs tuned to only position (red), task (blue) and both (mixed-selectivity; yellow, mean +/- s.e. computed over 100 repetitions of over a random selection of 10 trials for each possible trial category). Non-selective MUAs are plotted in grey. Black dashed line corresponds to the 95% CI computed independently for each category of cells. Note that the average proportion of non-selective MUAs is very low relative to the 95% CI estimated from a random permutation procedure.**

**Supplementary material M1 is now called in the main text as follows: This categorization was robust against possible sources of noise in the data (supplementary material M1) …**

6)	On page 12, second paragraph, a 2-way ANOVA is mentioned but the two factors are not mentioned and neither are the p-values for the two factors. Only one p-value is reported. The same is true in several other places in the text (e.g. three lines below, legend of supplementary figure 4)

**We apologize for the oversight. This should have been a 1-way ANOVA with task as main factor. This is now corrected as follows:**

Figure 3D shows that for the first dPC, the TPR in classifying the memory task was 80% (iqr 7.3), while the TPR for both attentional tasks were significantly lower and not significantly different one from the other (**1**-way ANOVA, **task as main factor**: p = 1.1835e-15, Exo vs Mem: post-hoc Bonferroni test: p = 5.4277e-14; Endo vs Mem: post-hoc Bonferroni test: p = 1.8701e-14; Exo vs Endo: post-hoc Bonferroni p>0.05) suggesting a dissociation between attentional and working memory processes. In contrast, TPR in predicting the memory task using the dPC2 was lower than predicting both exogenous and endogenous attention (**1**-way ANOVA, **task as main factor**: p = 0.002, Exo vs Mem: post-hoc Bonferroni: p = 0.0218; Endo vs Mem: post-hoc Bonferroni: p = 0.0023; Exo vs Endo: post-hoc Bonferroni: p>0.05) suggesting a dissociation in the way the attentional processes (exogenous vs endogenous) were implemented.

**(Supplementary Figure 4 is now supplementary Figure 6)**

Supplementary Figure **6**. (A) Barplot corresponding to the true positive rates obtained using the first task-related demixed principal components as a linear classifier to classify trials by task (Endogenous attention, blue; Exogenous attention, green; Memory saccade, red) (**1**-way ANOVA, **task as main factor, on** dPC1: p = 9.6184e-10: Exo vs Endo: post-hoc Bonferroni: p>0.05; Exo vs Mem: post-hoc Bonferroni: p = 1.8546e-8; Endo vs Mem: post-hoc Bonferroni: p = 8.6775e-9; **1**-way ANOVA, **task as main factor, on** dPC2: p = 4.0943e-6, Exo vs Endo: post-hoc Bonferroni: p>0.05; Exo vs Mem: post-hoc Bonferroni: p = 0.0011; Endo vs Mem: post-hoc Bonferroni: p = 3.1361e-6). (B) Barplot corresponding to the true positive rates obtained using the first position-related demixed principal components as a linear classifier to classify trials by position (**1**-way ANOVA, **position as main factor, on** dPC1: p = 0.0367; post-hoc Bonferroni: all positions comparisons showed no significant difference, p>0.05; **1**-way ANOVA, **position as main factor, on** dPC2: p = 0.4449). (C) Barplot corresponding to the true positive rates obtained using the first task x position interaction demixed principal component as a linear classifier to classify trials by position in each task (**2**-way ANOVA, **position x task;** **task: p = 0.7971; position: p = 0.8539; interaction: p = 0.4104**). In all plots, black dashed lines indicate absolute chance level. **Gray spaced dashed lines indicate 95% C.I.**

7)	The authors used the interaction component (position x task) to decode position and task information. Although accuracies are significantly above chance, they are very low. What are the implications of this finding for the authors' conclusions? How do such low accuracies fit into the context of high dimensional representations subserved by mixed selectivity? A more detailed discussion is needed.

**We appreciate this comment from the reviewer. We added the following paragraph to the discussion:**

[…] All in all, our results reinforce previous studies suggesting that the dynamics of the encoding of sensory information might be different based on the underlying cognitive process recruited by the task. **In addition, we found that the interaction between task and position explained 20% of the variance. This indicates a strong influence of the high-dimensional representation of sensory information on population coding. We decided to focus on the first interaction dPC (accounting for 6.2% of total variance and 32.2% of variance assigned to the interaction) to check classification accuracies. These were above the absolute chance level (8.33%) as well as above the 95% C.I. Yet they can still be considered as low. An important question is thus whether these interaction components have a functional and/or behavioral impact or not. In a previous study, we show that the degree of interaction impacts overt behavioral performance (Amengual et al., 2022). How the interaction component reflecting different coding of position across tasks we describe in the present work reflects on behavior is yet unknow. One might for example predict a correlation between the strength of this interaction term and perceptual sensitivity or response criterion (and hence speed) in each task. This will require further investigations in a different set of experiments.**

8)	In the discussion (first paragraph) the authors write: "the dynamics of the activity of the FEF cells when their preferred position was cued was different across tasks…". Why not also when their least preferred position was cued? None of the analyses was specific for the cued position, was it? This is rather confusing.

**This is actually correct (as can be seen in figure 2a and 2b). We have now rephrased as follows:** We found that, at the neural level, the dynamics of the activity of the FEF cells ~~when their preferred position was cued~~ was different across tasks, suggesting a different encoding of the sensory information as a function of the task being performed.

9)	In the legend of Supplementary Figure 2 when referring to "Projection of MUA" it should be clarified that this is population activity

**This is now clarified as follows: (now Figure 4)**

**Supplementary Figure 4.** Projection of MUA **population activity** onto the first and second principal components extracted with a PCA analysis. Each point corresponds to the projection of the activity in each trial (averaged in the time interval -300 to - 100ms pre Cue, each circle thus corresponds to one trial) in each task (Exogenous task, blue; Endogenous task; green; Memory saccade task, red). Each plot corresponds to a different session. Plots 1 to 7 are from monkey 1 and plots 8 to 12 are from monkey 2.

10)	In the legend of Supplementary Figure 4, in B and C there are no statistics for the comparison to chance

**We have now implemented the statistics relative to chance. The new figure and its legend are updated below:**

Supplementary Figure 4. (A) Barplot corresponding to the true positive rates obtained using the first task-related demixed principal components as a linear classifier to classify trials by task (Endogenous attention, blue; Exogenous attention, green; Memory saccade, red) (**1**-way ANOVA, **task as main factor, on** dPC1: p = 9.6184e-10: Exo vs Endo: post-hoc Bonferroni: p>0.05; Exo vs Mem: post-hoc Bonferroni: p = 1.8546e-8; Endo vs Mem: post-hoc Bonferroni: p = 8.6775e-9; **1**-way ANOVA, **task as main factor, on** dPC2: p = 4.0943e-6, Exo vs Endo: post-hoc Bonferroni: p>0.05; Exo vs Mem: post-hoc Bonferroni: p = 0.0011; Endo vs Mem: post-hoc Bonferroni: p = 3.1361e-6). (B) Barplot corresponding to the true positive rates obtained using the first position-related demixed principal components as a linear classifier to classify trials by position (**1**-way ANOVA, **position as main factor, on** dPC1: p = 0.0367; post-hoc Bonferroni: all positions comparisons showed no significant difference, p>0.05; **1**-way ANOVA, **position as main factor, on** dPC2: p = 0.4449). (C) Barplot corresponding to the true positive rates obtained using the first task x position interaction demixed principal component as a linear classifier to classify trials by position in each task (**2**-way ANOVA, **position x task;** **task: p = 0.7971; position: p = 0.8539; interaction: p = 0.4104**). In all plots, black dashed lines indicate absolute chance level. **Gray spaced dashed lines indicate 95% C.I.**

### Minor

1)	In Figure 1 where the tasks are shown, the last part of the memory guided saccade task, where according to the description in the methods, the monkey waited for the peripheral stimulus to change color from red to grey is not shown

**Figure 1 has been changed to implement this comment. Updated figure 1 is appended below. The legend does not require adjustments.**

2)	Page 8 first line: replace "avoid" by "ignore"

**This was implemented as follows:**

In both endogenous and exogenous attention tasks, distractor stimuli were presented during the cue to target interval (CTOA), and monkeys needed to **ignore** them (otherwise, the trial was cancelled).

3)	Page 8, penultimate paragraph, last sentence: "in despite" delete "in"

**This was implemented as follows:**

Regarding the attentional tasks, **~~in~~** despite of no significant difference, RTs tended to be slower for the Endo than for the Exo task as reported in other studies (Astrand et al., 2016).

4)	There are a few instances where the authors do not consistently use either past or present tense in the same sentence. For example, on page 10, first line: "…these recorded neurons SHOW enhanced responses when the cue WAS presented…". Similarly on page 15, 5 lines before the end: "These results ARE in line with prior studies (Bahmani et al., 2019; Panichello and Buschman, 2021), and SHOWED…"

**This was implemented as follows:**

As reported in previous studies (Amengual et al., 2022; Di Bello et al., 2021; Ibos et al., 2013; Moore and Armstrong, 2003), these recorded neurons **showed** enhanced responses when the cue was presented in or was cueing towards their preferred spatial position, relative to when it was presented in the least preferred spatial location, **and this was true for all three tasks** (Figure 2A&B).

The projection of the firing rates onto the second dPCA associated to the task show a dissociation

between the exogenous (blue) and endogenous (green)**~~s~~** tasks suggesting a dissociation as a function of the attentional task-demands specifically within the attentional tasks.

We found that the FEF **encoded** task identity in two sources of task-related variability (independently from the processing of sensory information) that **segregated** in orthogonal components that **conformed** a two-dimensional space, with one component dissociating spatial working memory vs spatial attention processes, and the other component dissociating specifically **the two** spatial attentional processes (endogenous **vs exogenous**). Additionally, we observed that the neuronal population **encoded** task-identity and trial-relevant spatial information in a unique interaction component that can be decoded using a single linear readout.

Prior evidence showed that FEF **encoded** spatial information during the performance of attentional tasks (Amengual et al., 2022; Armstrong et al., 2009; Bruce and Goldberg, 1985; Di Bello et al., 2021; Goldberg and Segraves, 1987; Paneri and Gregoriou, 2017) and working memory tasks (Baddeley, 2012; Bahmani et al., 2019; Funahashi and Kubota, 1994; Noudoost et al., 2021, 2021; Sapountzis et al., 2022).

These results are in line with prior studies (Bahmani et al., 2019; Panichello and Buschman, 2021), and **show** the ability of this area to modulate its patterns of neuronal responses as a function of underlying cognitive process.

5)	In the same sentence rephrase "…and this for all three tasks" by e.g. and this was true for all three task

**This was implemented as follows:**

As reported in previous studies (Amengual et al., 2022; Di Bello et al., 2021; Ibos et al., 2013; Moore and Armstrong, 2003), these recorded neurons **showed** enhanced responses when the cue was presented in or was cueing towards their preferred spatial position, relative to when it was presented in the least preferred spatial location, **and this was true for all three tasks** (Figure 2A&B).

6)	The title in Figure 2 should change to be more specific and reflect the content

**This was implemented as follows:**

**Figure 2. Mixed selectivity corresponds to the ability of a neuronal population to encode simultaneously independent sources of information.**

7)	Page 10, line 4: "24% of cells were tuned to the three different tasks". Do the authors mean in all three tasks

**We adapted the interval of selectivity for this analysis to the time interval of the analysis performed for Figure 2D (from 500 to 1000ms after the cue onset). As a result, the result of the analysis of the Figure 2C changed. This was implemented as follows:** 17% of cells were tuned **to position** **in all three tasks**

8)	In the same sentence: "suggesting that there is a neural population encoding multiple cognitive processes (spatial attention & spatial working memory)". Please rephrase.

**This was implemented as follows:** 17% of cells were tuned **to position** **in all three tasks** **indicating that these cells encoded spatial information both during spatial attention and spatial working memory.**

9)	On page 11, first paragraph: "Dimensionality reduction consists of projecting…" use either consist in or rephrase

**This was implemented as follows:** Dimensionality reduction consists **in** projecting the high-dimensional neuronal recordings onto a low-dimensional space minimizing the information loss.

10)	Page 12, line 5: replace show by shows

**This was implemented as follows:** The projection of the firing rates onto the second dPCA associated to the task **shows** a dissociation between the exogenous (blue) and endogenous (green)s tasks suggesting a dissociation as a function of the attentional task-demands specifically within the attentional tasks.

11)	Page 12, four lines before the end: "using the dPC" do the authors mean using the second dPC?

**Indeed. This was implemented as follows:** In contrast, TPR in predicting the memory task using the **second** dPC was lower than predicting both exogenous and endogenous attention attention (**1**-way ANOVA, **task as main factor**: p = 0.002, Exo vs Mem: post-hoc Bonferroni: p = 0.0218; Endo vs Mem: post-hoc Bonferroni: p = 0.0023; Exo vs Endo: post-hoc Bonferroni: p>0.05) suggesting a dissociation in the way the attentional processes (exogenous vs endogenous) were implemented.

12)	Page 13, line 6: "we will focused" use either we focused or we will focus

**This was implemented as follows:** Here, we were interested in the task and target spatial information, thus, we will **focus** on the delay period between the cue and the target in order to include spatial information, as opposed to the pre-cue interval that had been selected in our previous analysis in which there was no spatial information available yet.

13)	Legend of Figure 4: "per tasks" replace by per task

**This was implemented as follows:** In each plot, MUA firing rate averaged **per task** (exogenous (blue shades); endogenous (green shades) and memory guided saccade (red shades)) and cued position in each task, in the interval from **500 ms to 1000 ms locked to cue** onset are projected onto the task-related, position related and interaction dPCA components.

14)	Page 14, four lines before the end: "dissociating specifically both spatial attentional processes" replace both by the two.

**This was implemented as follows:** We found that the FEF **encoded** task identity in two sources of task-related variability (independently from the processing of sensory information) that **segregated** in orthogonal components that **conformed** a two-dimensional space, with one component dissociating spatial working memory vs spatial attention processes, and the other component dissociating specifically **the two** spatial attentional processes (endogenous vs exogenous).

15)	Page 16, first paragraph: "These results extended…" replace extended by extend

**This was implemented as follows:** These results **extend** those reported by Astrand and colleagues (Astrand et al., 2015), where they showed a different population dynamics and code stability as a function of the source of the information and task.

## Reviewer #2:

This is a neurophysiological study in macaque monkeys that compared 3 different tasks (memory-guided saccade task and two spatial attention / detection tasks with manual responses) in the same recording sessions, in order to answer the question whether there is task identity representation besides the representation of task-relevant parameters such as spatial position selectivity, in the frontal eye fields (FEF). I have read it with great interest. The results show that indeed, the task identity is represented in FEF and the two attentional tasks are represented similarly to each other but differently from the memory-guided saccade task. Furthermore, there is an interaction between spatial selectivity and the task-identity selectivity after the spatial information is available, suggesting a high-dimensional representation of spatial factors that might facilitate flexible adjustment to specific task demands.

These results are of substantial interest and, as far as I can judge, are novel for the FEF and might constitute a significant advance to warrant a publication in PLoS Biology. I have however a number of concerns or questions related to insufficient methodological descriptions, experimental design, and presentation of the results, as well as suggestions for additional analyses, which should be addressed in a revision.

### Major:

1) Page 6 : The electrophysiological methods are not sufficiently clear and need to be explained better and more comprehensively:

a) "A threshold defining the MUA was applied independently for each recording contact and before the actual task-related recordings started." - this is not clear, please describe the procedure in more detail and explain why the threshold has been defined online and not during the data analysis stage offline, taking into account recording SNR etc. Please explain how the MUA signal was derived (e.g. as envelope of the MUA energy, or the actual threshold crossings resulting in "multiunit spike trains" - as it seems to be the case) and what were the thresholds relative to some measure of the signal SNR - e.g. in terms of number of standard deviations of the activity.

**This point was clarified as follows:** A threshold was applied independently for each recording contact and before the actual task-related recordings started**, such that the crossing of this threshold resulted in multiunit spike trains. This threshold corresponded to 3 standard deviations from mean activity during unconstrained eye movements by the monkeys while exploring a blank screen. This threshold was confirmed during the offline analyses.**

b) Furthermore, the exclusive emphasis on multiunit activity is surprising, especially having in mind the putative mixed selectivity properties. It seems that for the aims of the current study, carefully spike-sorted single neurons would be better, at least when the signal quality would allow reliable spike sorting. It seems that there was no attempt to pursue proper spike sorting. Please explain/justify the choice of this approach. I would highly recommend to supplement the results of MUA with single neuron analysis.

**We fully appreciate this comment by reviewer 2. Ideally, spike sorting would indeed provide a more accurate description of mixed selectivity in the FEF in relation with the different variables of interest. However, the focus of the study was to describe how neuronal FEF population encodes information, rather than understand the precise mechanisms of mixed selectivity and the contribution of specific individual neurons to neuronal computations. In this respect, a recent study published by Trautman and colleagues (2019) shows that dimensionality reduction methods applied to MUA activity provide homologous results to when these methods are applied to the underlying SUA activity. The following paragraph is introduced in the demixed PCA analysis methods section:**

**A recent publication indicates that dimensionality reduction methods are accurate in the absence of spike sorting (Trautmann et al., 2019). In other words, neural dynamics and scientific conclusions are similar using multiunit threshold crossing or sorted neurons** **when using dimensionality reduction methods. This particularly applies to the present study. Indeed, 10 to 15 dimensions capture over 90% of the variability of trial-averaged neural population activity when monkeys perform the different tasks. This is associated with low error in manifold estimation (Johnson and Lindenstrauss, 1984). As a result, due to the theory of random projections, neural population dynamics can be accurately estimated from our recordings using the MUA instead of the SUA, since the former is a low-dimensional representation of the latter. In the present work, the dPCA is applied on MUA activities.**

**Trautmann EM, Stavisky SD, Lahiri S, Ames KC, Kaufman MT, O'Shea DJ, Vyas S, Sun X, Ryu SI, Ganguli S, Shenoy KV. Accurate Estimation of Neural Population Dynamics without Spike Sorting. Neuron, 2019, 103(2):292-308.e4. doi: 10.1016/j.neuron.2019.05.003.**

**Johnson W.B., Lindenstrauss J. Extensions of Lipschitz mappings into a Hilbert space. Contemp. Math. 1984; 26: 189-206.**

2) Please present number of sessions and the overall number of recording sites/channels for each animal and task, as well as the number of trials for each task in each session.

**This is now described in the methods section as follows:** **We used two probes (one in each recording site) containing 24 contacts each, in the two monkeys. Probes were placed in each recording session in a different location of the chamber (number of sessions: M1, n=7; M2, n=5**). **The median number of trials by task were as follows: Exogenous task, M1: 318 (iqr = 59); M2: 380 (iqr = 62); Endogenous task, M1: 357 (iqr = 67); M2: 589 (iqr = 171); Memory guided saccade task, M1: 244 (iqr = 39); M2: 302 (iqr= 184).**

3) Please describe how the stability of the recording was assessed, and what measures were taken to ensure that the differences observed between task blocks are not attributable to recording instability or drift, which is often observed with acute U-probe recordings.

**We appreciate this comment. This is a fundamental question. The main text was modified as follows.**

**In the section ‘Neuronal recordings’:**

Simultaneous recordings in the two FEFs were carried out using two 24-contact Plexon U-probes. The contacts had an interspacing distance of 250 μm. Neural data was acquired with PlexonOmniplex® neuronal data acquisition system (Omniplex). The data was amplified 400 times and digitized at 40,000 Hz. The neuronal data was low-cut filtered at 300 Hz. All analyses were performed on the MUA recorded from each of the 48 recording contacts. A threshold was applied independently for each recording contact and before the actual task-related recordings started, **such that the crossing of this threshold resulted in multiunit spike trains. This threshold corresponded to 3 standard deviations from mean activity during unconstrained eye movements by the monkeys while exploring a blank screen. This threshold was confirmed during the offline analyses.**

**In the section supplementary material M2:**

**Supplementary material M2:** **Despite the precautions taken to stabilize the electrodes after their insertion in the brain, and trial-based baseline correction, a drift related to physiological parameters, could still** **impact neuronal recordings along the session. In contrast with other methods such as decoding or PCA, the analysis of neuronal variability that we implemented in this work relies on demixed principal component analysis (dPCA, see below). This method has been shown in the literature not to be affected by the drift in the signal (Kobak et al., 2016). Indeed, it extracts the neuronal variance associated to specific parameters (here, task or position), and separates this neuronal variance from the parameter-independent variance.** **Monkeys performed each task for 20 minutes or more. Thus, drift could happen both within any task and between tasks. These changes in neuronal responses were captured in the parameter-independent variance.**

**We further validate that drift did not account for the main results presented in Figures 3 and 4, as follows. We performed task-dPCA on each of the independent sessions. For each session and each task, we projected the population activity on the first task-component. We then plotted the average of this activity not as a function of task, but as a function of task order in the recording session. While the memory guided saccade task was always the last task in the session, half of the sessions started with the Exo task and the other half started with the Endo task. We found that there was no statistically significant difference between the distributions of average projected activity recorded during the first and second task onto the first dPC (figure below).**

**Normalized firing rate of projected population activity on the first task-component of the dPCA per session, computed independently for the first task in the session (50% Exo, 50% Endo), the second task in the session (50% Exo, 50% Endo) and the last task in the session (100% memory-guided saccade task) (1-way non-parametric ANOVA: p = 5.0396e-14; post-hoc Bonferroni test, task 1 vs. task 2: p = 1, task 1 vs. task 3: p = 6.2112e-13, task 2 vs. task 3: p = 2.2593e-12).**

**Supplementary material M2 is called in the main text as follows:**

**In order to confirm that dPC1 reflected a genuine task-identity coding, rather than a time in the session, we performed task-dPCA on the independent sessions. For each session and each task, we projected the population activity onto the first task component. We then plotted the average of this activity not as a function of task, but as a function of task order in the session. While the memory guided saccade task was always the last task, half of the sessions started with the Exo task and the other half started with the Endo task.** **There was no statistically significant difference between the distributions of average activity recorded during the first and second task projected onto the first dPC (supplementary material M2).**

4) "Spatially selective channels were identified using a non-parametric Wilcoxon test of the activity between the baseline and a post cue period (1300 to 1400 ms following cue onset)." - Perhaps I am missing something obvious, but I don't understand what is especially relevant for the spatial selectivity in this short 100 ms interval long after the cue onset. Is this a proxy for the delay period activity? But this would not work for shorter delay periods…

**This reviewer is absolutely correct. This is a proxy for the delay period activity, far enough into the delay epoch from the visual cue-related response. In the analysis, we removed the trials in which the target was presented earlier than 1400ms after the cue. We chose an interval of 100ms to match the 100ms pre-cue interval that was used for baselining the signal. As expected from the neurophysiology of the FEF, spatial selectivity did not change substantially for a larger time interval spanning 500 to 1000ms post-cue. We however decided to follow the comment of this reviewer and we now report selectivity in this larger window running from 500 to 1000ms post-cue.**

**This is now specified in the methods section as follows:**

Spatially selective channels **during the delay epoch** were identified **as follows. First,** a non-parametric Wilcoxon test **was used to identify responsive channels, i.e. channels for which the activities observed during the baseline and during the post cue period (800 to 900 ms following cue onset, only considering trials for which target or fixation off event happened 1000ms after cue presentation) w**ere **significantly different for at least one of the four possible spatial cued positions (p<0.05).** For each responsive channel, a **non-parametric** two-way ANOVA Task x Position **(David Stern,** [**https://www.mathworks.com/matlabcentral/fileexchange/44308-randanova2**](https://www.mathworks.com/matlabcentral/fileexchange/44308-randanova2)**, this method uses the Still and White's (1981) approach to calculate P-value for interaction effect)** was then implemented on the average activity in the **post cue period** in order to identify the Task related channels, the spatially selective channels as well as the mixed selectivity channels (**p<0.01**). All p values were corrected for multiple comparisons using a Holm-Bonferroni correction. **Note that the proportion of selective cells did not change substantially for different analysis time windows (****supplementary Figure 3).**

**Still AW, White AP. The approximate randomization test as an alternative to the F test in analysis of variance. British Journal of Mathematical and Statistical Psychology 1981, 34, 243–252. doi: 10.1111/j.2044-8317.1981.tb00634.x.**

5) "A two-way ANOVA Task x Position" - was this a non-parametric two-way ANOVA (given the non-parametric Wilcoxon test of the activity between the baseline and a post cue period)? Please specify/justify.

**This is a good point. We have now repeated this analysis using a non-parametric two-way ANOVA (David Stern,** [**https://www.mathworks.com/matlabcentral/fileexchange/44308-randanova2**](https://www.mathworks.com/matlabcentral/fileexchange/44308-randanova2)**). This is now indicated in the methods as follows:**

Spatially selective channels **during the delay epoch** were identified **as follows. First,** a non-parametric Wilcoxon test **was used to identify responsive channels, i.e. channels for which the activities observed during the baseline and during the post cue period (800 to 900 ms following cue onset, only considering trials for which target or fixation off event happened 1000ms after cue presentation) w**ere **significantly different for at least one of the four possible spatial cued positions (p<0.05).** For each responsive channel, a **non-parametric** two-way ANOVA Task x Position **(David Stern,** [**https://www.mathworks.com/matlabcentral/fileexchange/44308-randanova2**](https://www.mathworks.com/matlabcentral/fileexchange/44308-randanova2)**, this method uses the Still and White's (1981) approach to calculate P-value for interaction effect)** was then implemented on the average activity in the **post cue period** in order to identify the Task related channels, the spatially selective channels as well as the channels showing both a task effect and a spatial selectivity effect simultaneously (**p<0.01**). All p values were corrected for multiple comparisons using a Holm-Bonferroni correction.

**Figure 2.** **Mixed selectivity corresponds to the ability of a neuronal population to encode simultaneously independent sources of information:** (A) Firing rate of the same MUA in the three tasks aligned on the cue (200 ms before the cue onset to 1000 ms after the cue onset) in exogenous task (blue), endogenous task (green) and memory guided saccade task (red) for preferred (**solid** line) and non-preferred (dash line) position. **The data was z-scored relative to mean and standard deviation of average individual trial responses during baseline (-150 to 0 ms before cue onset).** **Gray box corresponds to the delay period during which neuronal activities were analyzed for subplots C and D.** (B) Mean firing rate of the same neuronal population in the three tasks aligned on cue presentation (200 ms before the cue onset to 1000 ms after the cue onset) in the exogenous task (blue), the endogenous task (green) and the memory guided saccade task (red) for preferred (**solid** line) and non-preferred (dash line) position. **The data was z-scored relative to mean and standard deviation of average individual channel responses during baseline (-150 to 0 ms before cue onset).** **Gray box corresponds to the delay period during which neuronal activities were analyzed for subplots C and D.** (C) Venn diagram representing the proportion of cells showing a spatial tuning in each of the three tasks. (D) Pie chart corresponding to the proportion of neurons tuned to only position (red), task (blue) and both (mixed-selectivity; yellow). Non-selective neurons are plotted in grey. **Wilcoxon ranksum test was performed between baseline and time interval for each channel and position across trials. A non-parametric 2-Way-ANOVA was performed on selective channels with Bonferroni_Holm correction on spatial and task factor (p<0.01).**

6) Please explain how was MUA normalized (e.g. for the Figure 2A, B and further analyses). It is mentioned that "To extract the averaged firing rates, these epoched MUA data were baseline corrected (independently for each channel and each trial) relative to the time period from -500 ms to -300 ms before the cue.", but baseline subtraction would not result in the arbitrary units shown in Figure 2. What are these units, and why the activity is not shown in Hz, as in Figures 3 and 4?

**We apologize for lack of clarity. Indeed, this text refers to preprocessing for the PCA or dPCA analysis and figure 2ab involves a different normalization. This is now specified in the text as follows:**

[…] In each task, MUA activity was epoched per trial. Spike trains were filtered with a Gaussian kernel (δ = 60 ms). First, MUA activity previous to the cue onset was epoched from -500 ms to 0 with respect to the onset of the Cue for the Pre-Cue analysis. Then, MUA activity was epoched between 500 ms to 0 ms prior to Target onset for the Delay analysis. To extract the averaged firing rates **for** **the dPCA analysis**, these epoched MUA data were baseline corrected (independently for each channel and each trial) relative to the time period from -500 ms to -300 ms before the cue. **This procedure reduced trial-to-trial variability in baseline activity levels.**

**Figure 2. Mixed selectivity corresponds to the ability of a neuronal population to encode simultaneously independent sources of information:** (A) Firing rate of the same MUA in the three tasks aligned on the cue (200 ms before the cue onset to 1000 ms after the cue onset) in exogenous task (blue), endogenous task (green) and memory guided saccade task (red) for preferred (**solid** line) and non-preferred (dash line) position. **The data was z-scored relative to mean and standard deviation of average individual trial responses during baseline (-150 to 0 ms before cue onset).** **Gray box corresponds to the delay period during which neuronal activities were analyzed for subplots C and D.** (B) Mean firing rate of the same neuronal population in the three tasks aligned on cue presentation (200 ms before the cue onset to 1000 ms after the cue onset) in the exogenous task (blue), the endogenous task (green) and the memory guided saccade task (red) for preferred (**solid** line) and non-preferred (dash line) position. **The data was z-scored relative to mean and standard deviation of average individual channel responses during baseline (-150 to 0 ms before cue onset).** **Gray box corresponds to the delay period during which neuronal activities were analyzed for subplots C and D.** (C) Venn diagram representing the proportion of cells showing a spatial tuning in each of the three tasks. (D) Pie chart corresponding to the proportion of neurons tuned to only position (red), task (blue) and both (mixed-selectivity; yellow). Non-selective neurons are plotted in grey. **Wilcoxon ranksum test was performed between baseline and time interval for each channel and position across trials. A non-parametric 2-Way-ANOVA was performed on selective channels with Bonferroni_Holm correction on spatial and task factor (p<0.01).**

**Supplementary Figure 1.** (A) Firing rate of **a representative** MUA (same as in Figure 1) aligned to target onset (400 ms before target onset to 600 ms after target onset) averaged across trials of the exogenous attention task (blue), the endogenous attention task (green) and the memory guided saccade task (red) for preferred (full line) and least-preferred (dash line) positions. **The data was z-scored relative to mean and standard deviation of average individual trial responses during baseline (-150 to 0 ms before cue onset).** (B) Mean firing rate across the neuronal population (MUA) aligned on the target (-400 ms before target onset to 600 ms after target onset) as a function of tasks and positions (same as in A). **The data was z-scored relative to mean and standard deviation of average individual channel responses during baseline (-150 to 0 ms before cue onset).**

7) In terms of the task design, I am surprised the authors did not match the spatial display of the memory-guided task to the other two tasks better, by placing the 4 placeholders identical to the attention tasks target/distractors and indicating the actual saccade target with a brief cue. Without this matching, the tasks differ not only in cognitive demands, but also in the low-level visual stimulation. This might represent a potential confound, since the task-selectivity might at least partially stem from the sensory spatial information, even before cue onset. Please explain this design choice and provide counter-arguments as to why this does not invalidate the results on the task encoding. It seems that there were only 4 possible positions in each session anyway, so showing 4 placeholders would not invalidate the spatial memory component of the actually cued location.

**We thank reviewer 2 for this comment. In the spatial memory task, monkeys had to produce a spatially precise eye movement towards the memorized location. The presence of a landmark would have changed the computations towards the selection of one landmark (aka target) and the execution of an eye movement towards this selected landmark. This operation would have been distinct from that targeted with the current task, i.e. holding in working memory the precise location of a memorized target. In the attention tasks, monkeys had to detect a cued sensory event (change in luminosity of the landmark) while ignoring distractors, and produce a manual response on detection. Placeholders were introduced in these tasks because monkeys also performed in a different set of sessions a variant of these tasks in which distractors could be presented at unpredictable locations anywhere in the screen (Di Bello et al., Cerebral Cortex, 2022). This being said, prefrontal cortical regions are shown to hold a salience map of the most relevant items of the environment. This includes abrupt onset stimuli as well as behaviorally relevant stimuli. Non-informative stimuli such as the landmarks, have thus been shown not to be represented in the FEF (Thompson and Bichot, 2005). As a result, we do not expect these stable landmarks to contribute to the neuronal variance extracted by the dPCA. We do acknowledge that this deserves a discussion.**

**This point is now discussed in the main text as follows: ‘****It is worth noting that in addition, to the fact that attention tasks and memory guided saccade task relied on different cognitive processes, they also differed in that the cued location was indicated by a placeholder in the attentional task, but not in the memory guided saccade task. This can potentially have induced a low-level bias between the two sets of tasks. However, we consider this as unlikely due to the fact that the FEF has been shown to hold a salience map of the most relevant items of the environment. This includes abrupt onset stimuli as well as behaviorally relevant stimuli. Non-informative stable stimuli such as the landmarks, have thus been shown not to be represented in the FEF after their initial processing (Thompson and Bichot, 2005). As a result, we do not expect these stable landmarks to contribute to the neuronal variance extracted by the dPCA’.**

**Thompson**  [**KG**](https://pubmed.ncbi.nlm.nih.gov/15581711/#full-view-affiliation-1)**,**  [**Bichot**](https://pubmed.ncbi.nlm.nih.gov/?sort=date&term=Bichot+NP&cauthor_id=15581711) **NP. A visual salience map in the primate frontal eye field. Prog Brain Res, 2005, 147:251-62. doi: 10.1016/S0079-6123(04)47019-8.**

8) Results: Page 8: It seems counterintuitive that the hit rate in M1 was lowest for arguably the simplest Mem task. Why was this task so demanding for M1? This also seems to be in conflict with the statement in page 6 "while the memory-guided saccade task was always presented in the third order, as monkeys displayed a strong behavioural preference for this task." - how was this preference assessed?

**Monkeys were initially trained on the attention tasks and were reinforced for maintaining a very stable fixation. The memory guided saccade task was introduced next. Monkeys found this new task less demanding producing a lot of trials in a row. However, they still were not very precise in locating the memorized stimulus, hence the high error rate. When this task was introduced first, they refused to work on the attentional task.**

**The main text was modified as follows:** “while the memory-guided saccade task was always presented in the third order, as monkeys displayed a strong behavioral preference for this task **-though low success rate due to not sufficiently precise saccades-** **and refused to work on the attentional tasks when these were presented after the memory guided saccade task”.**

9) Please explain the usage of the nonparametric Friedman test here (i.e. what are repeated measures, what are degrees of freedom, is this analysis across sessions, target locations, etc).

**We really appreciate this comment and acknowledge we have not been clear enough on this point. In this context, we utilized Friedman tests to discern significant variations in task performances for each monkey. Two separate Friedman tests were conducted, one for M1 and another for M2. In each test, we compared behavioral parameters, specifically Hits and Reaction Time, across all sessions in the three tasks. False alarms, indicating responses to distractors, were excluded as they are exclusive to the two attentional tasks. To assess significant differences in false alarm performances within the attentional tasks, we directly applied a Wilcoxon test (rank sum).**

**The repeated measure for the Friedman tests is the number of tasks (n=3). Subsequently, we employed Wilcoxon tests (rank sum) in post-hoc task comparisons to identify significant behavioral differences. It is noteworthy that all statistical tests were conducted independently of the target location, and the degrees of freedom (df) were determined by the number of tasks minus one (i.e., df = 2). We apologize for the error in reporting the degree of freedom.**

**Main text was modified as follows:** Both monkeys showed different behavioural patterns as a function of the type of task they performed. Non-parametric Friedman test **(Tasks as repeated measures, df=2, performed across sessions)** revealed that hit rates (M1: χ2(2) = 12.29, p = 0.002; M2: χ2(2) = 7.6, p = 0.0224) and reaction times (RT) (M1: χ2(2) = 12.29, p = 0.0021; M2: χ2(2) = 7.6, p = 0.0224) were different across tasks (Figure 1B). This reflects different task demands.

10) Page 10: "Indeed, we found a significant task-effect on the response to the preferred position (nonparametric Friedman test χ2(5) = 39.3, p = 2.89e-09)." Please explain the usage of the nonparametric Friedman test here (what are repeated measures, what are degrees of freedom, etc). Is this statement about the example channel in Figure 2A, or the entire population? How is this related to the two-way ANOVA on the task and position?

**Our focus in this analysis is on between-task variability. Consequently, we conducted a comparison of the MUA neuronal population at the preferred position associated with the three tasks, revealing a significant difference among these activities. To achieve this, we employed a Friedman test with the number of tasks (n=3) as the repeated measure and a degree of freedom (df) of 2. We apologize for the error in reporting the degree of freedom.**

**Main text was modified as follows:** Indeed, we found a significant task-effect**, across the entire population,** on the response to the preferred position (non-parametric Friedman test, **performed across MUAs, with tasks as repeated measure,** χ^2^(~~5~~**2**) = 39.3, p = 2.89e-09).

11) Please illustrate the channels of all 3 types. I assume the channel illustrated in the Figure 2A is "mixed selective", please show also only spatial position-selective and task-selective channels. I would also strongly recommend splitting the population into the 3 categories and showing both, the example channel and the population, for all 3 cases.

**We have now generated a supplementary figure S2 that shows three single channels MUA selective to task, position or with mixed-selectivity, respectively, as well as the corresponding population responses. This figure is now cited in the main text in support of Figure 2. This figure and its legend are duplicated below.**

**Supplementary Figure 2: (A) Single MUA example of task selectivity (left), position selectivity (middle) and mixed-selectivity (right). (B) Population activity for task selective MUA (left), position selective MUA (middle) and mixed-selectivity MUA (right).** **Gray box corresponds to the delay period during which neuronal activities were analyzed for subplots C and D of figure 2.**

**This is called in the main text as follows: Supplementary Figure 2 represents a single cell example and the population MUA activity for each category of MUAs.**

12) Figure 2C,D - from which trial periods these analyses are derived? I was expecting the same period as the dPCA analysis (500 ms to 0 ms before target onset) for consistency (but see the later comments on other task intervals).

**In new supplementary figure 3, we now present the same analyses as current figure 2CD for several intervals: Early Cue (0 to 500 ms after the cue); Delay (500 to 1000 ms after the cue); Pre Target (-500 to 0 ms before the target) and Post Target (0 to 500 ms after the target). This supplementary figure 3 is now called in the main text in support of figure 2** **[This categorization was robust against possible sources of noise in the data (supplementary material M1) and did not depend on the analysis window (supplementary Figure 3)], and it is duplicated with its legend below.**

**Supplementary Figure 3: Top row: Venn diagram representing the proportion of cells showing a spatial tuning in each of the three tasks. Bottom row: pie chart corresponding to the proportion of neurons tuned to only position (red), task (blue) or both (mixed-selectivity; yellow) during different periods in the trial (Early Cue: from 0 to 500 ms after the cue onset; Delay: from 500 to 1000 ms after the cue onset; Pre Target: from -500 to 0 ms before the target onset; and Target: from 0 to 500 ms after the target onset). Proportion of non-selective neurons is plotted in grey. This figure complements Figure 2C&D.**

13) Did channels showing exclusively task selectivity in the interval after the spatial information was available - i.e. at some point after the cue - also show task selectivity in the pre-cue interval? Please relate task identity selectivity across these two intervals.

**We thank the reviewer for this suggestion.** **We found that the 94.6% of channels that were exclusively task selective during the delay, were also task-selective in the pre-cue period. This is now implemented in the main text as is.**

14) Generally, I think the results in the Figures 3 and 4 are overall interesting and compelling, but I find a considerable disconnect between the basic - usual standard statistical methods - but largely incomplete analysis in the Figure 2 and the rest, in terms of the time intervals and the level of description. Furthermore, I see no convincing reason to focus only on a single, late delay interval, especially because the analysis in figures 3 and 4 is largely time-resolved anyway. I suggest to expand this analysis to at least two more epochs - pre-cue (as is already done), right after the cue, late delay (as is already done), and response period (e.g. 0 - 500 ms after target onset, or aligned to saccade/manual response) - to capture selectivity profiles of most task-relevant intervals. In fact, I hope that the analysis of the post-cue/early delay period could more consistently highlight the difference / task identity selectivity between the two attentional tasks, because the effect of the exogenous vs endogenous cueing could be more pronounced in the early delay period compared to the late delay where the origin of the attentional cue is already of little relevance.

**Following this reviewer’s recommendation, in new supplementary figure 7, we now present the same analyses as current figure 4 for several intervals: Early Cue (0 to 500 ms after the cue) ; Delay (500 to 1000 ms after the cue) ; Pre Target (-500 to 0 ms before the target) and Post Target (0 to 500 ms after the target). This supplementary figure 7 is now called in the main text in support of figure 4, and it is duplicated with its legend below.**

**Supplementary Figure 7: All as in Figure 4, except that dPCA is performed on a time interval running from (A1 to D1) 0 to 500ms after cue presentation; (A2 to D2) -500 to 0 Pre-target presentation and (A3 to D3) 0 to 500ms Post-target presentation.** **Absolute chance level of the confusion matrix is 1/12=0.08.**

**This figure is now called in the main text as follows: …** (Figure 4, **see supplementary Figure 7 for replication of analyses on different time intervals**).

15) Regarding the interpretation of task identity selectivity, I find the argument "To note, such difference cannot be interpreted as related to the response movement effector (hand response or saccade), since data was analysed during pre-cue activity (prior to motor preparation)." flawed. Because of the block design, monkeys could (and likely did) mobilize the appropriate effectors in a sustained fashion, and perhaps adjusted posture. I think that a large proportion of task selectivity, concerning the most pronounced difference between Mem and manual response attentional tasks, might stem from effector specificity, and I don't think authors can easily dismiss this interpretation. Furthermore, the above argument clearly cannot be applied to the delay period interval, but I don't think the authors try to claim completely different mechanisms of task selectivity for the pre-cue and the delay intervals. Importantly, I don't think this possibility invalidates/compromised the

findings in any way - whether the difference between the tasks is purely in the "cognitive" or/and in the "effector"-specific domain, it is still very interesting.

**We thank reviewer 2 for this insightful comment. At second thought, we do agree with this point. We have now rephrased this part as follows:** To note, such differences **could be due to a reconfiguration of the prefrontal population computations between the different tasks. This reconfiguration could be associated with higher order cognitive factors segregating attentional processes from working memory processes. Alternatively, it could be associated with other task parameters such as effector specificity, the attentional task requiring a manual response while the memory guided saccade task requiring a saccadic response. Further experiments will be needed to precisely identify the computational underlying of these task-related differences.**

16) Partially related to this, I think that spatial working memory process plays a role in all 3 tasks, and not only in the Mem task. Note that the monkeys had to remember which of the 4 locations was cued, in order to ignore distractors and respond to the target. I fully agree that the two attentional tasks present additional cognitive demands compared to the Mem task, but I think the spatial working memory is a largely shared process here, and should not be highlighted as a major discriminant.

**We take this point from reviewer 2. The discussion on this point has now been tuned down as follows:** These three tasks presented a very similar structure (the onset of a cue was followed by a target stimuli). However, they recruited different underlying cognitive mechanisms necessary for their performance. **The two attentional tasks recruited endogenous spatial attention, exogenous spatial attention processes in addition to distractor suppression mechanisms and spatial working memory processes. The memory guided saccade task mostly recruited spatial working memory as well as spatially accurate sensorimotor transformation processes. In the following, we will refer to attention and spatial working memory as the distinguishing features between the two sets of tasks for simplicity.**

### Minor:

1) Page 3: I found the first paragraph of the Introduction quite vague and long-winded, and it did not help me to set up the research question. It also was not convincing stylistically (e.g. 2nd sentence "To do so, …"; 3rd sentence "In doing so,… " and hard to follow. Please consider rewriting. "in the representation of specific task-parameters such as position, color or attention irrespective of the ongoing task" - how can specific task parameters be represented irrespective of the task? Please rephrase/fix logic.

**We changed this paragraph as follows:** The prefrontal cortex (PFC) produces internal representations of our actions and of our environment. **Its** neural computations contribute to the selection, maintenance and filtering of sensory information, in order to integrate the representation of the current task parameters with overarching internal goals and expectations. **As a result**, PFC functionally contributes to several cognitive processes that include executive functions (Friedman and Robbins, 2022; Goldman-Rakic, 1996; Robbins, 1996), attention (Amengual and Ben Hamed, 2021; Ibos et al., 2013; Paneri and Gregoriou, 2017; Rossi et al., 2009), decision making (Domenech and Koechlin, 2015; Funahashi, 2017) and working memory (Funahashi and Kubota, 1994; Lara and Wallis, 2015). ~~Such rich functional architecture is essential to guide the cognitive control of our actions and to shape our behaviour. This vast mosaic-like map of functions demands a very fine functional organization that allows performing multiple computations minimizing interference.~~ **An important question is how PFC implements these different functions both at the single cell and at the populational level.** Electrophysiological studies have shown that PFC displays cells tuned to multiple task- and behaviour-related parameters, a property called non-linear mixed selectivity, which provides computational advantages for information transmission to downstream neurons (Fusi et al., 2016; Rigotti et al., 2013). **In particular, mixed-selectivity allows a given neuronal population to code multiple variables (or cognitive functions) with minimal interference of one variable onto the readout of the other variables. Indeed, n**on-linear mixed-selectivity has been proposed to result in a high-dimensional representation of multiple sensory information patterns of stimuli (such as position, colour or shape) (Rigotti et al., 2013). Such encoding strategy allows simple readout of complex patterns of behaviour, using for example linear classifiers. In the present study, we aim to describe **whether and** how this mixed-selectivity ~~allows the PFC to hold a representation of the ongoing task in addition to a representation of sensory information that dynamically changes as a function of specific task demands.~~ **contributes to the coding of task-identity in the PFC, articulates task-identity coding with that of spatial information and possibly facilitates flexible adjustment of the representation of spatial factors to specific task demands.**

~~One important question is whether non-linear mixed selectivity cells in the PFC are merely involved in the representation of specific task-parameters such as position, color or attention irrespective of the ongoing task, or whether these cells represent higher cognitive levels of encoding by additionally representing task-identity, i.e. specific parameters related to the ongoing task such as the implementation of a specific cognitive process (e.g. attention versus spatial working memory). If task-identity is represented, is this information represented at the same hierarchical level as specific task parameters (i.e. independently), or is task identity represented at a higher hierarchical level (i.e. task information changing for example how the spatial location of stimuli is represented)?~~

2) Page 4: "Two male rhesus monkeys (Macaca mulatta) weighing between 6-8 kg underwent a unique surgery during" - unique in what sense - single surgery, or special / unusual surgery? If the latter, please specify the uniqueness.

**Corrected:** Two male rhesus monkeys (Macaca mulatta) weighing between 6-8 kg underwent a **single** surgery during which they were implanted with two MRI compatible PEEK recording chambers placed over the left and the right FEF hemispheres respectively, as well as a head fixation post.

3) "isomorphic scan" - you probably mean "isotropic"

**Corrected:** A 0.6mm **isotropic** anatomical MRI scan was acquired post surgically on a 1.5T Siemens Sonata MRI scanner

4) Page 6: Baseline corrected – typo

**Corrected:** To extract the averaged firing rates, these epoched MUA data were **baseline corrected** (independently for each channel and each trial) relative to the time period from -500 ms to -300 ms before the cue.

5) Page 7: "of two different macaque rhesus monkeys" - no need for "different"

**Corrected:** To this end, we recorded the MUA from bilateral monkey frontal eye field (FEF), an area involved in the control of covert spatial attention (Armstrong et al., 2009; Gregoriou et al., 2009; Ibos et al., 2013; Paneri and Gregoriou, 2017) and saccades (Hanes et al., 1995; Schall, 2004, 1991; Vernet et al., 2014), of two **~~different~~** macaque rhesus monkeys while they performed three different tasks: an endogenous attention task (Endo), an exogenous attention task (Exo) and a memory-guided saccade task (Mem).

6) Page 8: "While monkeys needed to enhance visual attention in the cued quadrant to respond to the target onset in the exogenous and endogenous attentional tasks (Amengual et al., 2022; Astrand et al., 2020, 2016), they needed to memorize the location of the cue to produce an oriented eye movement towards that location." - add "in the memory-guided saccade task" at the end.

**Corrected:** While monkeys needed to enhance visual attention in the cued quadrant to respond to the target onset in the exogenous and endogenous attentional tasks (Amengual et al., 2022; Astrand et al., 2020, 2016), they needed to memorize the location of the cue to produce an oriented eye movement towards that location **in the memory-guided saccade task**.

7) Figure 2 legend: "full line" - "solid line".

**Corrected.**

8) Figure 2A,B: what is denoted by the gray shaded boxes?

**We apologize for this oversight. Changed as follows in main text and figure legends:**

Specifically, we observed that some cells were recruited during the performance of the three tasks during the delay period (Figure 2A**, gray box**).

**Figure 2.** **Mixed selectivity corresponds to the ability of a neuronal population to encode simultaneously independent sources of information:** (A) Firing rate of the same MUA in the three tasks aligned on the cue (200 ms before the cue onset to 1000 ms after the cue onset) in exogenous task (blue), endogenous task (green) and memory guided saccade task (red) for preferred (**solid** line) and non-preferred (dash line) position. **The data was z-scored relative to mean and standard deviation of average individual trial responses during baseline (-150 to 0 ms before cue onset).** **Gray box corresponds to the delay period during which neuronal activities were analyzed for subplots C and D.** (B) Mean firing rate of the same neuronal population in the three tasks aligned on cue presentation (200 ms before the cue onset to 1000 ms after the cue onset) in the exogenous task (blue), the endogenous task (green) and the memory guided saccade task (red) for preferred (**solid** line) and non-preferred (dash line) position. **The data was z-scored relative to mean and standard deviation of average individual channel responses during baseline (-150 to 0 ms before cue onset).** **Gray box corresponds to the delay period during which neuronal activities were analyzed for subplots C and D.** (C) Venn diagram representing the proportion of cells showing a spatial tuning in each of the three tasks. (D) Pie chart corresponding to the proportion of neurons tuned to only position (red), task (blue) and both (mixed-selectivity; yellow). Non-selective neurons are plotted in grey. **Wilcoxon ranksum test was performed between baseline and time interval for each channel and position across trials. A non-parametric 2-Way-ANOVA was performed on selective channels with Bonferroni_Holm correction on spatial and task factor (p<0.01).**

**Supplementary Figure 1.** (A) Firing rate of **a representative MUA** (same as in Figure 1) aligned to target onset (400 ms before target onset to 600 ms after target onset) averaged across trials of the exogenous attention task (blue), the endogenous attention task (green) and the memory guided saccade task (red) for preferred (**solid** line) and least preferred (dash line) positions. (B) Mean firing rate across the neuronal population (MUA) aligned on the target (-400 ms before target onset to 600 ms after target onset) as a function of tasks and positions (same as in A).

9) Figure 4 legend: "Demixed principal components show similar explain variance as PCA." - typo, "explained".

**Corrected. Thanks.**

10) Figure 4C - time axis labels are completely confused, there are three different variants (time to target, time from target, time from cue) and only the first one is correct, I believe.

**We have now corrected the time labels in Figure 4C. Below is a reproduction of corrected figure 4.**

11) Suppl. Figure 1: since it is MUA, I suggest to avoid usage of "Single Neuronal Activity" or "single neuron".

**Indeed. We have changed the legends and labels of Suppl. Figure 1 accordingly. The final figure is reproduced below.**
