# Supplementary material for: The prefrontal cortex encodes task-identity information and flexibly adjusts its sensory processes as a function of the specific ongoing task
Source: PLoS Biol. 2025 Aug 26;23(8):e3003353. doi: 10.1371/journal.pbio.3003353 (PMC12463330; doi:10.1371/journal.pbio.3003353)
Supplement: Attachment — Submitted filename: Reponse_R2_final.docx [file pbio.3003353.s018.docx]

**We would like to thank both reviewers and the editor for their positive assessment of our work and the constructive comments and insightful questions. We believe that this paper is now improved thanks to their feedback on our work.**

**Please find below a point-by-point response to the reviewers’ comments. Reviewers’ questions are in** black normal fonts**. Responses are in bold blue font and changes made to original version of the manuscript are in *italic bold blue font*. Text from the original version of the manuscript is in** normal blue font**.**

**Major changes to the manuscript are partial spike sorting and reproduction of some of our observations on SUAs. Complete spike sorting was not possible, due to the professional situation of the first author. In addition, due to personal contingencies, the co-last author order was swapped around, with agreement of each of these authors.**

**In addition, a new paragraph in the discussion was added in order to refer to recent modeling work based on recurrent neuronal networks literature that precisely matches the main empirical findings reported in the present paper. This new discussion paragraph reads as follows:**

**“*Our findings resonate with recent computational modeling work by*** ***Driscoll et al. (2024), who propose that recurrent neural networks (RNNs), such as those described in prefrontal cortical circuits, utilize* shared dynamical motifs*—recurring patterns of neural activity implementing specific computations through dynamics such as attractors, decision boundaries, and rotations—to encode task-related information and support flexible multitasking. In their study, RNNs were trained on a diverse set of cognitive tasks commonly studied in humans and animals (Dubreuil et al., 2022; Márton et al., 2022; Yang et al., 2019b). Dynamical motifs clustered in state-space according to task similarity, enabling rapid learning and reconfiguration when faced with task variants. Our empirical evidence of task-state encoding in the prefrontal cortex aligns with these predictions of a modular and compositional neural architecture. Indeed, we describe a lower-dimensional representation of both task identity and task proximity, suggesting a comparable reuse of prefrontal dynamical motifs to enable efficient and flexible task representation and switching, as observed in the RNNs.*”**

*Associated references:*

Driscoll, L.N., Shenoy, K., Sussillo, D., 2024. Flexible multitask computation in recurrent networks utilizes shared dynamical motifs. Nat. Neurosci. 27, 1349–1363. https://doi.org/10.1038/s41593-024-01668-6

Dubreuil, A., Valente, A., Beiran, M., Mastrogiuseppe, F., Ostojic, S., 2022. The role of population structure in computations through neural dynamics. Nat. Neurosci. 25, 783–794. https://doi.org/10.1038/s41593-022-01088-4

Márton, C.D., Gagnon, L., Lajoie, G., Rajan, K., 2022. Efficient and robust multi-task learning in the brain with modular latent primitives. https://doi.org/10.48550/arXiv.2105.14108

Yang, G.R., Joglekar, M.R., Song, H.F., Newsome, W.T., Wang, X.-J., 2019. Task representations in neural networks trained to perform many cognitive tasks. Nat. Neurosci. 22, 297–306. https://doi.org/10.1038/s41593-018-0310-2

## Reviewer #1:

The authors addressed most of the points I raised in a satisfactory manner.

**We thank Reviewer 1 for the positive evaluation of our work.**

There is one point that is still not clear to me in the data preprocessing for multi-unit activity as shown in Fig. 2A, B. In Fig. 2A where an example MUA is shown it is now mentioned that "The data was z-scored relative to mean and standard deviation of average individual trial responses during baseline (-150 to 0 ms before cue onset)." I understand that this refers to the average across individual trials for this channel. However, the baseline level is around 2. I would have expected it to be around 0 if the mean baseline was subtracted. Am I missing something? Similarly, in Fig. 2B where the population average is shown it is around 0.2 instead of 0. Could you please explain why?

**We apologize for this mistake in preparing the figure for the printed version. The correct figure can be directly generated from the OSF files at the following link:** [**https://osf.io/z8eh9/**](https://osf.io/z8eh9/) **and is as expected by Reviewer #1, around zero. The figure has been corrected accordingly. The corrected figure is pasted below. In response to Reviewer #2, point 3a, panel 2D additionally segregates the mixed selectivity category into additive and interaction categories. Last, following the recommendation of reviewer #2 in point 4, the same normalization epoch ([-500ms -300ms] precue) was used when processing the single channel/unit activity and the dPCA. This new figure 2 is duplicated below.**

**Figure 2. Mixed selectivity corresponds to the ability of a neuronal population to encode simultaneously independent sources of information:** (A) Firing rate of a representative MUA aligned on the cue (200 ms before the cue onset to 1000 ms after the cue onset) in exogenous task (blue), endogenous task (green) and memory guided saccade task (red) for preferred (solid line) and non-preferred (dash line) position. ***The data were baseline corrected (-500 to 0 ms before cue onset) on individual trial and channel.*** Gray box corresponds to the delay period during which neuronal activities were analyzed for subplots C and D. (B) Mean firing rate of the MUA neuronal population in the three tasks aligned on cue presentation (200 ms before the cue onset to 1000 ms after the cue onset) in the exogenous task (blue), the endogenous task (green) and the memory guided saccade task (red) for preferred (full line) and non-preferred (dash line) position. ***The data were baseline corrected (-500 to -300 ms before cue onset) on individual trial and channel****.* Gray box corresponds to the delay period during which neuronal activities were analyzed for subplots C and D. (C) Venn diagram representing the proportion of cells showing a spatial tuning in each of the three tasks. (D) Pie chart corresponding to the proportion of neurons tuned to only position (red), task (blue) and ***both (Additive mixed-selectivity; yellow; Mixed-selectivity with interaction; green).*** Non-selective neurons are plotted in grey. Wilcoxon ranksum test was performed between baseline ***(-100 to 0 before the cue onset)*** and time interval ***(800 to 900 ms after the cue onset)*** for each channel and position across trials. A non-parametric 2-Way-ANOVA was performed on selective channels with Bonferroni_Holm correction on spatial and task factor ***on time interval from 600 to 900 ms after the cue onset*** (p<0.01).

**The same figure but displaying SUA data is now presented as main supplementary figure 2. It also includes SUA data aligned on cue, thus mirroring supplementary figure 3 (which shows MUA data aligned on target). Supplementary figure 2 is duplicated below.**

**Supplementary Figure 2. Mixed selectivity corresponds to the ability of a neuronal population to encode simultaneously independent sources of information:** (A) Firing rate of a representative SUA aligned on the cue (200 ms before the cue onset to 1000 ms after the cue onset) in exogenous task (blue), endogenous task (green) and memory guided saccade task (red) for preferred (solid line) and non-preferred (dash line) position. ***The data were baseline corrected (-500 to 0 ms before cue onset) on individual trial and channel.*** Gray box corresponds to the delay period during which neuronal activities were analyzed for subplots C and D. ***(B) Firing rate of a representative SUA (same as in A) aligned on the target (500 ms before the target onset to 500 ms after the target onset). All else as in A.*** (C) Mean firing rate of the SUA neuronal population in the three tasks aligned on cue presentation (200 ms before the cue onset to 1000 ms after the cue onset) in the exogenous task (blue), the endogenous task (green) and the memory guided saccade task (red) for preferred (full line) and non-preferred (dash line) position. ***The data were baseline corrected (-500 to -300 ms before cue onset) on individual trial and channel****.* Gray box corresponds to the delay period during which neuronal activities were analyzed for subplots C and D. ***(D) Firing rate of the SUA neuronal population (same as in C) aligned on the target (500 ms before the target onset to 500 ms after the target onset). All else as in C.*** (E) Venn diagram representing the proportion of cells showing a spatial tuning in each of the three tasks. (F) Pie chart corresponding to the proportion of neurons tuned to only position (red), task (blue) and ***both (Additive mixed-selectivity; yellow; Mixed-selectivity with interaction; green).*** Non-selective neurons are plotted in grey. Wilcoxon ranksum test was performed between baseline ***(-100 to 0 before the cue onset)*** and time interval ***(800 to 900 ms after the cue onset)*** for each channel and position across trials. A non-parametric 2-Way-ANOVA was performed on selective channels with Bonferroni_Holm correction on spatial and task factor ***on time interval from 600 to 900 ms after the cue onset*** (p<0.01).

## Reviewer #2:

Dear authors, dear editor, the manuscript has substantially improved; the revision clarified or fixed several issues. However, four major questions remain unresolved, and I believe they need to be carefully addressed to make the study convincing enough for publication in PLoS Biology. Additionally, there are still parts of the writing that are unclear or confusing. The manuscript also contains several stylistic inconsistencies and typos, so a thorough review of the text is recommended to ensure clarity and consistency.

**Below is a point-by-point response to Reviewer #2 final comments. A major change in the manuscript is the partial spike sorting performed on some sessions, throughout the entire duration of each session (i.e. across all three tasks), as requested by Reviewer #2. We have decided to present the initial MUA analyses in the main manuscript and duplicate the analyses with SUA in supplementary material. In addition, the text was thoroughly reviewed for clarity and consistency.**

### Major:

#### Point 1:

Claims of mixed selectivity using MUA. I am afraid I am not fully convinced by the authors' response that "A recent publication indicates that dimensionality reduction methods are accurate in the absence of spike sorting (Trautmann et al., 2019).". The paper by Trautmann and colleagues shows that the neural population dynamics (neural space trajectories) can be estimated accurately enough without the spike sorting. As they write: "The use of multiunit threshold crossings is not appropriate for establishing whether neurons exhibit selective tuning to one or more stimuli or task parameters. … The experimental neuroscience paradigm proposed here, using threshold crossings, applies to neural population-level analyses. It is not applicable in cases where one wishes to make statements about the properties (e.g., stimulus selectivity) of individual neurons." (Trautmann et al., 2019). In the current manuscript, the authors write, e.g. in the abstract, that "The PFC cells show a non-linear mixed selectivity characterized by specific tuning for multiple task- and behavior related parameters.", so the study is about cells, not about population neural trajectories. As authors themselves state, based on the previous work that used the spike sorting: "Mixed selectivity corresponds to the ability of neurons to simultaneously encode different sources of information (Fusi et al., 2016; Rigotti et al., 2013)." All in all, mixed selectivity is the property of individual neurons. I think it goes without saying that the FEF "population response" would represent both the tasks (which are quite different in a number of features, including very important aspect of the eye vs hand effector for the FEF) as well as the spatial position effects.

a) At least for me, the real question is if and how this information is multiplexed at the level of single neurons.

b) Furthermore, these dimensionality-reduction arguments do not apply at all to the ANOVA-based results, which are currently an integral part of the manuscript. Therefore, I think the authors should provide clear evidence for the mixed selectivity using spike-sorted data. I know it is a lot of work, so perhaps it can be done on a subset of single neurons with the best SNR. A consistent spike-sorting throughout the entire duration of a session (i.e. across all three tasks) will also allow addressing the major point 2 below.

**While we do not question the relevance of the reviewer’s point on whether mixed-selectivity can be properly assessed in MUA as compared to SUA, we would like to emphasize the following point. The core of the paper addresses lower dimensional coding states and neuronal trajectories captured during the cue-to-target epochs of the tasks. The investigation of mixed-selectivity properties is used as a predictor of higher-dimensional representation of information, a point that is confirmed by the dPCA analysis, and to which the above Trautmann et al. 2019 quoted statements apply.**

**As requested by Reviewer #2, the spike sorting was performed on some representative sessions, throughout the entire duration of each session (i.e. across all three tasks). This is now described in the methods section as follows:**

“[…] ***Spike sorting was performed, on a subsample of sessions, using the Offline Sorter (Plexon inc.), on the broadband time signal (bandpass filtered between 300Hz and 3000Hz) of the entire session prior to assigning the signal to the different tasks. Only spikes that could be unambiguously assigned to a same neuron throughout all the tasks were used for the SUA-based analyses. The “Clusters vs. Time” view was used (using the most informative principal component pairs) to check how the waveform changed as a function of time throughout the session. Only units that could be continuously tracked through the entire session, with no drift or a smooth continuous drift in PCA cluster attributes were used for the SUA-based analyses. Examples of spike sorted waveforms tracked in the three different tasks are presented in supplementary figure 1.*** […]”

**Supplementary Figure 1 and its legend are reproduced below:**

***Supplementary Figure 1. Exemplar waveforms for spike sorted single units (SUA) sampled on different channels and sessions. Mean (+/- s.e.) of each waveform is presented per task (Blue: Exogenous attentional task; Green: Endogenous attentional task; Red: Memory guided saccade task).***

**All analyses were reproduced on the spike sorted data, as will be present throughout this revision, except when mentioned otherwise. In particular, we have now performed the mixed-selectivity analysis on sorted data, extracting, for a subsample of sessions, single unit activity (SUA) during the same delay interval used for the MUA analysis (from 600 to 900 ms after cue onset). Although a higher proportion of task-specific SUA is observed compared to the MUA data, a significant proportion of mixed selectivity is still evident for SUA whereby up to 29% of SUA are involved in at least two out of three tasks. Van plots are reproduced below for SUA (left) and MUA (right). MUA data is presented in Figure 2. SUA data is presented in Supplementary Figure 2.**

**SUA mixed-selectivity (Figure S2E):	MUA mixed-selectivity (Figure 2C):**

**As requested by Reviewer #2, the spike sorting was performed on a subsample of sessions, throughout the entire duration of each session (i.e. across all three tasks). The ANOVA-based analyses were subsequently performed on these SUA data. Results are reported in supplementary figure 2 and associated text, as well as in supplementary figure 5. For the analyses in panels A and B of both figure 2 and panels A-D of supplementary figure 2, we adjusted the baseline correction as requested by the reviewer, removing the averaged baseline (from -500 ms to -300 before cue onset) from individual trials and SUA/channels. Additionally, as described above, we conducted the mixed-selectivity analysis on SUA (Figure S2E) / MUA (Figure 2C) and supplemented these analyses with the channel selectivity analysis, incorporating the interaction factor of mixed selectivity on SUA (Figure S2F) / MUA (Figure 2D) as requested by the reviewer in a separate comment. MUA data is presented in figure 2. SUA data is presented in supplementary figure 2.**

**New supplementary figure 2 and associated legend is duplicated below.**

**Supplementary Figure 2. Mixed selectivity corresponds to the ability of a neuronal population to encode simultaneously independent sources of information:** (A) Firing rate of a representative SUA aligned on the cue (200 ms before the cue onset to 1000 ms after the cue onset) in exogenous task (blue), endogenous task (green) and memory guided saccade task (red) for preferred (solid line) and non-preferred (dash line) position. ***The data were baseline corrected (-500 to 0 ms before cue onset) on individual trial and channel.*** Gray box corresponds to the delay period during which neuronal activities were analyzed for subplots C and D. ***(B) Firing rate of a representative SUA (same as in A) aligned on the target (500 ms before the target onset to 500 ms after the target onset). All else as in A.*** (C) Mean firing rate of the SUA neuronal population in the three tasks aligned on cue presentation (200 ms before the cue onset to 1000 ms after the cue onset) in the exogenous task (blue), the endogenous task (green) and the memory guided saccade task (red) for preferred (full line) and non-preferred (dash line) position. ***The data were baseline corrected (-500 to -300 ms before cue onset) on individual trial and channel****.* Gray box corresponds to the delay period during which neuronal activities were analyzed for subplots C and D*.* ***(D) Firing rate of the SUA neuronal population (same as in C) aligned on the target (500 ms before the target onset to 500 ms after the target onset). All else as in C.*** (E) Venn diagram representing the proportion of cells showing a spatial tuning in each of the three tasks. (F) Pie chart corresponding to the proportion of neurons tuned to only position (red), task (blue) and ***both (Additive mixed-selectivity; yellow; Mixed-selectivity with interaction; green).*** Non-selective neurons are plotted in grey. Wilcoxon ranksum test was performed between baseline ***(-100 to 0 before the cue onset)*** and time interval ***(800 to 900 ms after the cue onset)*** for each channel and position across trials. A non-parametric 2-Way-ANOVA was performed on selective channels with Bonferroni_Holm correction on spatial and task factor ***on time interval from 600 to 900 ms after the cue onset*** (p<0.01).

**New supplementary figure 5 and associated legend is duplicated below.**

**Supplementary Figure 5.** ***A) MUA:*** Top row: Venn diagram representing the proportion of cells showing a spatial tuning in each of the three tasks. Bottom row: pie chart corresponding to the proportion of neurons tuned to only position (red), task (blue) or both (mixed-selectivity; yellow) during different periods in the trial (Early Cue: from 0 to 500 ms after the cue onset; Delay: from 500 to 1000 ms after the cue onset; Pre Target: from -500 to 0 ms before the target onset; and Target: from 0 to 500 ms after the target onset). Proportion of non-selective neurons is plotted in grey. This figure complements Figure 2C&D. ***B) SUA: all as in A. This figure complements Supplementary Figure 2E&F.***

**The main text has now been modified as follows: “*****Supplementary figure 2 duplicates the analyses presented in figure 2 for SUAs.*** Supplementary Figure ***4*** represents a single cell example and the population ***MUA/SUA*** activity for each category of ***MUAs/SUAs***. This categorization was robust against possible sources of noise in the data (supplementary material M1***: A, MUA; B: SUA***). ***For MUA***, selectivity did not depend on the post-cue analysis window (***MUA:*** supplementary Figure ***5A), and only marginally did so for SUA (SUA: supplementary Figure 5B***).**”**

#### Point 2:

Recording (in)stability, fixed thresholding and blocked task design
The authors applied a constant (and arguably, very lenient) 3 times SD threshold to derive MUA spike trains from the filtered data. My concern was that this could lead to different firing rates, and different composition of MUA, due to temporal drifts in the acute linear probe recordings. I take the authors' point that the dPCA can deal with the drifts by assigning the related variance to non-specific components. But the task and the time are not orthogonal in the current design, so for instance the strongest task-related component 3 in Figure 4 could be related to time, as the new analysis in Supplementary material M2 also seems to show. Furthermore, the ANOVA analyses are not at all immune to drifts.

**In our view, Supplementary material by reviewer 2 confirms independence of our report from the confounding time variable. Indeed, the task-dPCA component does distinguish between attentional tasks 1 and 2. Yet, when comparing the normalized firing rate projected onto this component when attentional tasks 1 and 2 are ordered as a function of order of presentation in the session and not as a function of task identity, the task-dPCA component cannot distinguish them anymore, indicating that the task-component is indeed extracting task information rather than task order in the session. This is now made explicit in supplementary material M2 and main text as follows:**

***“******Thus, the task-dPCA component cannot distinguish between first and second task in the session, while it can distinguish between Endo and Exo attentional tasks.”***

a) Therefore, I think illustrative examples of filtered recordings as a function of time (from which the spike crossings were derived) should be shown, and most importantly, the analysis of raw firing rates as a function of time and the task block should be presented, to dissociate the effects of the tasks and potential drift effects. Again, using the well-isolated single neuron data can also help to address this concern, if the spike sorting is stable and accurate enough despite potential amplitude drifts.

**Spike sorting has been implemented as described in response to reviewer #2, point 1b. Spike sorting procedure is now described in main text, and exemplar waveforms tracked in the three tasks are presented in supplementary figure 1.**

b) "A threshold was applied independently for each recording contact and before the actual task-related recordings started, such that the crossing of this threshold resulted in multiunit spike trains. This threshold corresponded to 3 standard deviations from mean activity during unconstrained eye movements by the monkeys while exploring a blank screen. This threshold was confirmed during the offline analyses." - What does "confirmed" mean here? Was the threshold ever adjusted offline? If not, how much it deviated from the initially calculated/guessed 3 SD?

**We apologize for the lack of clarity. We applied a threshold for each channel on the MUA signal during the recording sessions and conducted a sanity check channel by channel to ensure the quality of the MUA signal and the 3 SD criteria. Additionally, the threshold was further adjusted offline during spike sorting analyses to optimize single unit activity extraction for each channel. This readjustment impacted MUA final thresholding values. The main text has now been modified as follows:**

**“**[…] ***This threshold was confirmed during the offline analyses to optimize single unit activity extraction for each channel. This readjustment impacted MUA final thresholding values.*”**

#### Point 3:

Interpretation of ANOVA results and mixed selectivity, relationship to dPCA results.

a) One of the central points the authors advance in this work is the non-linear mixed selectivity. If I understand correctly, this type of mixed selectivity should be reflected in the interaction term of the two-way ANOVA. But the analysis in the Figure 2D combines the units which show an additive combination of the two main effects (presumably corresponding to linear mixed selectivity) and those showing the interaction. Please separate these two effects, which would also allow to relate this to the dPCA interaction component.

**Reviewer #2 is indeed correct. We now changed figure 2D (for MUA), supplementary figure 2E (for SUA), supplementary material M1 (for each of MUA and SUA) and supplementary figure 5 (for each of MUA and SUA), highlighting the proportion of mixed selectivity channels with (non-linear mixed-selectivity) and without interaction (linear mixed-selectivity). These figures are reproduced below and the main text has been modified as follows:**

**“**Based on the statistical significance of the firing rate modulation for these two parameters (spatial selectivity and task selectivity), we found that 31% of cells were uniquely selective for the task, 6% were uniquely selective for position, and 60% showed a mixed selectivity for both parameters**: *38% showed no interaction between task and position (corresponding to a linear mixed-selectivity) and 22% showed an interaction between task and position (corresponding to a non-linear mixed-selectivity -Figure 2D)****.* ***Supplementary figure 2 duplicates the analyses presented in figure 2 for SUAs.*** Supplementary Figure ***4*** represents a single cell example and the population ***MUA/SUA*** activity for each category of ***MUAs/SUAs***. This categorization was robust against possible sources of noise in the data (supplementary material M1***: A, MUA; B: SUA***). ***For MUA***, did not depend on the post-cue analysis window (***MUA:*** supplementary Figure ***5A), and only marginally did so for SUA (SUA: supplementary Figure 5B***).**”**

**New figure 2 and associated legend is duplicated below.**

**Figure 2. Mixed selectivity corresponds to the ability of a neuronal population to encode simultaneously independent sources of information:** (A) Firing rate of a representative MUA aligned on the cue (200 ms before the cue onset to 1000 ms after the cue onset) in exogenous task (blue), endogenous task (green) and memory guided saccade task (red) for preferred (solid line) and non-preferred (dash line) position. ***The data were baseline corrected (-500 to 0 ms before cue onset) on individual trial and channel.*** Gray box corresponds to the delay period during which neuronal activities were analyzed for subplots C and D. (B) Mean firing rate of the MUA neuronal population in the three tasks aligned on cue presentation (200 ms before the cue onset to 1000 ms after the cue onset) in the exogenous task (blue), the endogenous task (green) and the memory guided saccade task (red) for preferred (full line) and non-preferred (dash line) position. ***The data were baseline corrected (-500 to -300 ms before cue onset) on individual trial and channel****.* Gray box corresponds to the delay period during which neuronal activities were analyzed for subplots C and D. (C) Venn diagram representing the proportion of cells showing a spatial tuning in each of the three tasks. (D) Pie chart corresponding to the proportion of neurons tuned to only position (red), task (blue) and ***both (Additive mixed-selectivity; yellow; Mixed-selectivity with interaction; green).*** Non-selective neurons are plotted in grey. Wilcoxon ranksum test was performed between baseline ***(-100 to 0 before the cue onset)*** and time interval ***(800 to 900 ms after the cue onset)*** for each channel and position across trials. A non-parametric 2-Way-ANOVA was performed on selective channels with Bonferroni_Holm correction on spatial and task factor ***on time interval from 600 to 900 ms after the cue onset*** (p<0.01).

**New supplementary figure 2 and associated legend is duplicated below.**

**Supplementary Figure 2. Mixed selectivity corresponds to the ability of a neuronal population to encode simultaneously independent sources of information:** (A) Firing rate of a representative SUA aligned on the cue (200 ms before the cue onset to 1000 ms after the cue onset) in exogenous task (blue), endogenous task (green) and memory guided saccade task (red) for preferred (solid line) and non-preferred (dash line) position. ***The data were baseline*** ***corrected (-500 to 0 ms before cue onset) on individual trial and channel.*** Gray box corresponds to the delay period during which neuronal activities were analyzed for subplots C and D. ***(B) Firing rate of a representative SUA (same as in A) aligned on the target (500 ms before the target onset to 500 ms after the target onset). All else as in A.*** (C) Mean firing rate of the SUA neuronal population in the three tasks aligned on cue presentation (200 ms before the cue onset to 1000 ms after the cue onset) in the exogenous task (blue), the endogenous task (green) and the memory guided saccade task (red) for preferred (full line) and non-preferred (dash line) position. ***The data were baseline corrected (-500 to -300 ms before cue onset) on individual trial and channel****.* Gray box corresponds to the delay period during which neuronal activities were analyzed for subplots C and D. ***(D) Firing rate of the SUA neuronal population (same as in C) aligned on the target (500 ms before the target onset to 500 ms after the target onset). All else as in C.*** (E) Venn diagram representing the proportion of cells showing a spatial tuning in each of the three tasks. (F) Pie chart corresponding to the proportion of neurons tuned to only position (red), task (blue) and ***both (Additive mixed-selectivity; yellow; Mixed-selectivity with interaction; green).*** Non-selective neurons are plotted in grey. Wilcoxon ranksum test was performed between baseline ***(-100 to 0 before the cue onset)*** and time interval ***(800 to 900 ms after the cue onset)*** for each channel and position across trials. A non-parametric 2-Way-ANOVA was performed on selective channels with Bonferroni_Holm correction on spatial and task factor ***on time interval from 600 to 900 ms after the cue onset*** (p<0.01).

**New supplementary figure 4 and associated legend is duplicated below.**

**Supplementary *Figure 4.*** (A) Single MUA example of task selectivity (left), position selectivity (middle) and mixed-selectivity (right). (B) Population activity for task selective MUA (left), position selective MUA (middle) and mixed-selectivity MUA (right). Gray box corresponds to the delay period during which neuronal activities were analysed for subplots C and D of figure 2. ***(C) Single SUA example of task selectivity (left), position selectivity (middle) and mixed-selectivity (right). (D) Population activity for task selective MUA (left), position selective MUA (middle) and mixed-selectivity MUA (right). Gray box corresponds to the delay period during which neuronal activities were analysed for subplots E and F of supplementary figure 2.***

**New supplementary figure 5 and associated legend is duplicated below.**

**Supplementary Figure 5.** ***A) MUA:*** Top row: Venn diagram representing the proportion of cells showing a spatial tuning in each of the three tasks. Bottom row: pie chart corresponding to the proportion of neurons tuned to only position (red), task (blue) or both (mixed-selectivity; yellow) during different periods in the trial (Precue: from 0 to 500 ms before the cue onset; Early Cue: from 0 to 500 ms after the cue onset; Delay: from 500 to 1000 ms after the cue onset; Pre Target: from -500 to 0 ms before the target onset; and Target: from 0 to 500 ms after the target onset). Proportion of non-selective neurons is plotted in grey. This figure complements Figure 2C&D. ***B) SUA: all as in A. This figure complements Supplementary Figure 2E&F.***

b) Additionally, please describe in simple terms how the (non-linear) mixed selectivity influences the preferred spatial position in individual neurons/multiunits? Specifically, please describe whether and how the actual preferred position changes as a function of the task: e.g., as shifts of the preferred position, or changes in the "depth" of spatial tuning (e.g. gain-like task effect) but without the shifts of the preferred position? It would be helpful to show the proportions of units where such changes occur, along with illustrative examples of tuning curves (across all four positions) for both linear and non-linear mixed selectivity units. As a general note, the Results section is very condensed; I suggest taking advantage of the PLoS Biol format to include more than just four figures to better illustrate the findings.

**Taking on the recommendation of reviewer #2, we have now added a main Figure 3 that quantifies some aspects of mixed-selectivity with and without interaction:**

***Figure 3.*** ***Spatial tuning of mixed-selectivity cells. (A) Single MUA example, with mixed-selectivity, and finely-tuned receptive field. Plots represent firing rates (average across trials +/- s.e.) baseline corrected (-500 to 0 ms before cue onset) on individual trial: Exo, blue, Endo, green, Sacc, red. Firing rates at preferred spatial position are significantly different from the other spatial locations for all tasks (p<0.005 or better). (B) Single MUA example, with mixed-selectivity, and board receptive field. All else as in (A). Firing rates at the two preferred spatial positions are significantly different from the other spatial locations for all tasks (p<0.05 or better), except for the Ipsi versus Contra and Diagonal comparison in the Sacc task. (C) Population spatial tuning of mixed-selectivity MUAS with no interaction. Plots represent firing rates (average across MUAs +/- s.e.) baseline corrected (-500 to 0 ms before cue onset) on individual trial: Exo, blue, continuous, MUAs with broad receptive fields (RF), dashed, MUAs with finely-tuned receptive fields, Endo, green, Sacc, red. Firing rates at preferred spatial position are significantly different from the other spatial locations for all tasks and conditions (p<0.005 or better). (D) Population spatial tuning of mixed-selectivity MUAS with interaction. All else as in (D). Firing rates at preferred spatial position are significantly different from the other spatial locations for all tasks and conditions except for the firing rates of the MUAs with broad receptive fields in the Exo task (p<0.001 or better). Firing rates at Ipsi spatial position are significantly different from the Contra and Diag spatial locations for all tasks and conditions except for the Sacc task (p<0.005 or better).***

***Preferred indicates the spatial location eliciting the highest variation in firing rate from baseline. Ipsi indicates the stimulated spatial position that falls in the same hemifield as the Preferred location. Contra indicates the stimulated spatial position that falls in the contralateral hemifield as the Preferred location. Diag indicates the stimulated spatial position that falls in the contralateral hemifield as the Preferred location, diagonally to it.***

**This figure is now called and discussed in the main text as follows: “*Figure 3 aims to characterize the impact of mixed-selectivity, with and without interactions, on attention selectivity spatial indicators prior to target presentation. Some MUAs had a firing rate that was significantly higher for a well-identified preferred position compared to the three other positions in at least one of the three tasks. Such a spatially organized response pattern could be related to the match between our task configuration and the MUAs’ receptive field location, where one of the target positions coincided with the center of the receptive field. However, such a response pattern was almost exclusively identified in the Exo task (Mixed-selectivity without interaction, Exo: 28%; Mixed-selectivity with interaction, Exo: 59%, Endo: 1%). This indicates that, independently of the relative match between task configuration and MUA receptive field, the response pattern changed between the Exo task and the other tasks and became spatially less resolved. This is exemplified in Figure 3, which shows representative single MUAs (Figure 3A, mixed-selective MUA finely tuned receptive field; 3B, mixed selectivity MUA with broad receptive field) and population-level responses (Figure 3C, average population mixed-selective MUAs without interaction; 3D, with interaction). To further analyze the impact of the task on the reconfiguration of the MUAs’ spatial selectivity, we identified, for all mixed-selectivity MUAs, the two spatial positions eliciting the highest firing rates. We then compared the degree of partial overlap across tasks, pairwise. For the vast majority of mixed-selectivity MUAs without interaction, the receptive fields shared at least one spatial position across each pair of tasks (Exo-Endo: 98%; Exo-Sacc: 98%; Endo-Sacc: 97%). This was also the case for the receptive fields of mixed-selectivity MUAs with interaction, with the marked exception of the Endo-Sacc comparison (Exo-Endo: 95%; Exo-Sacc: 96%; Endo-Sacc: 75%). Because the reconfiguration of receptive fields between the Endo and Sacc tasks was specific to this MUA category, it strongly indicates that these MUAs play a distinct role in these tasks. It is worth noting that task order cannot account for these selective changes, not only because they are specific to this MUA mixed-selectivity category, but also because the order of the Exo and Endo tasks was randomized across different sessions. This possibly relates to previous observations describing shifts of receptive fields in the parietal and prefrontal cortex around saccade execution (Ben Hamed et al., 2002; Joiner et al., 2013, 2011; Khanna et al., 2020; Zirnsak and Moore, 2014). Understanding why these shifts seem to be specific to mixed-selectivity MUAs with interaction requires further investigations. Likewise, as these shifts are mostly evident when comparing the Endo and Sacc tasks, it would be relevant to understand in what tasks and context such shifts arise (Zirnsak and Moore, 2014).”***

**References:**

**Ben Hamed, S., Duhamel, J.-R., Bremmer, F., Graf, W., 2002. Visual receptive field modulation in the lateral intraparietal area during attentive fixation and free gaze. Cereb. Cortex N. Y. N 1991 12, 234–45. https://doi.org/11839598**

**Joiner, W.M., Cavanaugh, J., Wurtz, R.H., 2013. Compression and Suppression of Shifting Receptive Field Activity in Frontal Eye Field Neurons. J. Neurosci. 33, 18259–18269. https://doi.org/10.1523/JNEUROSCI.2964-13.2013**

**Joiner, W.M., Cavanaugh, J., Wurtz, R.H., 2011. Modulation of shifting receptive field activity in frontal eye field by visual salience. J. Neurophysiol. 106, 1179–1190. https://doi.org/10.1152/jn.01054.2010**

**Khanna, S.B., Scott, J.A., Smith, M.A., 2020. Dynamic shifts of visual and saccadic signals in prefrontal cortical regions 8Ar and FEF. J. Neurophysiol. 124, 1774–1791. https://doi.org/10.1152/jn.00669.2019**

**Zirnsak, M., Moore, T., 2014. Saccades and shifting receptive fields: anticipating consequences or selecting targets? Trends Cogn. Sci. 18, 621–628. https://doi.org/10.1016/j.tics.2014.10.002**

c) As a control, and again to better relate the results of the ANOVA analysis to subsequent dPCA not only for the post-cue epochs but also for pre-cue epoch, please repeat the same two-way ANOVA analysis on the pre-cue epoch. The expectation would be that the "non-causal" position selectivity should largely disappear, except for some spurious random effects due to false positives.

**This is again a very good point. This analysis has now been implemented for MUAs and SUAs in Supplementary Figure 5 duplicated below. As expected by reviewer #2, only spurious position-related units are identified. This is now mentioned in the main text as follows:**

**“**Based on the statistical significance of the firing rate modulation for these two parameters (spatial selectivity and task selectivity), we found that 31% of cells were uniquely selective for the task, 6% were uniquely selective for position, and 60% showed a mixed selectivity for both parameters**: *38% showed no interaction between task and position (corresponding to a linear mixed-selectivity) and 22% showed an interaction between task and position (corresponding to a non-linear mixed-selectivity -Figure 2D)****.* ***Supplementary figure 2 duplicates the analyses presented in figure 2 for SUAs.*** Supplementary Figure ***4*** represents a single cell example and the population ***MUA/SUA*** activity for each category of ***MUAs/SUAs***. This categorization was robust against possible sources of noise in the data (supplementary material M1***: A, MUA; B: SUA***). ***For MUA***, did not depend on the post-cue analysis window (***MUA:*** supplementary Figure ***5A), and only marginally did so for SUA (SUA: supplementary Figure 5B). As expected, no position-related nor mixed-selectivity MUAs or SUAs were identified during the pre-cue epoch (-500 to 0ms prior to cue presentation, supplementary Figure 5B).*”**

**Supplementary Figure 5.** ***A) MUA:*** Top row: Venn diagram representing the proportion of cells showing a spatial tuning in each of the three tasks. Bottom row: pie chart corresponding to the proportion of neurons tuned to only position (red), task (blue) or both (mixed-selectivity; yellow) during different periods in the trial (Precue: from 0 to 500 ms before the cue onset; Early Cue: from 0 to 500 ms after the cue onset; Delay: from 500 to 1000 ms after the cue onset; Pre Target: from -500 to 0 ms before the target onset; and Target: from 0 to 500 ms after the target onset). Proportion of non-selective neurons is plotted in grey. This figure complements Figure 2C&D. ***B) SUA: all as in A. This figure complements Supplementary Figure 2E&F.***

d) Finally, in the current manuscript the authors use the toolbox by Kobak et al. to generate Figures 3 and 4, and do not go any deeper beyond the basic output. But the dimensions underlying main effects (tasks and position) as well as the non-linear mixed selectivity can be distributed very differently across the units. For instance, if one imagines a 2D grid plot where one axis is units and another dimensions, and the intensity of a cell indicates the "weights" for each unit and each dimension, there could be for instance two different scenarios that both would result in representation of both types of information at the population level. In the first scenario, one group of units can represent e.g. the position dimensions but not the task dimensions, and the complementary group - vice versa, whereas in the second scenario, all units would have some non-zero weights for both types of dimensions. If I understand correctly, the current analysis cannot distinguish between these two scenarios? Did the authors examine the patterns of the weight distributions in their analysis?

**We thank reviewer #2 for this comment. We now produce, in Supplementary Figure 8, the distribution of weights attributed to the different units in the first position-related dPC as a function of the first task-related dPC, for both SUAs (left) and MUAs (right). Both distributions are unimodal, such that most units contribute to the coding of both task- and position-related information. This is now discussed in the main text as follows: “**Similarly to what we found in the previous section, the two first components associated to the task showed respectively a dissociation between attentional task-related processes and working memory task-related process (component 3), and a trend of separation between the two attentional tasks (component 4). In addition, the first component associated to position exhibited a dissociation of each position corresponding to the four expected target locations, whereas the projection onto the second spatial-related component showed two different states possibly corresponding to the segregation of information across hemifields and recording probes (i.e., probes respectively located into right or left FEF) (supplementary Figure 12). ***The distribution of weights attributed to the different units in the first position-related dPC as a function of the first task-related dPC were unimodal, such that most units contribute to the coding of both task- and position-related information (Supplementary Figure 8). This supports the fact that mixed-selectivity contributes to the lower dimensional coding of these two variables.***”

**New supplementary figure 8 and associated legend is duplicated below.**

***Supplementary figure 8. Distribution of weights attributed to the different units in the first position-related dPC as a function of the first task-related dPC, for both SUAs (left) and MUAs (right).***

#### Point 4:

There are a number of inconsistencies / seemingly arbitrary choices in respect to many variants of baseline correction, selected analysis epochs and ANOVA vs dPCA results.

a) Figure 2: "The data was z-scored relative to mean and standard deviation of average individual trial responses during baseline (-150 to 0 ms before cue onset)." - what does "average individual" mean? Why the normalized pre-cue activity level is not around zero given that largely overlapping window -150 to 0 ms was used for the baseline? Same questions about population response in B. I also don't understand why B is not just an average of already normalized responses of all MUA as in A - i.e. why there seems to be different normalization applied?

**This should have read as "**The data was z-scored relative to mean and standard deviation of individual trial responses during baseline (-150 to 0 ms before cue onset).**"** **We apologize for this oversight. This baselining procedure is now not used anymore as per your comment 4.b.**

b) Next, I don't understand why the new Supplementary Figure 2, related to Figure 2, shows a different scale for the "normalized firing rate" - should it not be the same as in the Figure 2A? I also assume that this is not the same normalization that is used for dPCA (see below), because subtracting a baseline should result in in firing rate difference to baseline in Hz, not in very small a.u.?

**We thank reviewer #2 for spotting this inconsistency. For Supplementary Figure 2, we indeed applied a baseline correction similar to the one used for the dPCA analysis by subtracting the averaged baseline signal (from -500 ms to -300 before cue onset) for each trial and channel, without performing a z-score correction as in Figures 2A and 2B. Consequently, we also reanalysed Figures 2A and 2B using the same baseline correction as in Supplementary Figure 2 and the dPCA, and we updated the corrected versions of Figures 2A and 2B in the manuscript. This difference in normalization explains why the firing rate signal was not expressed in Spikes/s in Figures 2A and 2B. We also corrected the y-axis label in Supplementary Figure 2, and applied the baseline correction used in the dPCA analysis for Supplementary figures 2-4.**

**New figure 2 and associated legend is duplicated below.**

**Figure 2. Mixed selectivity corresponds to the ability of a neuronal population to encode simultaneously independent sources of information:** (A) Firing rate of a representative MUA aligned on the cue (200 ms before the cue onset to 1000 ms after the cue onset) in exogenous task (blue), endogenous task (green) and memory guided saccade task (red) for preferred (solid line) and non-preferred (dash line) position. ***The data were baseline corrected (-500 to 0 ms before cue onset) on individual trial and channel.*** Gray box corresponds to the delay period during which neuronal activities were analyzed for subplots C and D. (B) Mean firing rate of the MUA neuronal population in the three tasks aligned on cue presentation (200 ms before the cue onset to 1000 ms after the cue onset) in the exogenous task (blue), the endogenous task (green) and the memory guided saccade task (red) for preferred (full line) and non-preferred (dash line) position. ***The data were baseline corrected (-500 to -300 ms before cue onset) on individual trial and channel****.* Gray box corresponds to the delay period during which neuronal activities were analyzed for subplots C and D. (C) Venn diagram representing the proportion of cells showing a spatial tuning in each of the three tasks. (D) Pie chart corresponding to the proportion of neurons tuned to only position (red), task (blue) and ***both (Additive mixed-selectivity; yellow; Mixed-selectivity with interaction; green).*** Non-selective neurons are plotted in grey. Wilcoxon ranksum test was performed between baseline ***(-100 to 0 before the cue onset)*** and time interval ***(800 to 900 ms after the cue onset)*** for each channel and position across trials. A non-parametric 2-Way-ANOVA was performed on selective channels with Bonferroni_Holm correction on spatial and task factor ***on time interval from 600 to 900 ms after the cue onset*** (p<0.01).

**New supplementary figure 2 and associated legend is duplicated below.**

**Supplementary Figure 2. Mixed selectivity corresponds to the ability of a neuronal population to encode simultaneously independent sources of information:** (A) Firing rate of a representative SUA aligned on the cue (200 ms before the cue onset to 1000 ms after the cue onset) in exogenous task (blue), endogenous task (green) and memory guided saccade task (red) for preferred (solid line) and non-preferred (dash line) position. ***The data were baseline corrected (-500 to 0 ms before cue onset) on individual trial and channel.*** Gray box corresponds to the delay period during which neuronal activities were analyzed for subplots C and D. ***(B) Firing rate of a representative SUA (same as in A) aligned on the target (500 ms before the target onset to 500 ms after the target onset). All else as in A.*** (C) Mean firing rate of the SUA neuronal population in the three tasks aligned on cue presentation (200 ms before the cue onset to 1000 ms after the cue onset) in the exogenous task (blue), the endogenous task (green) and the memory guided saccade task (red) for preferred (full line) and non-preferred (dash line) position. ***The data were baseline corrected (-500 to -300 ms before cue onset) on individual trial and channel****.* Gray box corresponds to the delay period during which neuronal activities were analyzed for subplots C and D. ***(D) Firing rate of the SUA neuronal population (same as in C) aligned on the target (500 ms before the target onset to 500 ms after the target onset). All else as in C.*** (E) Venn diagram representing the proportion of cells showing a spatial tuning in each of the three tasks. (F) Pie chart corresponding to the proportion of neurons tuned to only position (red), task (blue) and ***both (Additive mixed-selectivity; yellow; Mixed-selectivity with interaction; green).*** Non-selective neurons are plotted in grey. Wilcoxon ranksum test was performed between baseline ***(-100 to 0 before the cue onset)*** and time interval ***(800 to 900 ms after the cue onset)*** for each channel and position across trials. A non-parametric 2-Way-ANOVA was performed on selective channels with Bonferroni_Holm correction on spatial and task factor ***on time interval from 600 to 900 ms after the cue onset*** (p<0.01).

**New supplementary figure 3 and associated legend is duplicated below.**

**Supplementary *Figure 3*.** (A) Firing rate of a representative MUA (same as in ***Figure 2***) aligned to target onset (400 ms before target onset to 600 ms after target onset) averaged across trials of the exogenous attention task (blue), the endogenous attention task (green) and the memory guided saccade task (red) for preferred (solid line) and least-preferred (dash line) positions. The data was z-scored relative to mean and standard deviation of average individual trial responses during baseline (-150 to 0 ms before cue onset). (B) Mean firing rate across the neuronal population (MUA) aligned on the target (-400 ms before target onset to 600 ms after target onset) as a function of tasks and positions (same as in A). The data was z-scored relative to mean and standard deviation of average individual channel responses during baseline (-150 to 0 ms before cue onset).

**New supplementary figure 4 and associated legend is duplicated below.**

**Supplementary *Figure 4.*** (A) Single MUA example of task selectivity (left), position selectivity (middle) and mixed-selectivity (right). (B) Population activity for task selective MUA (left), position selective MUA (middle) and mixed-selectivity MUA (right). Gray box corresponds to the delay period during which neuronal activities were analysed for subplots C and D of figure 2. ***(C) Single SUA example of task selectivity (left), position selectivity (middle) and mixed-selectivity (right). (D) Population activity for task selective MUA (left), position selective MUA (middle) and mixed-selectivity MUA (right). Gray box corresponds to the delay period during which neuronal activities were analysed for subplots E and F of supplementary figure 2.***

c) Finally, there is yet another normalization for dPCA: "To extract the averaged firing rates for dPCA analysis, these epoched MUA data were baseline corrected (independently for each channel and each trial) relative to the time period from -500 ms to -300 ms before the cue. This reduced trial-to-trial variability in baseline activity levels and reduces individual trial differences." The question is why a different baseline epoch has been used for dPCA?
"First, MUA activity previous to the cue onset was epoched from -500 ms to 0 with respect to the onset of the Cue for the Pre-Cue analysis." - why to normalize per trial using the window (-500 ms to -300 ms before the cue) that is largely overlapping with the epoch of interest? Do I understand correctly that if you were to normalize in -500 ms to 0 ms before the cue, you would not be able to see pre-cue effects at all?

**See below.**

Alternatively, there is an error in the description above, because the Results text (page 12) says "This analysis was firstly performed during the precue period (200 ms before cue onset to cue onset) and will be later performed during the cue to target interval (500 ms to 0 ms before target onset) to unmix task- from sensory- specific variance." Is the "precue period (200 ms before cue onset to cue onset)" supposed to be the same as "Pre-Cue" epoch above?

**Reviewer #2 is correct. This part is correct -modulo a small mistake:** "This analysis was firstly performed during the precue period (***300*** ms before cue onset to cue onset) and will be later performed during the cue to target interval (500 ms to 0 ms before target onset) to unmix task- from sensory- specific variance."

**The above methodological description was corrected as follows:** “First, MUA activity previous to the cue onset was epoched from **-*300*** ms to 0 with respect to the onset of the Cue for the Pre-Cue analysis. Then, MUA activity was epoched between 500 ms to 0 ms prior to Target onset for the Delay analysis. To extract the averaged firing rates for dPCA analysis, these epoched MUA data were baseline corrected (independently for each channel and each trial) relative to the time period from -500 ms to -300 ms before the cue.”

d) Figure 2: "Gray box corresponds to the delay period during which neuronal activities were analyzed for subplots C and D." - so it is 500 ms for the two-way ANOVA" - so it is 500 ms epoch for two-way ANOVA, on raw firing rates or on normalized (and so, how) firing rates? But for the "Wilcoxon ranksum test was performed between baseline and time interval for each channel and position across trials." - here the "time interval" (please provide better description) is, according to the Methods, is different, 800 - 900 ms after the cue onset: "First, a non-parametric Wilcoxon test was used to identify responsive channels, i.e. channels for which the activities observed during the baseline and during the post cue period (800 to 900 ms following cue onset, only considering trials for which target or fixation off event happened 1000ms after cue presentation) were significantly different for at least one of the four possible spatial cued positions (p<0.05)."

**We thank the reviewer for this comment and apologize for the lack of clarity in our explanation. The two-way ANOVA tests for Figures 2C and 2D were conducted on raw firing rate data, smoothed using a sliding window of 50 ms. For the Wilcoxon test, the selected delay interval was from 800 to 900 ms after cue onset, with a baseline period from -100 to 0 ms before cue onset. The Wilcoxon test between this baseline (-100 to 0 ms before cue onset) and the delay period (800 to 900 ms after cue onset) was used to identify responsive channels. Following this, the responsive channels were statistically analyzed using a two-way ANOVA on the raw firing rate data from the delay interval of 600 to 900 ms after cue onset across trials, based on position and task. This analysis produced three p-values: one for selectivity to the task only, one for selectivity to the position only, and one for the interaction between task and position. The delay intervals for the Wilcoxon test and the two-way ANOVA differ because, for the responsive channels, we compared two intervals of equal length (-100 to 0 ms vs. 800 to 900 ms), whereas the two-way ANOVA compared the signal by task and position within the same cue-to-target interval. This analysis assigned specific selectivity to the task, position, or both parameters. This is now highlighted in the legend of figure 2, as follows:**

**Figure 2. Mixed selectivity corresponds to the ability of a neuronal population to encode simultaneously independent sources of information:** (A) Firing rate of a representative MUA aligned on the cue (200 ms before the cue onset to 1000 ms after the cue onset) in exogenous task (blue), endogenous task (green) and memory guided saccade task (red) for preferred (solid line) and non-preferred (dash line) position. **The data were baseline corrected (-500 to 0 ms before cue onset) on individual trial and channel.** Gray box corresponds to the delay period during which neuronal activities were analyzed for subplots C and D. (B) Mean firing rate of the MUA neuronal population in the three tasks aligned on cue presentation (200 ms before the cue onset to 1000 ms after the cue onset) in the exogenous task (blue), the endogenous task (green) and the memory guided saccade task (red) for preferred (full line) and non-preferred (dash line) position. **The data were baseline corrected (-500 to -300 ms before cue onset) on individual trial and channel**. Gray box corresponds to the delay period during which neuronal activities were analyzed for subplots C and D. (C) Venn diagram representing the proportion of cells showing a spatial tuning in each of the three tasks. (D) Pie chart corresponding to the proportion of neurons tuned to only position (red), task (blue) and **both (Additive mixed-selectivity; yellow; Mixed-selectivity with interaction; green).** Non-selective neurons are plotted in grey. Wilcoxon ranksum test was performed between baseline **(-100 to 0 before the cue onset)** and time interval **(800 to 900 ms after the cue onset)** for each channel and position across trials. A non-parametric 2-Way-ANOVA was performed on selective channels with Bonferroni_Holm correction on spatial and task factor **on time interval from 600 to 900 ms after the cue onset** (p<0.01).

### Minor

#### Point 1:

The Introduction and Discussion sections contain some areas that remain unclear (please see specific points below). Additionally, the Introduction does not adequately articulate the gap in the literature that the authors aim to address. The fact that the PFC, particularly the FEF, is known to represent a variety of variables and contingencies is well-established. Therefore, what makes this study's findings novel? What does this research contribute to the already extensive body of knowledge?

**We have now implemented the suggestion of reviewer #2 as follows:** “**While the ability of the PFC—and the FEF in particular—to encode multiple variables and task contingencies is well-established (e.g., visual and motor (Bruce and Goldberg, 1985; Goldberg and Bruce, 1990; Schall, 1991), working memory (Lawrence et al., 2005; Noudoost et al., 2021) and spatial attention (Astrand et al., 2015; Sapountzis et al., 2018) signals), a key unresolved question concerns how such flexible representations are organized to support generalizable, adaptive behavior across tasks. Recent theoretical frameworks propose that flexible cognition may rely on compositional neural codes, in which complex tasks are constructed from modular, reusable components of computation (Frankland and Greene, 2020; Yang et al., 2019a). Recent computational work propose that recurrent neural networks (RNNs), such as those described in prefrontal cortical circuits, utilize shared dynamical motifs—recurring patterns of neural activity implementing specific computations through dynamics such as attractors, decision boundaries, and rotations—to encode task-related information and support flexible multitasking (Driscoll et al., 2024; Yang et al., 2019b). Yet, direct neurophysiological evidence for such compositionality in prefrontal areas remains scarce (Cole et al., 2013; Han et al., 2023). In this study, we address this gap by investigating whether the FEF not only encodes individual task parameters, but also supports higher-order task identity representations that reflect the compositional structure of cognitive operations. By applying demixed principal component analysis to multi-task electrophysiological data in monkeys, we uncover low-dimensional neural states that capture both the identity and proximity of tasks, supporting the notion that the FEF may contribute to cognitive flexibility by encoding abstract, compositional task structures rather than mere sensorimotor mappings.**

***Associated references:***

Astrand, E., Ibos, G., Duhamel, J.-R., Ben Hamed, S., 2015. Differential Dynamics of Spatial Attention, Position, and Color Coding within the Parietofrontal Network. J. Neurosci. Off. J. Soc. Neurosci. 35, 3174–3189. https://doi.org/10.1523/JNEUROSCI.2370-14.2015

Bruce, C.J., Goldberg, M.E., 1985. Primate frontal eye fields. I. Single neurons discharging before saccades. J. Neurophysiol. 53, 603–635.

Cole, M.W., Reynolds, J.R., Power, J.D., Repovs, G., Anticevic, A., Braver, T.S., 2013. Multi-task connectivity reveals flexible hubs for adaptive task control. Nat. Neurosci. 16, 1348–1355. https://doi.org/10.1038/nn.3470

Driscoll, L.N., Shenoy, K., Sussillo, D., 2024. Flexible multitask computation in recurrent networks utilizes shared dynamical motifs. Nat. Neurosci. 27, 1349–1363. https://doi.org/10.1038/s41593-024-01668-6

Frankland, S.M., Greene, J.D., 2020. Concepts and Compositionality: In Search of the Brain’s Language of Thought. Annu. Rev. Psychol. 71, 273–303. https://doi.org/10.1146/annurev-psych-122216-011829

Goldberg, M.E., Bruce, C.J., 1990. Primate frontal eye fields. III. Maintenance of a spatially accurate saccade signal. J. Neurophysiol. 64, 489–508.

Han, C.Z., Donoghue, T., Cao, R., Kunz, L., Wang, S., Jacobs, J., 2023. Using multi-task experiments to test principles of hippocampal function. Hippocampus 33, 646–657. https://doi.org/10.1002/hipo.23540

Lawrence, B.M., White, R.L., Snyder, L.H., 2005. Delay-Period Activity in Visual, Visuomovement, and Movement Neurons in the Frontal Eye Field. J. Neurophysiol. 94, 1498–1508. https://doi.org/10.1152/jn.00214.2005

Noudoost, B., Clark, K.L., Moore, T., 2021. Working memory gates visual input to primate prefrontal neurons. eLife 10, e64814. https://doi.org/10.7554/eLife.64814

Sapountzis, P., Paneri, S., Gregoriou, G.G., 2018. Distinct roles of prefrontal and parietal areas in the encoding of attentional priority. Proc. Natl. Acad. Sci. 115, E8755–E8764. https://doi.org/10.1073/pnas.1804643115

Schall, J.D., 1991. Neuronal activity related to visually guided saccades in the frontal eye fields of rhesus monkeys: comparison with supplementary eye fields. J. Neurophysiol. 66, 559–579. https://doi.org/10.1152/jn.1991.66.2.559

Yang, G.R., Cole, M.W., Rajan, K., 2019a. How to study the neural mechanisms of multiple tasks. Curr. Opin. Behav. Sci., Artificial Intelligence 29, 134–143. https://doi.org/10.1016/j.cobeha.2019.07.001

Yang, G.R., Joglekar, M.R., Song, H.F., Newsome, W.T., Wang, X.-J., 2019b. Task representations in neural networks trained to perform many cognitive tasks. Nat. Neurosci. 22, 297–306. https://doi.org/10.1038/s41593-018-0310-2

I also suggest addressing a contrasting perspective on mixed coding in the FEF within the Discussion (cf. reference below), to provide a balanced counterpoint: Kaleb A Lowe, Wolf Zinke, Joshua D Cosman, Jeffrey D Schall, Frontal eye fields in macaque monkeys: prefrontal and premotor contributions to visually guided saccades, Cerebral Cortex, Volume 32, Issue 22, 15 November 2022, Pages 5083-5107, https://doi.org/10.1093/cercor/bhab533

**We thank reviewer #2 for this suggestion. The following paragraph was additionally added to the discussion:** **“****While our results support the view that the FEF contributes to high-dimensional, task-dependent representations through non-linear mixed selectivity, it is important to consider alternative interpretations of FEF function. Frontal eye field neurons have been shown to be selective to a wide range of signals, ranging from the classical visual and oculomotor features (Bruce and Goldberg, 1985; Goldberg and Bruce, 1990; Schall, 1991), to working memory (Lawrence et al., 2005; Noudoost et al., 2021), spatial attention (Astrand et al., 2015; Sapountzis et al., 2018) and attentional or distractibility states (Amengual et al., 2022; Amengual and Ben Hamed, 2021). These findings have fueled longstanding debates on whether the FEF is primarily a sensory, motor, or cognitive structure. Notably, Lowe et al. (2022) emphasize the distinction between prefrontal and premotor contributions within the FEF, suggesting that a substantial portion of FEF activity may be more closely tied to the premotor control of saccades rather than abstract cognitive variables. They argue for a more parsimonious account of FEF function, centered on its role in sensory-motor transformations. This perspective raises the possibility that some of the task-related modulation observed in our study could reflect dynamic changes in motor preparation or task-dependent oculomotor contingencies. However, the orthogonal task components identified in our dPCA analyses—particularly during the pre-cue period, when motor planning demands are minimal—point to a more abstract, task-identity-related representation. While we acknowledge the sensory-motor grounding of FEF activity, our data suggest an additional layer of flexible, cognitive encoding consistent with the broader role of the prefrontal cortex in executive control. These two perspectives are not mutually exclusive; rather, their integration may offer the most accurate account of FEF function and the richness of its neuronal computations.”**

***Associated references:***

Amengual, J.L., Ben Hamed, S., 2021. Revisiting Persistent Neuronal Activity During Covert Spatial Attention. Front. Neural Circuits 15. https://doi.org/10.3389/fncir.2021.679796

Amengual, J.L., Di Bello, F., Ben Hadj Hassen, S., Ben Hamed, S., 2022. Distractibility and impulsivity neural states are distinct from selective attention and modulate the implementation of spatial attention. Nat. Commun. 13, 4796. https://doi.org/10.1038/s41467-022-32385-y

Astrand, E., Ibos, G., Duhamel, J.-R., Ben Hamed, S., 2015. Differential Dynamics of Spatial Attention, Position, and Color Coding within the Parietofrontal Network. J. Neurosci. Off. J. Soc. Neurosci. 35, 3174–3189. https://doi.org/10.1523/JNEUROSCI.2370-14.2015

Ben Hamed, S., Duhamel, J.-R., Bremmer, F., Graf, W., 2002. Visual receptive field modulation in the lateral intraparietal area during attentive fixation and free gaze. Cereb. Cortex N. Y. N 1991 12, 234–45. https://doi.org/11839598

Bruce, C.J., Goldberg, M.E., 1985. Primate frontal eye fields. I. Single neurons discharging before saccades. J. Neurophysiol. 53, 603–635.

Cole, M.W., Reynolds, J.R., Power, J.D., Repovs, G., Anticevic, A., Braver, T.S., 2013. Multi-task connectivity reveals flexible hubs for adaptive task control. Nat. Neurosci. 16, 1348–1355. https://doi.org/10.1038/nn.3470

Driscoll, L.N., Shenoy, K., Sussillo, D., 2024. Flexible multitask computation in recurrent networks utilizes shared dynamical motifs. Nat. Neurosci. 27, 1349–1363. https://doi.org/10.1038/s41593-024-01668-6

Dubreuil, A., Valente, A., Beiran, M., Mastrogiuseppe, F., Ostojic, S., 2022. The role of population structure in computations through neural dynamics. Nat. Neurosci. 25, 783–794. https://doi.org/10.1038/s41593-022-01088-4

Frankland, S.M., Greene, J.D., 2020. Concepts and Compositionality: In Search of the Brain’s Language of Thought. Annu. Rev. Psychol. 71, 273–303. https://doi.org/10.1146/annurev-psych-122216-011829

Goldberg, M.E., Bruce, C.J., 1990. Primate frontal eye fields. III. Maintenance of a spatially accurate saccade signal. J. Neurophysiol. 64, 489–508.

Han, C.Z., Donoghue, T., Cao, R., Kunz, L., Wang, S., Jacobs, J., 2023. Using multi-task experiments to test principles of hippocampal function. Hippocampus 33, 646–657. https://doi.org/10.1002/hipo.23540

Joiner, W.M., Cavanaugh, J., Wurtz, R.H., 2013. Compression and Suppression of Shifting Receptive Field Activity in Frontal Eye Field Neurons. J. Neurosci. 33, 18259–18269. https://doi.org/10.1523/JNEUROSCI.2964-13.2013

Joiner, W.M., Cavanaugh, J., Wurtz, R.H., 2011. Modulation of shifting receptive field activity in frontal eye field by visual salience. J. Neurophysiol. 106, 1179–1190. https://doi.org/10.1152/jn.01054.2010

Khanna, S.B., Scott, J.A., Smith, M.A., 2020. Dynamic shifts of visual and saccadic signals in prefrontal cortical regions 8Ar and FEF. J. Neurophysiol. 124, 1774–1791. https://doi.org/10.1152/jn.00669.2019

Lawrence, B.M., White, R.L., Snyder, L.H., 2005. Delay-Period Activity in Visual, Visuomovement, and Movement Neurons in the Frontal Eye Field. J. Neurophysiol. 94, 1498–1508. https://doi.org/10.1152/jn.00214.2005

Lowe, K.A., Zinke, W., Cosman, J.D., Schall, J.D., 2022. Frontal eye fields in macaque monkeys: prefrontal and premotor contributions to visually guided saccades. Cereb. Cortex 32, 5083–5107. https://doi.org/10.1093/cercor/bhab533

Márton, C.D., Gagnon, L., Lajoie, G., Rajan, K., 2022. Efficient and robust multi-task learning in the brain with modular latent primitives. https://doi.org/10.48550/arXiv.2105.14108

Noudoost, B., Clark, K.L., Moore, T., 2021. Working memory gates visual input to primate prefrontal neurons. eLife 10, e64814. https://doi.org/10.7554/eLife.64814

Sapountzis, P., Paneri, S., Gregoriou, G.G., 2018. Distinct roles of prefrontal and parietal areas in the encoding of attentional priority. Proc. Natl. Acad. Sci. 115, E8755–E8764. https://doi.org/10.1073/pnas.1804643115

Schall, J.D., 1991. Neuronal activity related to visually guided saccades in the frontal eye fields of rhesus monkeys: comparison with supplementary eye fields. J. Neurophysiol. 66, 559–579. https://doi.org/10.1152/jn.1991.66.2.559

Yang, G.R., Cole, M.W., Rajan, K., 2019a. How to study the neural mechanisms of multiple tasks. Curr. Opin. Behav. Sci., Artificial Intelligence 29, 134–143. https://doi.org/10.1016/j.cobeha.2019.07.001

Yang, G.R., Joglekar, M.R., Song, H.F., Newsome, W.T., Wang, X.-J., 2019b. Task representations in neural networks trained to perform many cognitive tasks. Nat. Neurosci. 22, 297–306. https://doi.org/10.1038/s41593-018-0310-2

Zirnsak, M., Moore, T., 2014. Saccades and shifting receptive fields: anticipating consequences or selecting targets? Trends Cogn. Sci. 18, 621–628. https://doi.org/10.1016/j.tics.2014.10.002

#### Point 2:

2) "Each of these tasks recruited specific cognitive functions: endogenous attention, exogenous attention and spatial working memory." - as has been argued previously (and agreed upon by the authors), the spatial working memory is present in all 3 tasks, the main differences beyond attentional demands are the presence of visual stimuli placeholders during the delay in two attention tasks, and the manual response vs saccades. I think it needs to be mentioned already in the Introduction.

**The introduction has been modified as follows: “**[…] Each of these tasks recruited specific cognitive functions: endogenous attention, exogenous attention and spatial working memory. **While all tasks involved spatial working memory processes, they differed in their attentional demands and responses. The attentional tasks included specific landmark presentations during the delay and required a manual response to the target, whereas the spatial working memory task required a saccadic response to the target.”**

"While monkeys were performing these tasks in independent blocks or trials," - blocks ***of*** trials.

**The introduction has been corrected: "**While monkeys were performing these tasks in independent blocks **of** trials**”**

#### Point 3:

3) "…, dense recording probes were used to record multi-unit activity (MUA)…" - 24-contact Plexon U-probes with 250 micron distance between sites are not really dense recording probes (unlike e.g. Neuropixels where contacts are very close and each neuron can be seen on several channels - here the channels are independent).

**We have now changed the text as follows: “**[…] recording probes were used to record multi-unit activity (MUA) from both frontal eye fields (FEF), […]”

#### Point 4:

4) "This result suggests that task identity and spatial information are not encoded independently in absolute terms such that the specific state of one variable influences the specific computations implemented by the other variable." - I cannot fully understand this sentence, in particular the "such that" logical transition - do you mean that two types of information are ***not encoded independently*** so that one variable does influence the other?

**This has now been rephrased as follows: “**[…] This result suggests that, **although the FEF encodes some aspects of task identity and spatial information independently, some other aspects of task identity influence how spatial information is coded,** such that the specific state of task identity influences the computations associated with position coding.”

Point 5:

5) Note that the next (last) paragraph of the Introduction seems to claim the opposite: "In addition, spatial information is represented ***independently*** from task-related information, indicating that task-identity is represented at the same hierarchical level as specific task parameters such as position.

**This has now been rephrased as follows: “**[…] All in all, these results suggest that prefrontal cortex encodes sensory information and task-identity in a high-dimensional neural representation. In addition, **some aspects of** spatial information **are** represented independently from task-related information, **while other aspects of task-related information influence spatial information coding,** **suggesting** that task-identity is represented at the same hierarchical level as specific task parameters such as position.”

#### Point 6:

6) "At the fundamental level, it will be important to pursue on this work in order to better understand the specific dimensions that the prefrontal cortex uses to represent task identity and achieve perceptual representation stability across tasks. At the translational level, this work opens new venues for the implementation of brain computer interface technologies based on machine learning algorithms able to extract complex cognitive information from realtime PFC recordings." - but in the next paragraph, this statement is again repeated:

"At the fundamental level, it will be important to pursue on this work in order to better understand the specific dimensions that the prefrontal cortex uses to represent task identity. At the translational level, this work opens new venues for the implementation of brain computer interface technologies based on machine learning algorithms able to extract complex cognitive information from real-time PFC recordings."

**The entire paragraph was indeed duplicated. We apologize for this oversight.**

#### Point 7:

7) "These three tasks presented a very similar structure (the onset of a cue was followed by a target stimuli)." - and spatially-contingent manual or saccade response?

**We have now clarified this point as follows: “**[…] These three tasks presented a very similar structure (the onset of a cue was followed by a target stimuli). **However, the attentional tasks** **required a manual response while the working memory task required a saccadic response and …”**

#### Point 8:

8) "The memory guided saccade task mostly recruited spatial working memory as well as spatially accurate sensorimotor transformation processes." - The attention tasks also required sensorimotor transformation: visual target detection ---> manual response. Please amend this statement.

**This was rephrased as follows: “**[…] The two attentional tasks recruited endogenous spatial attention, exogenous spatial attention processes in addition to distractor suppression mechanisms**,** spatial working memory processes **and non-spatialized** **sensorimotor transformation processes**. The memory guided saccade task mostly recruited spatial working memory as well as spatially accurate sensorimotor transformation processes. In the following, we will refer to attention and spatial working memory as the distinguishing features between the two sets of tasks for simplicity.”

#### Point 9:

9) "We found that, at the neural level, the dynamics of the activity of the FEF cells was cued was different across tasks, suggesting a different encoding of the sensory information as a function of the task being performed." - "was cued was different" does not work. More importantly, I am not at all sure how is the dynamics addressed in this study?

**This was rephrased as follows: “**[…] We found that, at the neural level, **the activity of FEF cells varied across tasks, suggesting that sensory information is encoded differently depending on the task being performed.”**

#### Point 10:

10) "We found that the FEF encoded task identity in two sources of task-related variability
(independently from the processing of sensory information) that segregated in orthogonal components that conformed a two-dimensional space, …" - "that segregated … that conformed" does not make sense.

**This was rephrased as follows:** “[…] We found that the FEF encoded task identity in two sources of task-related variability (independently from the processing of sensory information**). These segregated** in orthogonal components that conformed a two-dimensional space, …”

Point 11:

11) "…high dimensional representation of the neuronal population" - no, as the authors wrote in the paragraph above, it is "high dimensional representation of sensory information".

**This was corrected as follows: “**[…] This is achieved thanks to a high dimensional representation of the **sensory information** which endows the FEF with a high degree of flexibility.”

#### Point 12:

12) "Source data for the figures and associated code can be downloaded at the following link:"
<https://osf.io/z8eh9/?view_only=a445fa5522924f709cca985b97ae7e9e>

**Apologies, the permissions to access scripts and data for non-contributors to the project were not set properly. Here is a corrected link:** [**https://osf.io/z8eh9/**](https://osf.io/z8eh9/)

**The project has now been set as public.**

**Scripts can be downloaded from Scripts/Files/OSF Storage**

**Data can be downloaded from Data/Files/OSF Storage**

**Zipped Project can be downloaded from ZippedProject/Files/OSF Storage**

#### Point 13:

13) "Supplementary material M2: … In contrast with other methods such as decoding or PCA, the analysis of neuronal variability that we implemented in this work relies on demixed principal component analysis (dPCA, see below). This method has been sho" - missing the end of the sentence?

**There was a copy paste error in this paragraph. This is now corrected.**
