# Supplementary material for: The prefrontal cortex encodes task-identity information and flexibly adjusts its sensory processes as a function of the specific ongoing task
Source: PLoS Biol. 2025 Aug 26;23(8):e3003353. doi: 10.1371/journal.pbio.3003353 (PMC12463330; doi:10.1371/journal.pbio.3003353)
Supplement: Attachment — Submitted filename: Response_to_Reviewer_R3_final_23-02960R4.docx [file pbio.3003353.s019.docx]

**We would like to thank the reviewers and the editor for their positive assessment of our work and the constructive comments. We believe that this paper is now improved thanks to their feedback on our work.**

**Please find below a point-by-point response to the reviewers’ comments. Reviewers’ questions are in** black normal fonts**. Responses and changes made to original version of the manuscript are in bold. Text from the original version of the manuscript is in** normal blue font**.**

Reviewer #2:

1.Please systematically summarize your data sample: how many sessions, how many valid channels, and most importantly, how many sessions/channels have been successfully spike-sorted and how many single units have been extracted and analyzed.

**We thank the reviewer for this comment and apologize for the lack of clarity. We have now implemented this information in the method section of the manuscript.**

**p.6** : […] Within any recording session **(M1: n=7; M2, n=5),** the monkeys performed the three tasks consecutively. The order of the attentional tasks was changed randomly between sessions, while the memory-guided saccade task was always presented last, as monkeys displayed a strong behavioural preference for this task -though with a low success rate due to not sufficiently precise saccades- and refused to work on the attentional tasks when these were presented after the memory guided saccade task.

**p.6:** […] Simultaneous recordings in the two FEFs were carried out using two 24-contact Plexon U-probes. The contacts had an interspacing distance of 250 µm. Neural data was acquired with PlexonOmniplex® neuronal data acquisition system (Omniplex). The data was amplified by a factor of 400 and digitized at 40,000 Hz. The neuronal data was low-cut filtered at 300 Hz. All analyses were performed on the MUA recorded from each of the 48 recording contacts **(M1: number of sessions =7, total number of channels= 336; M2: number of sessions =5, total number of channels=240).** A threshold was applied independently for each recording contact and before the actual task-related recordings started, such that the crossing of this threshold resulted in multiunit spike trains. This threshold corresponded to 3 standard deviations from mean activity during unconstrained eye movements by the monkeys while exploring a blank screen. This threshold was confirmed during the offline analyses to optimize single unit activity extraction for each channel. This readjustment impacted MUA final thresholding values. **Spike sorting was performed, on a subsample of channels (M1: n=94; M2: n=69),** using the Offline Sorter (Plexon inc.), on the broadband time signal (bandpass filtered between 300Hz and 3000Hz) of the entire session prior to assigning the signal to the different tasks. Only spikes that could be unambiguously assigned to a same neuron throughout all the tasks were used for the SUA-based analyses **(M1: n=43; M2: n=15).** The “Clusters vs. Time” view was used (using the most informative principal component pairs) to check how the waveform changed as a function of time throughout the session. Only units that could be continuously tracked through the entire session, with no drift or a smooth continuous drift in PCA cluster attributes were used for the SUA-based analyses. Examples of spike sorted waveforms tracked in the three different tasks are presented in supplementary Figure 1. All further analyses of the data were performed in Matlab (The Mathworks INC., Natick, Massachussetts).

2. My sincere apologies, but even after all error fixes and explanations, I still cannot follow the logic of different baselines: -500 to -300 before the cue onset vs -500 to 0 before the cue onset (e.g. both used in the Figure 2)? What is the pre-cue epoch: "pre-cue epoch (-500 to 0ms prior to cue presentation, supplementary Figure 5B)" -- page 12, or "pre-cue period (200 ms before cue onset to cue onset)" -- page 14? Then there is also another period “averaged in time interval -300 to -100ms in pre cue” in Suppl. Figure 8 and 9…

**In spite of our diligence on this, there were mistakes in updating the main text of the manuscript -but not in the analyses. We apologize for this. We used as baseline for all analyses the Intervale -500 to -200 ms before the cue onset.**

**Additionally, we confirm that the precue analyses were performed on the interval -200 to 0 ms before the cue onset. This is now clarified in the manuscript.**

**Text, P:11:**

Figure 2. Mixed selectivity corresponds to the ability of a neuronal population to encode simultaneously independent sources of information: (A) Firing rate of a representative MUA aligned on the cue (200 ms before the cue onset to 1000 ms after the cue onset) in exogenous task (blue), endogenous task (green) and memory guided saccade task (red) for preferred (solid line) and non-preferred (dash line) position. The data were baseline corrected (-500 to **-200 ms** before cue onset) on individual trials and channel. The gray box corresponds to the delay period during which neuronal activities were analyzed for subplots C and D. (B) Mean firing rate of the MUA neuronal population in the three tasks aligned on cue presentation (200 ms before the cue onset to 1000 ms after the cue onset) in the exogenous task (blue), the endogenous task (green) and the memory guided saccade task (red) for preferred (full line) and non-preferred (dash line) position. The data were baseline corrected (-500 to **-200 ms** before cue onset) on individual trial and channel. Gray box corresponds to the delay period during which neuronal activities were analyzed for subplots C and D. (C) Venn diagram representing the proportion of cells showing a spatial tuning in each of the three tasks. (D) Pie chart corresponding to the proportion of neurons tuned to only position (red), task (blue) and both (Additive mixed-selectivity; yellow; Mixed-selectivity with interaction; green). Non-selective neurons are plotted in gray. Wilcoxon ranksum test was performed between baseline (-100 to 0 before the cue onset) and time interval (800 to 900 ms after the cue onset) for each channel and position across trials. A non-parametric 2-Way-ANOVA was performed on selective channels with Bonferroni-Holm correction on spatial and task factors on time interval from 600 to 900 ms after the cue onset (p<0.01).

**Text, P:12:**

[…] For MUA, selectivity did not depend on the post-cue analysis window (MUA: supplementary Figure 5A), and only marginally did so for SUA (SUA: supplementary Figure 5B). As expected, no position-related nor mixed-selectivity MUAs or SUAs were identified during the pre-cue epoch **(-200** to 0ms prior to cue presentation, supplementary Figure 5B).

**Text, P:13:**

**Figure 3.** Spatial tuning of mixed-selectivity cells. (A) MUA example, with mixed-selectivity, and finely-tuned receptive field. Plots represent firing rates (average across trials +/- s.e.) baseline corrected (**-500 to -200 ms** before cue onset) on individual trial: Exo, blue, Endo, green, Sacc, red. Firing rates at preferred spatial position are significantly different from the other spatial locations for all tasks (p<0.005 or better). (B) MUA example, with mixed-selectivity, and board receptive field. All else as in (A). Firing rates at the two preferred spatial positions are significantly different from the other spatial locations for all tasks (p<0.05 or better), except for the Ipsi versus Contra and Diagonal comparison in the Sacc task. (C) Population spatial tuning of mixed-selectivity MUAS with no interaction. Plots represent firing rates (average across MUAs +/- s.e.) baseline corrected (**-500 to -200 ms** before cue onset) on individual trial: Exo, blue, continuous, MUAs with broad receptive fields (RF), dashed, MUAs with finely-tuned receptive fields, Endo, green, Sacc, red. Firing rates at preferred spatial position are significantly different from the other spatial locations for all tasks and conditions (p<0.005 or better). (D) Population spatial tuning of mixed-selectivity MUAS with interaction. All else as in (D). Firing rates at preferred spatial position are significantly different from the other spatial locations for all tasks and conditions except for the firing rates of the MUAs with broad receptive fields in the Exo task (p<0.001 or better). Firing rates at Ipsi spatial position are significantly different from the Contra and Diag spatial locations for all tasks and conditions except for the Sacc task (p<0.005 or better). Preferred indicates the spatial location eliciting the highest variation in firing rate from baseline. Ipsi indicates the stimulated spatial position that falls in the same hemifield as the Preferred location. Contra indicates the stimulated spatial position that falls in the contralateral hemifield as the Preferred location. Diag indicates the stimulated spatial position that falls in the contralateral hemifield as the Preferred location, diagonally to it.

**Text, P:27:**

**Supplementary Figure 2.** Mixed selectivity corresponds to the ability of a neuronal population to encode simultaneously independent sources of information: (A) Firing rate of a representative SUA aligned on the cue (200 ms before the cue onset to 1000 ms after the cue onset) in exogenous task (blue), endogenous task (green) and memory guided saccade task (red) for preferred (solid line) and non-preferred (dash line) position. The data were baseline corrected **(-500 to -200 ms** before cue onset) on individual trial and channel. Gray box corresponds to the delay period during which neuronal activities were analyzed for subplots C and D. (B) Firing rate of a representative SUA (same as in A) aligned on the target (500 ms before the target onset to 500 ms after the target onset). All else as in A. (C) Mean firing rate of the SUA neuronal population in the three tasks aligned on cue presentation (200 ms before the cue onset to 1000 ms after the cue onset) in the exogenous task (blue), the endogenous task (green) and the memory guided saccade task (red) for preferred (full line) and non-preferred (dash line) position. The data were baseline corrected (**-500 to -200 ms** before cue onset) on individual trial and channel. Gray box corresponds to the delay period during which neuronal activities were analyzed for subplots C and D. (D) Firing rate of the SUA neuronal population (same as in C) aligned on the target (500 ms before the target onset to 500 ms after the target onset). All else as in C. (E) Venn diagram representing the proportion of cells showing a spatial tuning in each of the three tasks. (F) Pie chart corresponding to the proportion of neurons tuned to only position (red), task (blue) and both (Additive mixed-selectivity; yellow; Mixed-selectivity with interaction; green). Non-selective neurons are plotted in grey. Wilcoxon ranksum test was performed between baseline (-100 to 0 before the cue onset) and time interval (800 to 900 ms after the cue onset) for each channel and position across trials. A non-parametric 2-Way-ANOVA was performed on selective channels with Bonferroni_Holm correction on spatial and task factor on time interval from 600 to 900 ms after the cue onset (p<0.01).

**Supplementary Figure 2.** (A) Firing rate of a representative MUA (same as in Figure 2) aligned to target onset (-500 ms before target onset to 600 ms after target onset) averaged across trials of the exogenous attention task (blue), the endogenous attention task (green) and the memory guided saccade task (red) for preferred (solid line) and least-preferred (dash line) positions. **The data were baseline corrected (-500 to -200 ms before cue onset) on individual trials and channels.** (B) Mean firing rate across the neuronal population (MUA) aligned on the target (-500 ms before target onset to 600 ms after target onset) as a function of tasks and positions (same as in A). **The data were baseline corrected (-500 to -200 ms before cue onset) on individual trials and channels.**

**Text, P:28:**

**Supplementary Figure 7.** Projection of MUA population activity onto the first and second principal components extracted with a PCA analysis. Each point corresponds to the projection of the activity in each trial (averaged in the time interval **-200** **to 0 ms** pre Cue, each circle thus corresponds to one trial) in each task (Exogenous task, blue; Endogenous task; green; Memory saccade task, red). Each plot corresponds to a different session. Plots 1 to 7 are from monkey 1 and plots 8 to 12 are from monkey 2.

Supplementary Figure 9. Projection of MUA onto the first and second demixed principal components extracted with a dPCA analysis. Each point corresponds to the projection of the activity in each trial (averaged in the time interval -200 to 0 ms pre-Cue, each circle thus corresponds to one trial) in each task (Exogenous task, blue; Endogenous task; green; Memory saccade task, red). Each plot corresponds to a different session. Plots 1 to 7 are from monkey 1 and plots 8 to 12 are from monkey 2.

3. It would be good to see the firing rate of MUA and SUA as a function of time in the session

**We have now added a new figure S10, presenting the raster plots for each of the three tasks, aligned on cue and aligned on target for a representative MUA example and a representative SUA example. This figure and its legend are duplicated below. For the MUA example, the session started with the Endogenous task, followed by the Exogenous task, then the Working Memory task. Notably, in the Endogenous task, the neural response to the cue was delayed, likely due to the cue being presented outside the receptive field, unlike in the Exogenous and Working Memory tasks. Please note, that in the endogenous task, the cue is not presented in the center of the receptive field. For the SUA example, the session started with the Exogenous task, followed by the Endogenous task, then the Working Memory task.**

**Supplementary Figure 10:** Raster plots of representative MUA and SUA examples.
(A) Raster plot of a representative MUA channel showing spike activity throughout an entire session (First task in session, Endo: green; Second task, Exo: blue; Third task, Sacc: red). Firing rates are shown below the raster plots, aligned to cue onset (−200 ms to +1000 ms) and to target onset (−500 ms to +500 ms). (B) Raster plot of a representative SUA example, as in (A). First task in session, Exo: blue; Second task, Endo: green; Third task, Sacc: red. The inset shows the waveform of the selected spike-sorted unit from the same channel.

**In addition, in a text associated with supplementary figure 10, we describe the following: “Focusing on spiking activity during the precue period (−200 to 0 ms relative to cue onset), we observed significant variation in firing rates across the three tasks, consistent with the task-related components identified by the dPCA during this epoch (Figure 4). To assess whether these differences might be explained by variations in trial-to-trial spiking variability—potentially due to recording instability—we computed the coefficient of variation (CV) of spike counts across trials, separately for each MUA and SUA, for each task and session. For MUAs, median CVs were: Exo = 0.2460 ± 0.0185, Endo = 0.2224 ± 0.0172, and Sacc = 0.2535 ± 0.0201. For SUAs, median CVs were: Exo = 0.8687 ± 0.0572, Endo = 1.1808 ± 0.0957, and Sacc = 0.9688 ± 0.0625. Non-parametric pairwise comparisons revealed no significant differences in CV across tasks for either MUAs or SUAs (all p > 0.05). These findings indicate that the observed task-dependent changes in spiking rates cannot be attributed to differences in inter-trial variability. Instead, they support the conclusion that task identity modulates neural activity during the precue period in a structured and reliable manner.”**

**In the main text, this is called as follows: “**[…] Thus, the task-dPCA component cannot distinguish between first and second task in the session, while it can distinguish between Endo and Exo attentional tasks. **These precue effects were not driven by recording instability (Supplementary Figure 10 and associated text)”.**

4. Concerning the new, very important for illustrations of mixed selectivity, Figure 3 and the associated text: it would be interesting to see the examples of linear and non-linear (i.e. with interaction) mixed selectivity MUA, and also corresponding examples of SUA. The population averages of MUA in 3C and D do not make a convincing case as they do not look that much different from each other.

**We appreciate the comment of the reviewer and identified an error when extracting the MUA mixed population with and without interaction. The corrected figure three is now appended below as well as the corrected category distributions.**

**Figure 3:**

**Figure 3.** Spatial tuning of mixed-selectivity cells. (A) MUA example, with mixed-selectivity, and finely-tuned receptive field. Plots represent firing rates (average across trials +/- s.e.) baseline corrected (**-500 to -200 ms** before cue onset) on individual trial: Exo, blue, Endo, green, Sacc, red. Firing rates at preferred spatial position are significantly different from the other spatial locations for all tasks (p<0.005 or better). (B) MUA example, with mixed-selectivity, and board receptive field. All else as in (A). Firing rates at the two preferred spatial positions are significantly different from the other spatial locations for all tasks (p<0.05 or better), except for the Ipsi versus Contra and Diagonal comparison in the Sacc task. (C) Population spatial tuning of mixed-selectivity MUAS with no interaction. Plots represent firing rates (average across MUAS +/- s.e.) baseline corrected (**-500 to -200 ms** before cue onset) on individual trial: Exo, blue, continuous, MUAs with broad receptive fields (RF), dashed, MUAs with finely-tuned receptive fields, Endo, green, Sacc, red. Firing rates at preferred spatial position are significantly different from the other spatial locations for all tasks and conditions (p<0.005 or better). (D) Population spatial tuning of mixed-selectivity MUAS with interaction. All else as in (D). Firing rates at preferred spatial position are significantly different from the other spatial locations for all tasks and conditions except for the firing rates of the MUAs with broad receptive fields in the Exo task (p<0.001 or better). Firing rates at Ipsi spatial position are significantly different from the Contra and Diag spatial locations for all tasks and conditions except for the Sacc task (p<0.005 or better). Preferred indicates the spatial location eliciting the highest variation in firing rate from baseline. Ipsi indicates the stimulated spatial position that falls in the same hemifield as the Preferred location. Contra indicates the stimulated spatial position that falls in the contralateral hemifield as the Preferred location. Diag indicates the stimulated spatial position that falls in the contralateral hemifield as the Preferred location, diagonally to it.

**Text, P:13:**

[…] However, such a response pattern was **predominantly identified in the Exo task (Mixed-selectivity without interaction: Exo: 18%, Endo: 5%, Sacc: 5%; Mixed-selectivity with interaction, Exo: 46%, Endo: 24%, Sacc: 18%).** […]

[…] For the vast majority of mixed-selectivity MUAs without interaction, the receptive fields shared at least one spatial position across each pair of tasks (Exo-Endo: 98%; Exo-Sacc: **95**%; Endo-Sacc: **94**%). This was also the case for the receptive fields of mixed-selectivity MUAs with interaction, with the marked exception of the Endo-Sacc comparison (Exo-Endo: **96**%; Exo-Sacc: **93**%; Endo-Sacc: **69**%). […]

**We have additionally added single MUA and SUA examples showing finely-tuned receptive fields or broad receptive fields (Supplementary Figure 6).**

**Supplementary Figure 6:**

**Supplementary Figure 6.** Spatial tuning of mixed-selectivity cells. (A) MUA example, with mixed-selectivity without interaction, and finely-tuned receptive field (Exo, blue, Endo, green, Sacc, red). (B) MUA example, with mixed-selectivity without interaction, and board receptive field. (C) MUA example, with mixed-selectivity with interaction, and finely-tuned receptive field (Exo, blue, Endo, green, Sacc, red). (D) MUA example, with mixed-selectivity with interaction, and board receptive field. (E)-(H) Same as (A)-(D) for SUA example.

"Understanding why these shifts seem to be specific to mixed-selectivity MUAs with interaction requires further investigations." - I am not sure if I am following this statement. Shouldn't that be the case by definition? If there is an RF shift, doesn't the unit have to show mixed selectivity?

**We have now removed this sentence.**

5. “Single MUA” confusion expression and would suggest using “an example MUA” in corresponding places.

**We thank the reviewer for this comment and agree with the suggestion. This has now been changes throughout the manuscript and in figures 3, S6.**

**Figure 3.** Spatial tuning of mixed-selectivity cells. (A) **MUA example**, with mixed-selectivity, and finely-tuned receptive field. Plots represent firing rates (average across trials +/- s.e.) baseline corrected (**-500 to -200 ms** before cue onset) on individual trial: Exo, blue, Endo, green, Sacc, red. Firing rates at preferred spatial position are significantly different from the other spatial locations for all tasks (p<0.005 or better). (B) **MUA example**, with mixed-selectivity, and board receptive field. All else as in (A). Firing rates at the two preferred spatial positions are significantly different from the other spatial locations for all tasks (p<0.05 or better), except for the Ipsi versus Contra and Diagonal comparison in the Sacc task. (C) Population spatial tuning of mixed-selectivity MUAS with no interaction. Plots represent firing rates (average across MUAS +/- s.e.) baseline corrected (**-500 to -200 ms** before cue onset) on individual trial: Exo, blue, continuous, MUAS with broad receptive fields (RF), dashed, MUAS with finely-tuned receptive fields, Endo, green, Sacc, red. Firing rates at preferred spatial position are significantly different from the other spatial locations for all tasks and conditions (p<0.005 or better). (D) Population spatial tuning of mixed-selectivity MUAS with interaction. All else as in (D). Firing rates at preferred spatial position are significantly different from the other spatial locations for all tasks and conditions except for the firing rates of the MUAs with broad receptive fields in the Exo task (p<0.001 or better). Firing rates at Ipsi spatial position are significantly different from the Contra and Diag spatial locations for all tasks and conditions except for the Sacc task (p<0.005 or better). Preferred indicates the spatial location eliciting the highest variation in firing rate from baseline. Ipsi indicates the stimulated spatial position that falls in the same hemifield as the Preferred location. Contra indicates the stimulated spatial position that falls in the contralateral hemifield as the Preferred location. Diag indicates the stimulated spatial position that falls in the contralateral hemifield as the Preferred location, diagonally to it.

**Supplementary Figure 6.** Spatial tuning of mixed-selectivity cells. (A) **MUA example**, with mixed-selectivity without interaction, and finely-tuned receptive field (Exo, blue, Endo, green, Sacc, red). (B) **MUA example**, with mixed-selectivity without interaction, and board receptive field. (C) **MUA example**, with mixed-selectivity with interaction, and finely-tuned receptive field (Exo, blue, Endo, green, Sacc, red). (D) **MUA example**, with mixed-selectivity with interaction, and board receptive field. The shift in position is different between the three tasks as compared to mixed-selectivity without interaction (A) and (B). (E)-(H) Same as (A)-(D) for SUA example.
